# Cross-niche metabolite–microbiome interactions orchestrate systemic soybean resistance to *Fusarium* root rot

**DOI:** 10.1093/ismejo/wrag080

**Published:** 2026-03-27

**Authors:** Qi Liu, Lang Cheng, Enxi Zhang, Li Ling, Weiyi Tan, Suwen Liang, Canwei Shu, Qibin Ma, Shuai Zhao, Jian Wei, Yingxiang Wang, Hai Nian, Yanbo Cheng, Tengxiang Lian

**Affiliations:** Guangdong Basic Research Center of Excellence for Precise Breeding of Future Crops, Guangdong Laboratory for Lingnan Modern Agriculture, Guangdong Provincial Key Laboratory for the Development Biology and Environmental Adaptation of Agricultural Organisms, South China Institute for Soybean Innovation Research, College of Agriculture, South China Agricultural University, No.483 Wushan Road, Guangzhou, Guangdong 510642, China; Guangdong Basic Research Center of Excellence for Precise Breeding of Future Crops, Guangdong Laboratory for Lingnan Modern Agriculture, Guangdong Provincial Key Laboratory for the Development Biology and Environmental Adaptation of Agricultural Organisms, South China Institute for Soybean Innovation Research, College of Agriculture, South China Agricultural University, No.483 Wushan Road, Guangzhou, Guangdong 510642, China; Guangdong Basic Research Center of Excellence for Precise Breeding of Future Crops, Guangdong Laboratory for Lingnan Modern Agriculture, Guangdong Provincial Key Laboratory for the Development Biology and Environmental Adaptation of Agricultural Organisms, South China Institute for Soybean Innovation Research, College of Agriculture, South China Agricultural University, No.483 Wushan Road, Guangzhou, Guangdong 510642, China; Guangdong Basic Research Center of Excellence for Precise Breeding of Future Crops, Guangdong Laboratory for Lingnan Modern Agriculture, Guangdong Provincial Key Laboratory for the Development Biology and Environmental Adaptation of Agricultural Organisms, South China Institute for Soybean Innovation Research, College of Agriculture, South China Agricultural University, No.483 Wushan Road, Guangzhou, Guangdong 510642, China; Guangdong Basic Research Center of Excellence for Precise Breeding of Future Crops, Guangdong Laboratory for Lingnan Modern Agriculture, Guangdong Provincial Key Laboratory for the Development Biology and Environmental Adaptation of Agricultural Organisms, South China Institute for Soybean Innovation Research, College of Agriculture, South China Agricultural University, No.483 Wushan Road, Guangzhou, Guangdong 510642, China; Guangdong Basic Research Center of Excellence for Precise Breeding of Future Crops, Guangdong Laboratory for Lingnan Modern Agriculture, Guangdong Provincial Key Laboratory for the Development Biology and Environmental Adaptation of Agricultural Organisms, South China Institute for Soybean Innovation Research, College of Agriculture, South China Agricultural University, No.483 Wushan Road, Guangzhou, Guangdong 510642, China; Guangdong Basic Research Center of Excellence for Precise Breeding of Future Crops, Guangdong Laboratory for Lingnan Modern Agriculture, Guangdong Provincial Key Laboratory for the Development Biology and Environmental Adaptation of Agricultural Organisms, South China Institute for Soybean Innovation Research, College of Agriculture, South China Agricultural University, No.483 Wushan Road, Guangzhou, Guangdong 510642, China; Guangdong Basic Research Center of Excellence for Precise Breeding of Future Crops, Guangdong Laboratory for Lingnan Modern Agriculture, Guangdong Provincial Key Laboratory for the Development Biology and Environmental Adaptation of Agricultural Organisms, South China Institute for Soybean Innovation Research, College of Agriculture, South China Agricultural University, No.483 Wushan Road, Guangzhou, Guangdong 510642, China; State Key Laboratory of Desert and Oasis Ecology, Xinjiang Institute of Ecology and Geography, Chinese Academy of Sciences, Urumqi, Xinjiang 830011, China; Northern Key Laboratory of Saline-tolerant Soybean Breeding, Ministry of Agriculture and Rural Areas, Jilin Agricultural University, Changchun, Jilin 130118, China; Guangdong Basic Research Center of Excellence for Precise Breeding of Future Crops, Guangdong Laboratory for Lingnan Modern Agriculture, Guangdong Provincial Key Laboratory for the Development Biology and Environmental Adaptation of Agricultural Organisms, South China Institute for Soybean Innovation Research, College of Agriculture, South China Agricultural University, No.483 Wushan Road, Guangzhou, Guangdong 510642, China; Guangdong Basic Research Center of Excellence for Precise Breeding of Future Crops, Guangdong Laboratory for Lingnan Modern Agriculture, Guangdong Provincial Key Laboratory for the Development Biology and Environmental Adaptation of Agricultural Organisms, South China Institute for Soybean Innovation Research, College of Agriculture, South China Agricultural University, No.483 Wushan Road, Guangzhou, Guangdong 510642, China; Northern Key Laboratory of Saline-tolerant Soybean Breeding, Ministry of Agriculture and Rural Areas, Jilin Agricultural University, Changchun, Jilin 130118, China; Guangdong Basic Research Center of Excellence for Precise Breeding of Future Crops, Guangdong Laboratory for Lingnan Modern Agriculture, Guangdong Provincial Key Laboratory for the Development Biology and Environmental Adaptation of Agricultural Organisms, South China Institute for Soybean Innovation Research, College of Agriculture, South China Agricultural University, No.483 Wushan Road, Guangzhou, Guangdong 510642, China; Guangdong Basic Research Center of Excellence for Precise Breeding of Future Crops, Guangdong Laboratory for Lingnan Modern Agriculture, Guangdong Provincial Key Laboratory for the Development Biology and Environmental Adaptation of Agricultural Organisms, South China Institute for Soybean Innovation Research, College of Agriculture, South China Agricultural University, No.483 Wushan Road, Guangzhou, Guangdong 510642, China

**Keywords:** Soybean, root rot, SynComs strategies, phyllosphere-mediated protection, metabolic remodulating

## Abstract

*Fusarium* root rot, predominantly caused by *Fusarium falciforme*, poses a significant threat to soybean productivity globally. Microbiome-based strategies offer sustainable alternatives, but the mechanisms underlying multi-niche interactions remain elusive. Here, we found that a tolerant soybean cultivar (GXD2) coordinates spatially resolved metabolite signals to recruit beneficial microbes across the rhizosphere, root endosphere, and leaf endosphere. Specifically, formononetin and maltol selectively enrich *Bacillus* and *Massilia* in the rhizosphere; arctigenin and isovanillic acid recruit *Bacillus* and *Streptomyces* to the root endosphere; and flavonoids such as diosmetin attract *Penicillium* and *Aspergillus* to the leaf endosphere. Leveraging these interactions, we constructed different types of synthetic communities (SynComs) via top–down (host-selected taxa) and bottom–up (antagonist-based) strategies. Both SynComs suppressed root rot in susceptible cultivars, with foliar application of top–down SynComs significantly enhancing shoot growth. Transcriptomics revealed distinct modes of action: that top–down SynComs activated mitogen-activated protein kinase (MAPK)-linked terpenoid and flavonoid pathways, whereas bottom–up SynComs primarily modulated host carbon–nitrogen allocation, effectively limiting pathogen resources. Our findings unveil a “metabolite-mediated, multi-niche collaborative defense” model, presenting a robust framework for microbiome-based disease management and paving the way toward sustainable crop protection strategies.

## Introduction

Soybean (*Glycine max*), a major global crop, faces substantial yield reductions due to *Fusarium* root rot (FRR), predominantly caused by *Fusarium falciforme* [1]. This fungal pathogen significantly threatens soybean production worldwide, particularly under monoculture systems where pathogen virulence and persistence are intensified [2, 3]. Current management practices rely primarily on chemical fungicides, yet these approaches are increasingly compromised by pathogen resistance development, environmental concerns, and regulatory limitations [4, 5]. Thus, sustainable alternatives, particularly microbiome-based solutions, have become urgent priorities in plant protection research.

Plants naturally harbor diverse microbiota across multiple niches, including rhizosphere, root endosphere, and phyllosphere, which collectively form intricate networks capable of enhancing host resilience to various biotic stresses [6]. Recent studies reveal that plants actively recruit specific beneficial microbes through selective metabolite secretion, establishing protective microbial consortia [7, 8]. However, empirical support for this recruitment–mutualism model remains largely root-centric [9, 10]. How plants spatially coordinate microbiota assembly across distinct compartments to defend against pathogens remains largely unexplored. Furthermore, the molecular mechanisms underlying cross-compartment microbiota synergy and their systemic impacts on host immunity are poorly defined.

Plant genotype shapes microbiome assembly largely through the composition and abundance of root exudates [11, 12]. Resistant cultivars often release elevated levels of phenylpropanoids and terpenoids [13, 14], compounds that directly suppress pathogens and simultaneously serve as chemical cues to recruit antagonistic microbial taxa (e.g. *Bacillus*, *Paenibacillus*, and *Penicillium*), thereby forming an initial biological defense barrier at the root interface [15, 16]. For example, upon *Fusarium* infection, resistant roots secrete metabolites such as the isoflavone formononetin and the hydroxy-benzoate isovanillic acid, which stimulate beneficial microbes, indirectly curbing pathogen proliferation [17–20]. Defense mechanisms, however, extend beyond the rhizosphere [21]. Leaf tissues emit volatile terpenes, deposit specialized cuticular waxes, and release distinct metabolites [22], actions that selectively filter local microbial colonization and transmit long-distance signals through vascular or airborne pathways to coordinate with root-based defense [23, 24]. Despite the phyllosphere’s harsh conditions, characterized by intense ultraviolet (UV) radiation and limited moisture, resistant genotypes successfully recruit and sustain diverse beneficial microbial communities [25]. This spatial extension suggests the plant–microbe defense network operates well beyond the rhizosphere, with the phyllosphere functioning as an auxiliary frontline that reinforces subterranean immunity once infection is sensed belowground [26]. Yet, most studies to date have focused on a single niche, typically the rhizosphere, leaving the cooperative roles of root endosphere and phyllosphere communities in systemic resistance underexplored [9, 27–29]. Furthermore, the temporal and spatial choreography linking metabolites, microbes, and host signaling remains poorly understood. Thus, dissecting how plant genotypes orchestrate beneficial microbiota across distinct niches (rhizosphere, root endosphere, and phyllosphere), alongside identifying key metabolites mediating this coordination, will provide a mechanistic framework for designing multi-niche microbial defenses.

Synthetic microbial communities (SynComs) are emerging as powerful tools for precision engineering in plants [30]. Two complementary SynCom assembly strategies are often used to guide community design [31]. A top–down approach involves reconstructing host-selected core taxa that are enriched in resistant, compared to susceptible, cultivars. For instance, rhizosphere-mediated recruitment of beneficial taxa in resistant wheat lines was previously characterized [32], where enriched microbes were reassembled into a SynCom to enhance plant stress resistance. Conversely, a bottom–up approach first screens for specific beneficial microbial traits, such as antifungal metabolite production or niche complementarity, subsequently combining isolates exhibiting these targeted functions [31, 33]. Each strategy offers distinct advantages depending on experimental goals and ecological contexts. Top–down consortia maintain ecological coherence and capture microbes that have co-evolved with the host, whereas bottom–up consortia provide targeted functional capability against the pathogen [34, 35]. Most current studies have employed only one of these strategies and focus on a single microbial niche, leaving unexplored the comparative efficacy and potential cross-niche synergies between these strategies. Moreover, the molecular mechanisms through which SynComs modulate host metabolism and immunity to achieve disease suppression remain poorly characterized.

Here, we utilized *Fusarium*-tolerant soybean cultivar (GXD2) and a susceptible counterpart (ND12) to systematically track the multi-niche dynamics of the plant holobiont during *F. falciforme* infection. By integrating metabolomics, microbiome profiling, and transcriptomics, we identified key metabolites responsible for microbial recruitment and developed cross-kingdom synthetic communities (SynComs) through top–down (host-selected core taxa) and bottom–up (highly antagonistic isolates) approaches. The constructed SynComs significantly reduced root rot disease severity and promoted soybean growth, revealing distinct underlying molecular mechanisms for each microbial consortium. Our results establish a “metabolite-microbiota coupled, multi-niche collaborative defense” model, advancing fundamental understanding of plant–microbiome interactions and providing a robust foundation for sustainable disease management in soybean and potentially other crop species.

## Materials and methods

### Experimental design and sampling

Surface sterilization of soybean seeds (tolerant variety GXD2 and susceptible variety ND12) was performed using 1% sodium hypochlorite solution for 5 min, with three subsequent rinses in sterile double-distilled water (ddH₂O). Uniformly sized, surface-sterilized seeds were planted in sterile vermiculite under controlled growth conditions (25°C/18°C day/night cycle, 16-h light/8-h dark photoperiod). At the two-true-leaf developmental stage, plants were inoculated with *F. falciforme*, involving mechanical root wounding followed by immersion in 100 ml of conidial suspension (1 × 10^8^ colony-forming units (CFU)/ml in sterile aqueous solution) for 6 h. The *F. falciforme* conidial suspension and root wounding inoculation were prepared as previously described [36, 37], with specific modifications detailed in the supplementary information. Control plants were mock-wounded and immersed in sterile water without conidia. Four treatments were established in a factorial design: uninoculated GXD2 (T), uninoculated ND12 (S), *F. falciforme*–inoculated GXD2 (T + F), and *F. falciforme*–inoculated ND12 (S + F). Each treatment comprised six pots (13.5 × 12 × 10.2 cm) filled with 1.5 kg of sterilized soil (Ultisols soils) and planted with four seedlings per pot under growth-chamber conditions. This controlled substrate system was used to reduce background variation from resident soil microbiota and to enable causal inference of pathogen- and genotype-driven microbiome assembly. All pots were arranged in a randomized complete block design and re-randomized weekly. Root-rot scoring and biomass measurements were performed by an investigator who was blind to treatment identity. At 10 days postinoculation (dpi), following disease assessment, root-rot severity in susceptible varieties was documented alongside plant and root biomass measurements. Microbial samples were collected from the rhizosphere, root endosphere, and leaf endosphere of each pot.

Rhizosphere soil was sampled by shaking off loosely adhering soil from uprooted plants, followed by placing the roots into sterile tubes containing 30 ml phosphate-buffered saline (PBS, pH 7.2) [28, 36]. Rhizosphere fractions were separated by vortexing vigorously for 2 min and centrifugation at 12 000 rpm for 10 min. Root endosphere and leaf endosphere compartments were sampled through surface sterilization protocols involving surface sterilization by 70% ethanol (1 min), 2% NaClO (2 min), and three rinses in sterile water [39]. All specimens designated for amplicon, metagenome, and metabolic sequencing were stored at −80°C, whereas samples reserved for microbial isolation were maintained at 4°C. The factorial design (2 genotypes × 2 treatments × 3 compartments × 6 replicates) yielded 72 sequencing samples, with 24 samples collected for each compartment (rhizosphere, root endosphere, and leaf endosphere).

### DNA extraction and amplicon sequencing

Microbial DNA from different ecological niches (rhizosphere, root endosphere, and leaf endosphere) was extracted using the Fast DNA SPIN Kit for Soil (MP Biomedicals, Santa Ana, CA) according to the manufacturer’s protocol [29]. DNA quantification was conducted using a NanoDrop 2000c UV-Vis micro-volume spectrophotometer (Thermo Scientific, Wilmington, USA), with subsequent normalization to uniform concentrations [28]. Bacterial and fungal community profiling was achieved through amplification of the 16S ribosomal RNA (rRNA) genes V3–V4 regions and internal transcribed spacer 1 (ITS1) region using universal primer sets 338F/806R and ITS1F/ITS2R, respectively [40]. The abundance of *F. falciforme* in the rhizosphere was determined via absolute quantification using *p*MD18-T (Takara, Japan) recombinant plasmids as external standards. A standard curve was established through a 10-fold serial dilution of the plasmid (10^1^–10^7^ copies/μl). Detailed quantitative real-time PCR (qPCR) protocols and primer sequences (Fal-qF/Fal-qR) are cataloged in the supplementary information. The PCR amplicons were sequenced on a MiSeq System (Illumina) with a PE300 strategy at Majorbio Bio-pharm Technology Co., Ltd (Shanghai, China). Raw sequence data are available at the National Center for Biotechnology Information (NCBI) sequence read archive (SRA) under the accession number PRJNA1277538.

Paired-end reads were quality-filtered with fastp v0.23.2 and merged with FLASH v1.2.11 [41, 42]. The raw FASTQ files were then processed using QIIME2 and DADA2 to remove primers and low-quality sequences (score < 20, length < 200 bp) [43, 44]. 16S rRNA gene and ITS1 reads were processed separately and analyzed with a Naïve Bayes classifier trained on amplicon sequence variants (ASVs) [45]. The SILVA database (v138) and UNITE database (v8.0) were used for taxonomic assignments of bacteria and fungi, respectively [46, 47]. ASV tables were rarefied to 6874 reads per bacterial sample and 2917 reads per fungal sample to standardize sequencing depth.

Alpha diversity indices such as Observed ASVs and the Shannon index were calculated using Mothur software [48]. One-way analysis of variance (ANOVA) was used to compare alpha-diversity indices among treatments. Factors affecting the Shannon index were assessed using type II ANOVA in linear mixed modeling (LMM) [49]. To characterize microbial beta diversity, principal coordinate analysis (PCoA) was performed based on Bray–Curtis dissimilarities, followed by permutational multivariate analysis of variance (PERMANOVA) (adonis2 function, 999 permutations) to quantify the relative contribution of factors driving community dissimilarity within the R vegan package [39]. Microbiome assembly mechanisms were deciphered through a null model (999 randomizations) calculating the beta Nearest Taxon Index (βNTI) [50]. Deterministic processes were defined at |βNTI| ≥ 2 thresholds, stochastic processes at |βNTI| < 2 [51, 52]. Ecological processes were categorized into five regimes using βNTI and Raup–Crick–Bray (RCBray) metrics: heterogeneous selection (βNTI < − 2), homogeneous selection (βNTI > + 2), dispersal limitation (|βNTI| < 2 and RCBray >0.95), homogenizing dispersal (|βNTI| < 2 and RCBray < − 0.95), and undominated (|βNTI| < 2 and |RCBray| < 0.95) [51, 52]. ASVs of bacteria and fungi were clustered at the phylum level according to the Ward.D2 method to analyze and show microbial differences at the phylum level. Differential ASVs between treatments were identified via DESeq2 [53] with significance FDR-adjusted *P* < .05. Comparative microbial profiling utilized generalized linear models in the edgeR package [54], visualized through volcano plots (*P* < .05 significance cutoff). Microbiome enrichment patterns were interrogated using Venn diagrams and Sankey flow diagrams mapping taxonomic hierarchies of significant ASVs (|log_2_FC| ≥ 1, *P* < .05) across treatments. Cross-domain bacterial–fungal interactomes were reconstructed through Spearman correlation networks (*ρ* > 0.7, *P* < .05) using the *hmisc* package [55], with topological visualization in Gephi (v0.9.7). All ASV tables were rarefied to the minimum sample depth as described above; Good’s coverage values exceeded 99% for every sample.

### Metagenomic sequencing and data analysis

Sixteen rhizosphere DNA samples, with four biological replicates each for uninoculated GXD2, uninoculated ND12, *F. falciforme–*inoculated GXD2, and *F. falciforme–*inoculated ND12, were processed for metagenomic sequencing on a NovaSeq 6000 System (Illumina) at Guangdong Magigene Biotechnology Co., Ltd (Guangzhou, China), with a PE150 strategy. Library construction employed the TruSeq DNA Sample Preparation Kit with dual-index barcoding, followed by quality verification via NanoDrop 2000 spectrophotometry and Qubit 2.0 fluorometry. Sequence preprocessing included adapter removal and quality filtering with Trimmomatic v0.39, and host reads were removed by aligning to the *G. max* reference genome (Bowtie2 v2.4.5). Taxonomic classification was performed with Kraken2 v2.1.2 against a custom RefSeq + GTDB database (build 2024-01-15), followed by abundance estimation with Bracken v2.8 [56–58]. Protein sequences were searched against UniRef90 using DIAMOND v2.1.8; Kyoto Encyclopedia of Genes and Genomes (KEGG) Orthology (KO), Carbohydrate-Active enzymes (CAZy), Clusters of Orthologous Groups (COG), and MetaCyc terms were assigned with egg NOG-mapper v2.1.9 [59].

Nonmetric multidimensional scaling (NMDS) based on Bray–Curtis dissimilarities was computed with the *vegan* package (v2.6.4) and visualized with *ggplot2* (v3.4.4) [60]. Shannon index of functional profiles was calculated with *vegan* and *picante* in R 4.4.1 [61]. Between-group differences in functional genes were calculated with one-way ANOVA and visualized by drawing a box plot. *The heatmap* package visualized the differences in the expression of key KEGG pathways between treatments [62]. Marker KOs for chemotaxis, motility, signal transduction, and core metabolic pathways were extracted, and their relative abundances were compared across treatments. We further compared the abundances of top contributing genera to these pathways among treatments. Raw reads have been deposited in the NCBI SRA under BioProject PRJNA1276253.

### Metabolite measurement

Exactly 100 mg (fresh weight) of each rhizosphere, root-endosphere, or leaf-endosphere sample was extracted with 1 ml of methanol:water (4:1, v/v) containing 10 μg/ml lidocaine as an internal standard. Samples were vortexed for 60 s, homogenized for 2 min, and sonicated for 10 min in an ice-water bath. The homogenization–sonication cycle was repeated three times, after which the extracts were incubated on ice for 30 min and centrifuged at 12 000 × g for 15 min at 4°C. Supernatants were analyzed on a Vanquish UHPLC System coupled to a Q Exactive Orbitrap Mass Spectrometer (both from Thermo Fisher Scientific) equipped with an ESI source operating in positive/negative switching mode [63]. The raw data files from mass spectrometry detection were imported into Compound Discoverer v3.1 with the following parameters: mass tolerance 5 ppm, signal-to-noise ≥10, minimum peak width 5 s, and total intensity ≥1 × 10^5^. Features were matched against mzVault, MassBank, HMDB4.0, and an in-house library; only matches with a score ≥ 80% and confirmed MS^2^ spectra (MSI Level 2) were retained. Twelve authentic standards provided Level 1 identification. Blank (solvent) and pooled quality control (QC) injections were used to subtract background ions and correct signal drift via locally estimated scatterplot smoothing (LOESS) normalization. Metabolites with a coefficient of variance (*CV*) >30% were removed from QC samples, and the remaining data were subjected to logarithmic transformation for further analysis, resulting in the final quantitative data of metabolites [64].

Orthogonal partial least squares discriminant analysis (OPLS-DA) was performed to examine metabolomic distinctions among plant compartments [27]. Differential metabolites were identified by VIP > 1.0 and Benjamini–Hochberg-adjusted *P* < .05 (two-tailed Student’s *t*-test). Volcano plots were generated for visual exploration of differential metabolites using the *ggplot2* package. Data were standardized using *z*-score normalization, followed by Euclidean distance matrix computation and complete-linkage hierarchical clustering implemented with the base R *dist* and *hclust* functions. The abundance patterns of selected differential metabolites across experimental groups were visualized using the *pheatmap* package (v1.0.8), with color gradients representing relative concentrations and dendrograms indicating cluster relationships.

### Isolation and identification of culturable microbes

Rhizosphere, root-endosphere, and leaf-endosphere samples from both soybean genotypes with *F. falciforme* inoculation were used to isolate culturable bacteria and fungi. Samples were stored at 4°C and processed for isolation at room temperature within 24 h. Rhizosphere subsamples (1 g) were suspended in 9 ml sterile water and shaken at 200 rpm for 20 min. Root endosphere and leaf endosphere tissues (1 g) were macerated in 9 ml sterile PBS (pH 7.2). Serial 10-fold dilutions (10^−1^–10^−6^) were prepared, and 100 μl aliquots were spread onto three different culture media, including nutrient agar (NA), potato dextrose agar (PDA), and modified Martin medium (Table S1, Supplementary Information). Distinct colonies were picked, inoculated into 2 ml lysogeny broth (LB) (for bacteria) or potato dextrose broth (PDB) (for fungi), and shaken at 28°C for 3 days. Colonies were then restreaked to obtain pure cultures. Total genomic DNA of the isolates was extracted using the TIANamp Bacteria DNA Kit (DP302) and Fungal Genomic DNA Kit (DP317) (TIANGEN Biotech, Beijing, China) according to the manufacturer’s protocols. The Sanger sequencing services provided by Sangon Biotechnology (Shanghai) Co., Ltd, were used to identify each isolate. The 16S rRNA genes (for bacteria) and ITS regions (for fungi) were amplified using the 27F/1492R and ITS4/ITS1 universal primers, respectively. Detailed primer sequences and cycling parameters are provided in Supplementary Table S2. The sequencing results were assembled and trimmed using the SeqMan software, and subsequently aligned against bacterial and fungal sequences separately, using the EzBiocloud website [9, 65] and the NCBI website, to determine the species information of isolated microorganisms. Phylogenetic trees were reconstructed using the neighbor-joining method in MEGA 11.0 based on multiple sequence alignments generated with ClustalW and visualized with iTOL v6 (https://itol.embl.de/) [66]. The unique strains were stored as 50% glycerol stocks at −80°C.

### Measuring *in vitro* antagonistic activities of microbiota against *F. falciforme*

The *F. falciforme* strain was isolated from diseased soybean root samples collected from the Ningxi base of South China Agricultural University in Guangdong province. A total of 476 initial isolates were recovered from three niches of tolerant and sensitive soybean. To avoid redundancy, these isolates were subjected to de-replication and identification based on NCBI Basic Local Alignment Search Tool (BLAST) analysis of 16S rRNA gene (for bacteria) and ITS (for fungi) sequences. Consequently, 89 unique representative strains (comprising 50 bacteria and 39 fungi) were selected for the subsequent antagonism assays. The antagonism test measured the *F. falciforme* radius and inhibition zone formed between the tested strain and *F. falciforme*. The smaller the radius of *F. falciforme*, the stronger the inhibitory ability of the test strain to *F. falciforme*. For screening the antagonistic strains, petri dishes containing PDA solid medium inoculated only with *F. falciforme* (1 cm diameter) in the center served as the negative controls. For the experimental groups, 5 μl of each culture (1 × 10^8^ CFU ml^−1^) were spotted to the four corners of the plates, 2.5 cm away from the center. All petri dishes were incubated at 28°C in the dark for 7 days, until the control plates were fully colonized by *F. falciforme*. The radial growth of *F. falciforme* in the control (R_1_) and experimental groups (R_2_) was then measured with a ruler. The inhibition rate of radial growth of *F. falciforme* by the isolated bacteria was calculated as follows: 100% × [(R_1_ − R_2_)/R_1_] [38].

### Construction of synthetic microbial communities (SynComs)

To investigate the functional role of microbiota in disease-tolerant cultivars in three plant compartments: rhizosphere, root endosphere, and leaf endosphere, we developed SynComs through complementary approaches: a host-selected top–down strategy that reconstructed ASV-enriched genera from the disease-tolerant cultivar [67] and a function-driven bottom–up strategy that selected isolates exhibiting antagonism toward *F. falciforme* [68]. These SynComs were systematically categorized as bacterial (Bac), fungal (Fun), cross-kingdom (Cross-K), or heat-killed Cross-K (HK-Cross-K) controls across each compartment. Bacteria were grown in LB to OD_600_ = 0.2; fungal isolates were grown on PDA, harvested as spore suspensions (1 × 10^6^ spores ml^−1^), and mixed at equal volumes to assemble Cross-K consortia. Each SynCom was first screened for *in vitro* inhibition of *F. falciforme* as described in the Measuring *In Vitro* Antagonistic Activities of Microbiota against  *F. falciforme* section.

Here, we evaluated the suppressive efficacy of SynComs against *F. falciforme* root rot using susceptible soybean cultivars. Vermicompost-grown seedlings received SynComs treatments 1 week after germination, with compartment-specific colonization achieved through targeted delivery: rhizosphere communities were established via soil drenching (10 ml/plant), root endosphere communities by root dipping for 6 h during transplanting, and leaf endosphere communities through foliar spraying (2 ml per plant, 50 μm droplet size). Both rhizosphere and leaf endosphere inoculations were performed every 3 days (six applications in total). Following a previously described method [69], the soil was fully covered with waterproof polyethylene (PE) film during spraying. To control for nonbiological effects, equivalent heat-killed SynComs and a sterile-water mock were applied. After a 7-day acclimation, roots were wounded and drenched with 100 ml of a *F. falciforme* spore suspension (1 × 10^6^ spores ml^−1^). All seedlings were subsequently transferred to sterile soil in a growth chamber (25°C/18°C day/night, 16/8-h photoperiod). Each treatment comprised six pots (biological replicates) containing four plants each. Pathogen-only and water-only pots served as negative controls. Root-rot severity was assessed at 14 dpi using a 0–4 ordinal disease severity scale, with scoring performed blind to treatment. The detailed scoring criteria are provided in Supplementary Table S3. In parallel, shoot height (cm), fresh biomass (g), root length (cm), and chlorophyll content (SPAD-502) were recorded.

### Characterization of SynComs stability and interaction dynamics

To evaluate the internal stability and interaction mechanisms of the SynComs, individual isolates, pairwise combinations, and their full consortia were standardized to a uniform OD_600_ (0.2) and inoculated (2 μl per 200 μl system) into 1/2 PDB medium buffered with 50 mM 4-(2-hydroxyethyl)-1-piperazineethanesulfonic acid (HEPES, pH 7.0). Growth kinetics were monitored by measuring OD_600_ regularly over a 60-h incubation period. To identify keystone contributors and interspecies interactions, “leave-one-out” (drop-out) assays were conducted alongside the pairwise growth profiling. Furthermore, *in planta* persistence was tracked using 16S rRNA gene and ITS1 amplicon sequencing to quantify niche occupancy under host-selective pressure.

### RNA extraction and RNA-Seq analysis

To evaluate how SynComs originating from the rhizosphere, root endosphere, and leaf endosphere modulate plant transcriptional responses to *F. falciforme*, we performed RNA-seq. After a 14-day incubation period, root tissue was harvested from plants inoculated with Cross-K SynComs and the corresponding heat-killed controls. Each treatment was represented by three biological replicates, yielding 36 root samples in total. Total RNA extraction from roots was performed using RNAiso Plus reagent (Takara Bio, Shiga, Japan) following the manufacturer’s protocols. Constructed libraries were subjected to quality assessment prior to sequencing on the Illumina platform, generating 150 bp paired-end reads. Raw sequence data are available at the NCBI SRA under the accession number PRJNA1276240.

Raw reads were processed to obtain clean reads through removal of adapter-contaminated sequences, poly-N stretches, and low-quality reads (Q-score < 20). Quality-filtered reads were aligned to the Wm82 soybean reference genome (a4_v1, SoyBase) using Hisat2 v2.0.5. Transcript quantification was performed with StringTie v2.2.1 [70], with gene expression levels calculated as FPKM (Fragments Per Kilobase of transcript per Million mapped reads). Differential expression analysis was conducted using DESeq2 v1.16.1 in R, applying Benjamini–Hochberg correction for multiple testing (adjusted *P*-value < .05). Functional enrichment analysis of differentially expressed genes (DEGs) was performed through Gene Ontology (GO) and KEGG pathway assessments using cluster Profiler v4.1.2 with gene length bias correction. Principal component analysis (PCA) of transcriptome profiles and multi-group volcano plot visualizations were implemented using *prcomp* and *ggplot2* packages in R v4.4.1, respectively [71, 72].

### Effects of selected metabolites on the growth of candidate beneficial microbes

Twenty metabolites that were significantly (FDR < 0.05) enriched in *F. falciforme*–infected rhizosphere, root endosphere, or leaf endosphere were selected for functional assays. We evaluated their microbial recruitment potential and growth-regulatory effects through systematic bioassays [10]. Isolates were grown overnight in nutrient broth (NB) at 28°C, 170 rpm. Cultures were washed twice in NB and adjusted to OD_600_ = 0.01. Aliquots (2 μl) of the diluted cultures were dispensed into 96-well plates containing 198 μl NB supplemented with each metabolite at a final concentration of 1 mM (triplicate wells). The plates were sealed with sealing film and incubated statically at 37°C within a SpectraMax M5 microplate reader (Molecular Devices, USA). Growth kinetics were monitored by measuring OD_600_ at 8.5-, 12.5-, 16.5-, 20.5-, and 22.5-h intervals. To ensure accurate and representative readings, the reader was programmed to perform a brief orbital shaking session prior to each measurement to fully resuspend the microbial cells. Fungal spores (5 μl, 1 × 10^6^ spores ml^−1^) were point-inoculated onto PDA amended with 1 mM metabolite or a control (plain PDA). After 72 h at 28°C, colony areas were quantified using ImageJ based on diameters measured in two perpendicular directions (*n* = 3). For statistical analysis, raw data (x) were normalized via log_2_(x + 1) transformation and analyzed by one-way ANOVA with Tukey’s *post hoc* test (*P* < .05).

### Statistical analysis

Data were statistically analyzed and presented graphically using the R 4.4.1 statistical environment (R Core Team, 2024; https://cran.r-project.org/), Prism 10.0 (GraphPad Software), and Origin 2022 (OriginLab Corporation).

## Results

### *F. falciforme* inoculation triggers severe root-rot symptoms and genotype-dependent colonization

**Figure 1:**
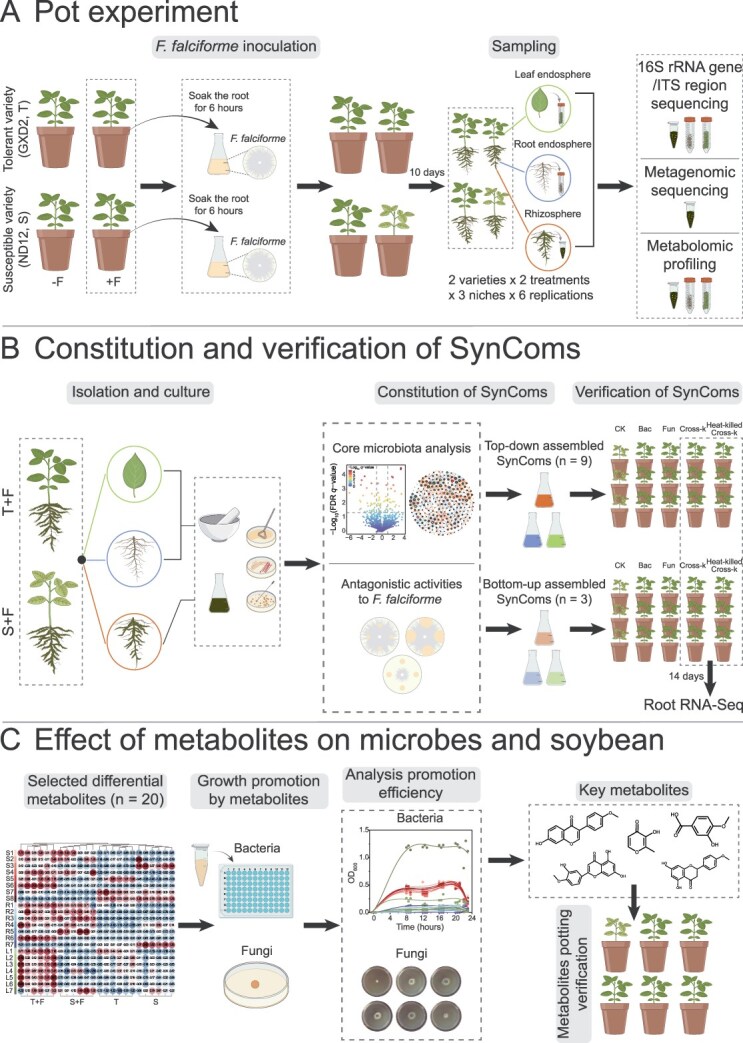
Schematic overview of the experimental and analytical workflow. (A) Pot experiment: Soybean plants of two genotypes (Tolerant, T; Susceptible, S) were subjected to either *F. falciforme* inoculation or mock treatment. Rhizosphere, root endosphere, and leaf endosphere samples (*n* = 6 per group) were collected 10 days postinoculation. Community composition was analyzed via 16S rRNA gene and ITS region amplicon sequencing. Metagenomics sequencing assessed rhizosphere microbial function, whereas metabolomic profiling and metabolite analysis elucidated soybean–microbe interactions. (B) Synthetic community (SynCom) construction and functional validation: microbial strains were isolated from the rhizosphere, root endosphere, and leaf endosphere of both healthy and diseased plants following *F. falciforme* inoculation. Twelve SynComs were assembled by integrating results from enriched microbial analyses (top–down strategy) and *F. falciforme* antagonism assays *in vitro* (bottom–up strategy). These SynComs were introduced into the susceptible genotype to identify the most effective consortium. Transcriptomic profiling (RNA-seq) was performed to identify differentially expressed genes. (C) Influence of metabolites on microbes and soybean: twenty metabolites selected from the rhizosphere, root endosphere, and leaf endosphere were tested for their ability to promote the growth of bacterial and fungal strains. This included analyzing metabolite–strain interactions, followed by evaluation of their disease-alleviating effects in a pot experiment.

**Figure 2:**
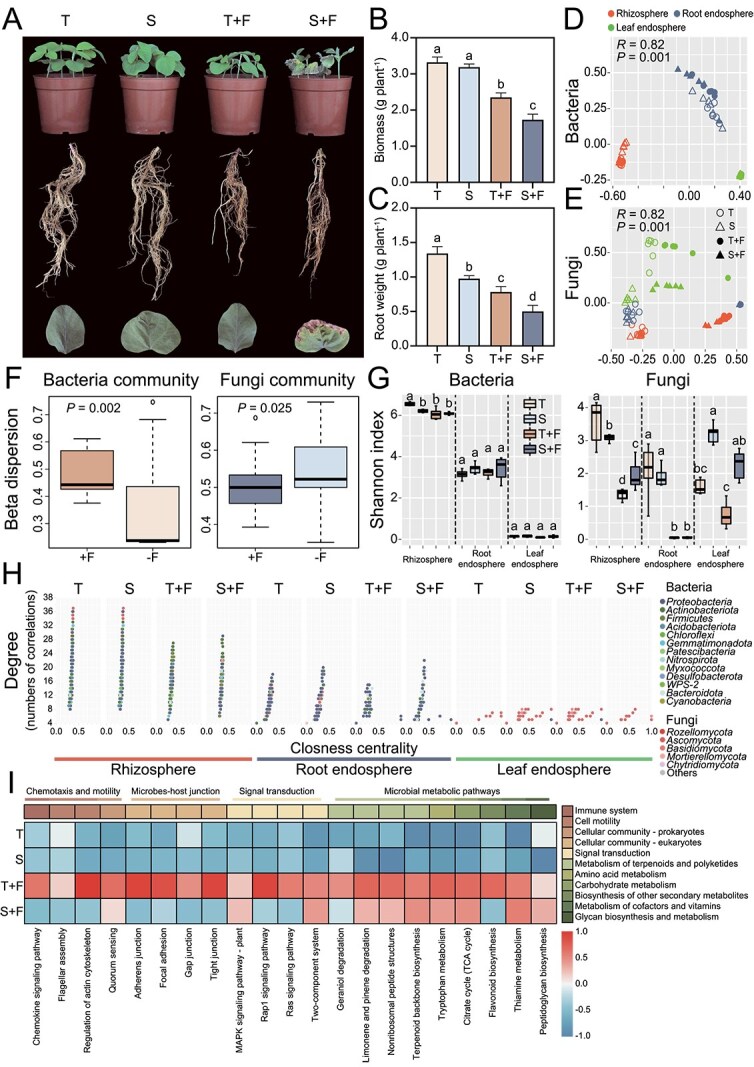
Phenotypic and multi-omic characterization of tolerant (T) and susceptible (S) soybean varieties under *F. falciforme* stress. (A) Representative phenotypes of shoots, roots, and leaves after inoculation. (B, C) Fresh weight of whole plant and roots (mean ± SD, *n* = 4). (D, E) Principal coordinates analysis (PCoA) of (D) bacterial and (E) fungal communities in rhizosphere, root endosphere, and leaf endosphere. (F) Beta-dispersion of microbial communities under inoculated (+F) and noninoculated (–F) conditions. (G) Shannon index of bacterial and fungal communities across three compartments. (H) Network topological characteristics correlating degree and closeness centrality. (I) Metagenomic functional heatmap of chemotaxis, signal transduction, and metabolic pathways across niches. The color scale represents standardized abundance levels, with the gradient indicating high (positive) to low (negative) relative enrichment. Distinct lowercase letters indicate statistically significant differences (*P* < .05, one-way ANOVA).

To investigate the response of soybean varieties with different genotypes to *F. falciforme* infection, a series of experiments combining multi-omics approaches and diverse validation methods was conducted (Fig. 1). In the pot assay, plants inoculated with *F. falciforme* exhibited a high disease incidence and developed classic root-rot symptoms. Shoots were stunted, roots turned brown and decayed, leaves became chlorotic or wilted, and some plants died (Fig. 2A). Correspondingly, the biomass of the tolerant variety (GXD2) decreased from 3.32 to 2.35 g following inoculation. In contrast, the susceptible variety (ND12) showed a more pronounced reduction, with its biomass dropping from 3.19 g in the control group to 1.73 g in the treatment group (ANOVA, *P* < .01, *n* = 4; Fig. 2B and C). To verify that symptom severity reflected pathogen load, we quantified *F. falciforme* copies in the rhizosphere at 0, 5, and 10 dpi. The standard and melting curves are provided in the supplementary information (Fig. S1). Although pathogen loads in both cultivars increased postinoculation, the tolerant cultivar (GXD2) exhibited a significant suppression after 5 dpi, maintaining a 5.53-fold lower abundance than the continuously surging susceptible one at 10 dpi (Fig. S2A). These findings indicate that *F. falciforme* infection severely restricts soybean growth and that the extent of root colonization, together with the resulting disease severity, is strongly modulated by host genotype.

### *F. falciforme* reshapes soybean microbiome in a compartment-specific manner

We characterized bacterial and fungal communities in rhizosphere, root endosphere, and leaf endosphere of two cultivars under inoculated and noninoculated conditions (Fig. 1A). Sequencing produced 4 340 452 high-quality 16S rRNA gene reads and 5 591 052 ITS1 reads across 72 samples, which were dereplicated into 6874 bacterial and 2917 fungal ASVs at exact-sequence resolution.

β nearest taxon index (βNTI) analysis revealed modest community restructuring after *F. falciforme* challenge (two-tailed Student’s *t*-test, *P* < .05). Inoculation shifted βNTI values for both bacterial and fungal assemblages in all compartments except the root endosphere microbiome (Fig. S2B and C). In the root endosphere, the susceptible cultivar exhibited a substantially higher proportion of homogeneous selection (βNTI < −2, 93.3%) than the tolerant cultivar (73.3%), indicating more rigid environmental filtering in susceptible plants post-infection (Fig. S2D and F). Across compartments, deterministic selection on fungi (βNTI < −2) increased in the rhizosphere and leaf endosphere, whereas inoculation reduced deterministic signals for fungi but enhanced them for bacteria inside roots (Fig. S2F and G). These findings indicate that bacterial microbiota of the tolerant root endosphere undergoes a more tightly deterministic re-assembly following *F. falciforme* infection, whereas the fungal component is comparatively buffered from host-genotype effects.

PERMANOVA confirmed that spatial niche was the principal driver of microbiome structure, that compartment accounted for 74.0% of the variation in bacterial composition and 26.0% in fungal composition (both *P* < .001; Table S4). In contrast, inoculation status and host genotype explained smaller, yet still significant, fractions of variance. Consistent with these statistics, Bray–Curtis PCoA cleanly separated bacterial communities by compartment along PC1 and partitioned fungal communities into below-ground versus aerial clusters (Fig. 2D and E, Fig. S3). Although bacterial composition at the phylum level showed distinct taxonomic shifts between compartments, fungal communities exhibited a more conserved phylum-level distribution, despite the statistically significant differences in their overall structure (Fig. S4). Beta-dispersion analysis indicated that bacterial assemblages became more heterogeneous after *F. falciforme* inoculation (Fig. 2F, Table S5). In the root endosphere, bacterial composition differed significantly between the tolerant and susceptible cultivars, in contrast to the fungal assemblages, which showed no significant variation (Table S6).

Linear-mixed-model analysis reinforced these trends. Whereas bacterial α-diversity was predominantly shaped by the compartment, the determinants of fungal α-diversity were metric-dependent. Specifically, *F. falciforme* inoculation was the primary driver of the fungal Shannon index, whereas the compartment exerted a stronger influence on Observed ASV richness (both *P* < .001; Table S7). Shannon index decreased from rhizosphere to root endosphere, and pathogen challenge reduced fungal diversity in the rhizosphere and root endosphere, whereas no significant reduction was observed in the leaf endosphere (Fig. 2G). Genotype effects were weaker but significant for the fungal Shannon index (*P* = .005) and bacterial Observed ASV richness (*P* = .017). Together, these results demonstrate that spatial niche imposes the primary ecological filter on the soybean microbiome, but pathogen invasion superimposes a genotype-dependent restructuring that is most evident in the bacterial community of the tolerant root endosphere.

### *F. falciforme* remodels cross-kingdom networks and selects compartment-specific beneficial taxa

Co-occurrence networks differed strongly among compartments and were reshaped by *F. falciforme* infection in a genotype-dependent manner (Fig. S5, Table S8). Across rhizosphere and root endosphere networks, infection generally reduced network size (fewer nodes/edges), whereas leaf endosphere networks remained sparse. Under infection (+F), the root endosphere network of the tolerant genotype was smaller and less connected than that of the susceptible genotype (Fig. 2H, Table S8), consistent with a more tightly host-filtered community that may contribute to resistance against *F. falciforme*. In the rhizosphere, the proportion of negative links decreased after inoculation in both genotypes but remained consistently higher in the susceptible genotype (ND12) than in the tolerant genotype (GXD2) (Table S8), suggesting a relatively more competitive association structure in ND12 under pathogen pressure. Hub analysis (top 1.0% betweenness centrality) identified *Bacillus*, *Streptomyces*, *Penicillium*, and an unclassified *Aspergillaceae* ASV as hubs in the rhizosphere network; *Massilia* as a hub in the root endosphere network; and *Cladosporium* as a hub in the leaf endosphere network (Table S9).

Differential-abundance analysis showed strong, compartment-specific recruitment after inoculation (Fig. S6). In the susceptible cultivar, 92, 27, and 0 bacterial ASVs, together with 23, 1, and 1 fungal ASVs, were enriched in the rhizosphere, root endosphere, and leaf endosphere, respectively. The tolerant cultivar responded with 81, 36, and 0 bacterial ASVs and only 1 fungal ASV in each niche, representing a restricted fungal shift. Summed across compartments, susceptible plants gained 119 bacterial and 25 fungal ASVs, whereas tolerant plants gained 117 bacterial but only 3 fungal ASVs (Table S10). Direct genotype contrasts highlighted 22 bacterial and 2 fungal ASVs enriched in the tolerant rhizosphere, 28 bacterial ASVs in the root endosphere, and 2 fungal ASVs in the leaf endosphere (Table S11). The most responsive taxa belonged to *Azospirillum*, *Bacillus*, *Paenibacillus*, *Brevibacillus*, *Massilia*, and *Penicillium* (Fig. S7A). Tolerant plants also accumulated ASVs of *Aspergillus*, *Penicillium*, and *Nigrospora* (Fig. S7B and C) and showed significant enrichment of *Pandoraea* in the rhizosphere and *Herbaspirillum* in the root endosphere (Fig. S7D–F). Collectively, these recruitment patterns indicate that the tolerant soybean cultivar preferentially recruits candidate beneficial bacterial taxa but largely excludes additional fungal responders.

### *F. falciforme* reshapes rhizosphere functional potential in a genotype-specific manner

Metagenomic profiling uncovered pronounced, genotype-dependent functional shifts in the rhizosphere microbiome following *F. falciforme* challenge. After quality control, the metagenome comprised 4987 bacterial and 442 fungal taxa with annotated genes. NMDS of KO, COG, CAZy, and MetaCyc profiles showed a clear separation between inoculated and noninoculated samples along the MDS1 axis (PERMANOVA, *R* > 0.67 in most cases, *P* = .001; Fig. S8A), whereas plant genotypes further differentiated samples along the MDS2 axis. Differential-abundance analysis revealed that inoculation significantly reduced the number of KO, COG, and MetaCyc features in both genotypes, whereas the size of the CAZyome did not change appreciably (Fig. S8B).

Despite this overall contraction, several functional categories remained over-represented in the tolerant rhizosphere. Genes linked to chemotaxis, flagellar assembly, signal transduction, and specialized metabolism were significantly more abundant in tolerant than in susceptible plants (Fig. 2I). Notable examples include the flagellar motor switch gene *fliG* (K02410), the tryptophan-metabolizing monooxygenase gene (K03392), and the geranylgeranyl-transferase gene involved in flavonoid degradation (K01692), which were 29.2%–59.4% more abundant in the tolerant microbiome than in the susceptible one following pathogen inoculation (one-way ANOVA, *P* < .05; Fig. S8C–E). Functional enrichment of secondary-metabolite biosynthesis pathways was largely attributable to the higher relative abundance of *Streptomyces*, *Pseudomonas*, and *Bacillus* in the tolerant rhizosphere (Fig. S6F). These results indicate that, although *F. falciforme* broadly contracts the pool of enriched functions, the tolerant genotype retains, and even amplifies, key traits associated with motility, signaling, and antimicrobial metabolism.

### SynCom promoted growth of susceptible soybean and suppress root-rot symptoms

**Figure 3:**
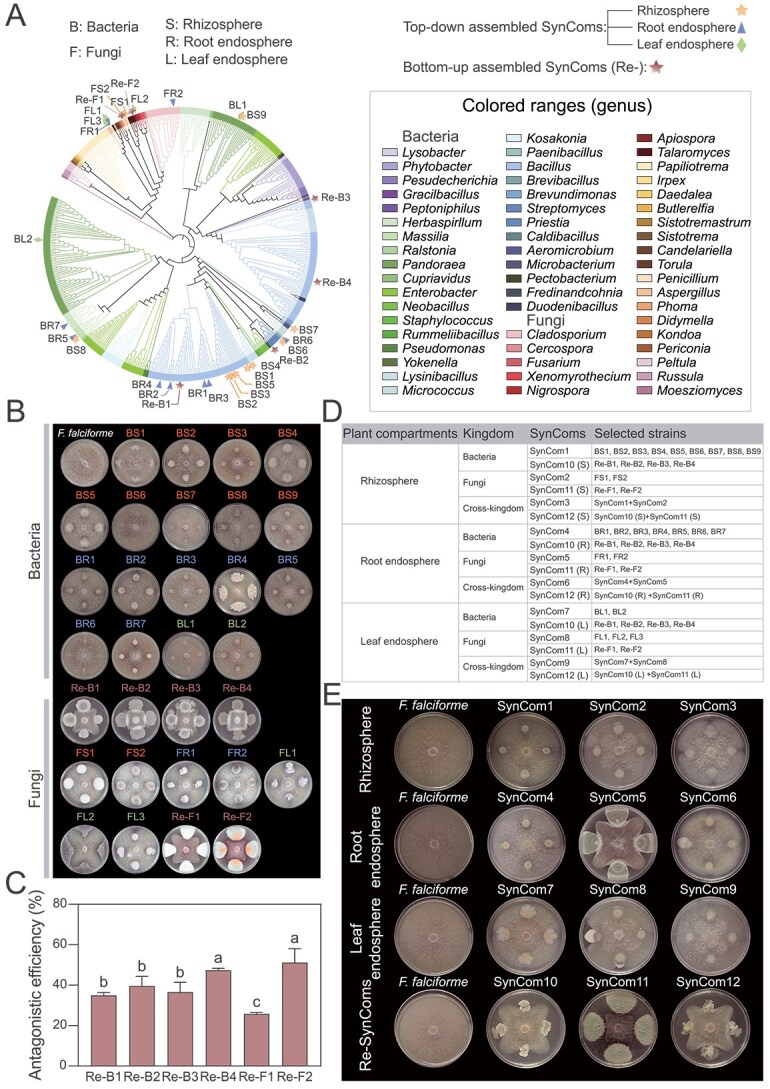
Construction of synthetic communities (SynComs) and their ability to suppress *F. falciforme*. (A) Maximum-likelihood phylogeny of the culturable bacteria and fungi isolated from the rhizosphere, root endosphere, and leaf endosphere; colored branches indicate taxa selected for SynCom assembly. (B) Dual-culture plates illustrating growth inhibition of *F. falciforme* by representative bacterial and fungal isolates from each niche. (C) Inhibition percentages for the four most active bacterial strains and the two most active fungal strains. Bars show mean ± SD (*n* = 3); bars with different lowercase letters differ significantly (one-way ANOVA, *P* < .05). (D) Design of 12 cross-kingdom SynComs. SynComs 1–9 were niche-specific; SynComs 10–12 were applied to all compartments via soil drench (rhizosphere, S), root dipping (root endosphere, R), or foliar spray (leaf endosphere, L). (E) Radial-growth assays comparing the suppressive efficacy of the 12 SynComs against *F. falciforme*.

Gradient-dilution plating yielded 476 culturable isolates from the rhizosphere, root endosphere, and leaf endosphere of tolerant and susceptible soybeans (Fig. 3A, NCBI BioProject PRJNA1433828). After dereplication, Sanger sequencing identified 50 unique bacterial species and 39 unique fungal species. Dual-culture assays classified 10 isolates as contact-dependent inhibitors, 9 as producers of diffusible antibiotics, and 44 as nonantagonistic (Fig. 3B, Fig. S9). Antagonists were dominated by *Bacillus*, *Caldibacillus*, *Talaromyces*, *Cladosporium*, and *Penicillium* (Fig. 3C, Table S10).

**Figure 4:**
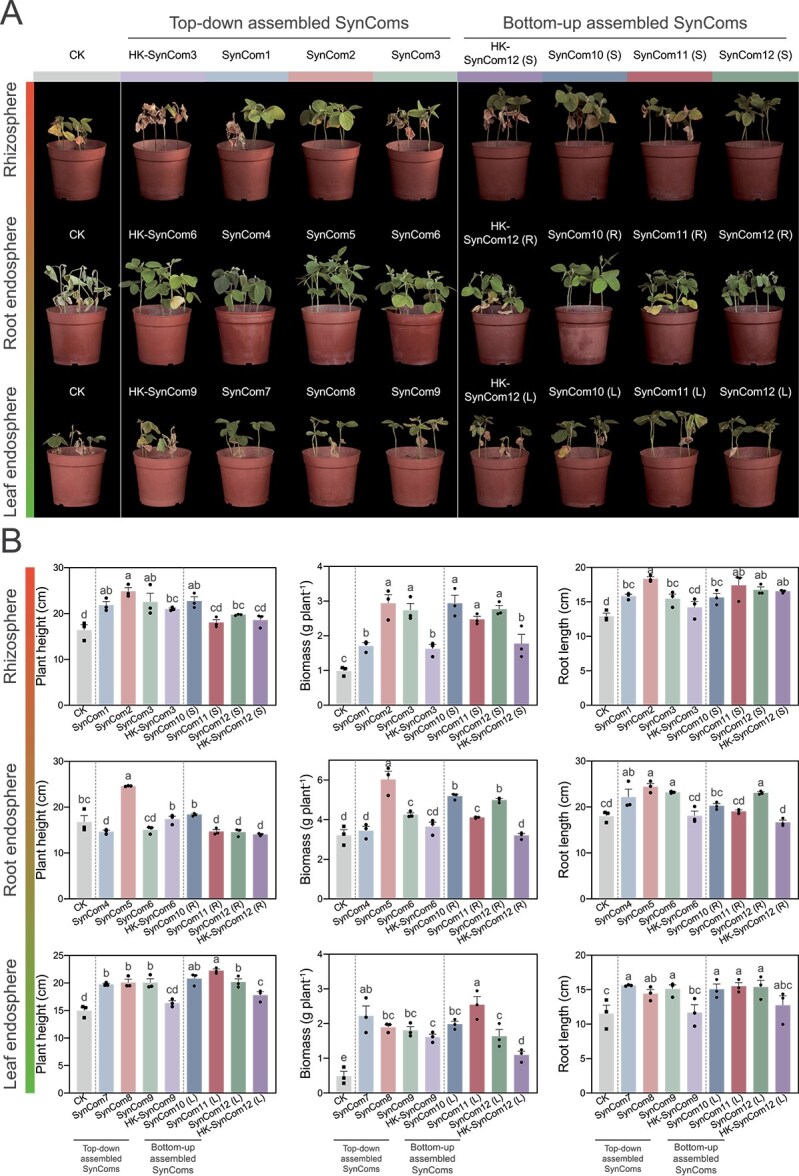
SynCom-mediated mitigation of *F. falciforme* root rot in susceptible soybean. (A) Representative ND12 plants 14 days after inoculation with the indicated SynComs. (B) Plant height, shoot biomass, and root length. Bars show mean ± SD (*n* = 3). Columns sharing the same lowercase letter do not differ significantly (one-way ANOVA, *P* < .05).

Twelve SynComs were assembled according to two design rules. The first involved taxa selectively enriched in the tolerant cultivar (top–down), and the second included strains exhibiting strong antagonism toward *F. falciforme* (bottom–up), plus their combinations (Fig. 3D and E, Table S12). The stability of the designed SynComs was validated through growth kinetics and drop-out assays (Figs S10–S14). Complete SynComs exhibited a significant advantage in saturation density OD_600_ after 48 h compared to individual isolates (*P* < .05; Fig. S10E–H), suggesting niche complementarity. Drop-out assays identified *Bacillus* sp. (BR1) as a keystone structural donor; its removal led to a marked reduction in community biomass (Fig. S12G). In susceptible cultivar ND12, every live SynCom reduced disease relative to the water control (CK) (Fig. 4A). SynComs reduced disease relative to the water control (CK) (Fig. 4A). Overall, cross-kingdom (Cross-K) consortia provided robust and consistent disease suppression across assays (Fig. 4B, Fig. S15). However, fungal SynComs achieved comparable or even greater growth promotion in specific compartments, particularly when the root endosphere or the leaf endosphere was targeted (Fig. 4B). SynComs built from tolerant-enriched taxa outperformed antagonist-only SynComs in the root and leaf endosphere niches, as indicated by a lower disease index (ANOVA; Fig. S15B and C). Unexpectedly, heat-killed root endosphere SynComs (HK-Cross-K) still conferred partial protection, suggesting a metabolite-mediated effect. In planta recolonization assays revealed the selective nature of community persistence. Target fungi and *Massilia* sp. (BR5) successfully occupied dominant niches (Figs S16 and S17), whereas *Herbaspirillum* sp. (BR7) failed to persist. These data indicate that the SynCom establishes a defensive barrier through a combination of keystone-driven biomass stability and host-selected niche occupancy.

**Figure 5:**
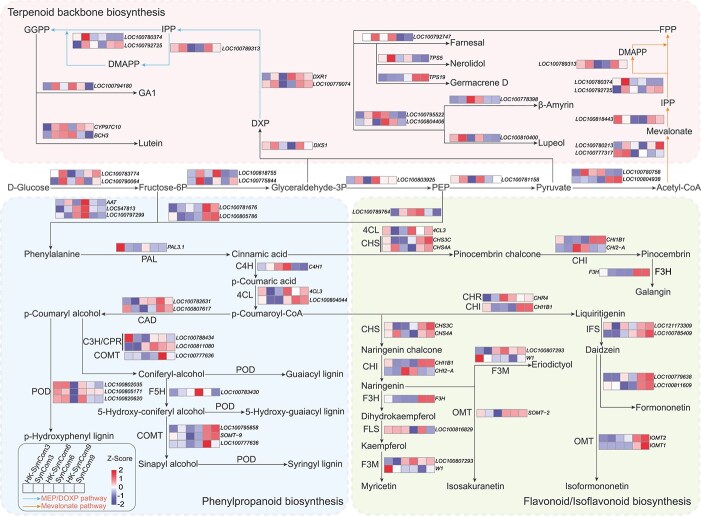
Map of differential expression in the terpenoid-backbone, phenylpropanoid, and flavonoid/isoflavonoid biosynthetic pathways. The heat map shows transcript levels in live cross-kingdom SynComs and their heat-killed counterparts constructed from microbes enriched in the rhizosphere (SynCom3 vs. HK-SynCom3), root endosphere (SynCom6 vs. HK-SynCom6), and leaf endosphere (SynCom9 vs. HK-SynCom9). The Z-score color scale represents standardized transcript levels relative to the corresponding heat-killed controls, with positive values indicating up-regulation and negative values indicating down-regulation.

**Figure 6:**
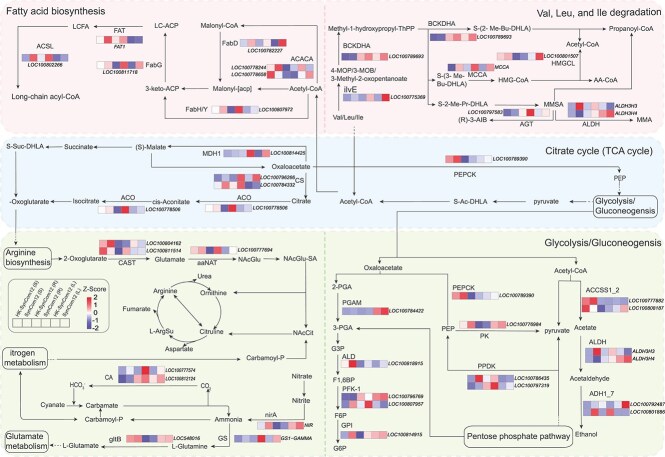
Differential expression of core primary-metabolism pathways after inoculation with antagonist-based SynComs. The heat map displays transcript levels for key enzymes in the tricarboxylic-acid (TCA) cycle, glycolysis/gluconeogenesis, fatty-acid biosynthesis, and nitrogen-metabolism pathways. Columns compare each live cross-kingdom SynCom built from *Fusarium*-antagonistic isolates with its heat-killed control. The heat-map displays transcript levels in live cross-kingdom SynComs and their heat-killed counterparts assembled from *F. falciforme–*antagonistic isolates in the rhizosphere [SynCom12 (S) vs. HK-SynCom12 (S)], root endosphere [SynCom12 (R) vs. HK-SynCom12 (R)], and leaf endosphere [SynCom12 (L) vs. HK-SynCom12 (L)]. The Z-score color scale indicates standardized transcript levels relative to the corresponding heat-killed controls, where positive and negative values represent higher and lower relative expression, respectively; blank (white) cells denote genes not detected in that comparison.

The same six cross-kingdom SynComs used for transcriptome analysis were evaluated for plant protection efficacy, specifically involving three tolerant-enriched consortia (SynComs 3, 6, and 9) and three antagonist-based consortia (all based on SynCom 12) (Fig. 4A and B, Table S13). PCA separated SynCom-treated and control samples along PC1 (72.3% variance) in all compartments (Fig. S9A and B). Stringent filtering (|log_2_FC| ≥ 1, FDR < 0.05) identified 605, 435, and 243 up-regulated DEGs in roots, following inoculation with the top–down SynComs assembled from rhizosphere-, root endosphere–, and leaf endosphere–derived taxa, respectively (Fig. S18A and B). Bottom–up SynComs induced larger shifts (2377, 4249, and 527 up-regulated DEGs), indicating strategy-dependent activation of immunity (Fig. S18C). KEGG enrichment analysis revealed dual layers of defense. The first involved induced systemic resistance (ISR) signaling, and the second included niche-specific secondary-metabolite routes. Top–down Cross-K SynComs up-regulated terpenoid backbone biosynthesis (ko00900) in the rhizosphere, phenylpropanoid biosynthesis (ko00940) in the root endosphere, and flavonoid/isoflavonoid biosynthesis (ko00941) in the leaf endosphere (Fig. 5, Fig. S18D). By contrast, antagonist-driven SynComs chiefly remodeled primary metabolism, elevating nitrogen metabolism (ko00910), biotin metabolism (ko00780), and glycolysis/gluconeogenesis (ko00010) (Fig. 6, Fig. S18D).

Together, the physiological and transcriptomic data demonstrate that rationally designed SynComs can rescue a susceptible soybean cultivar from *F. falciforme* root rot, with cross-kingdom consortia providing the strongest protection by simultaneously stimulating ISR, fortifying specialized-metabolite pathways, and rewiring central metabolism.

### *F. falciforme* reshapes compartmental metabolomes and steers microbe–metabolite interactions

**Figure 7:**
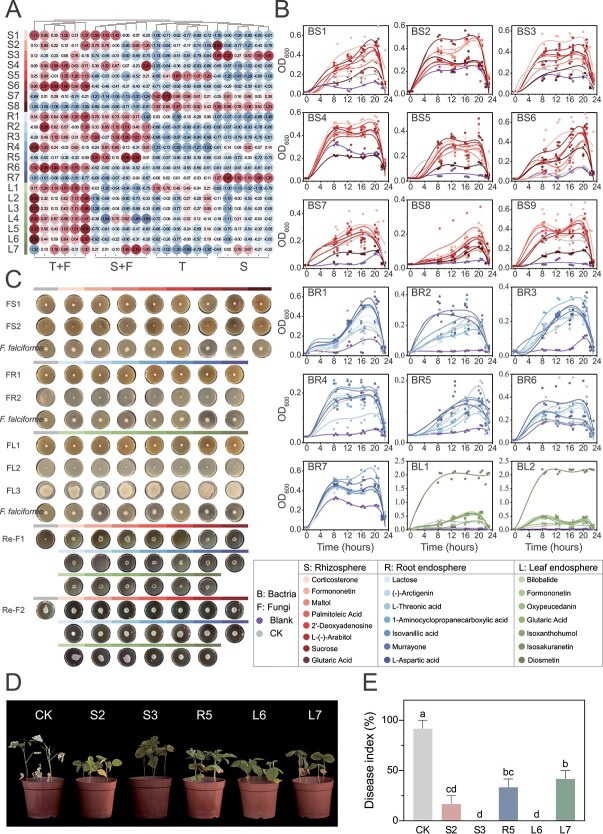
Target metabolites promote beneficial microbes and curb *F. falciforme* root rot. (A) Bubble plots showing compartment-specific enrichment of key metabolites in tolerant (GXD2) and susceptible (ND12) soybeans with (+F) or without (–F) inoculation. Bubble size corresponds to fold change; color denotes significance (Student’s *t*-test, *P* < .05). (B) Optical-density assays (OD^6^₀₀, 24 h) illustrating how the selected metabolites influence growth of SynCom bacterial members from each compartment. Values are expressed as percentage of solvent control. (C) Corresponding effects on SynCom fungi and on *F. falciforme* itself. (D) Representative plants from pot trials in which five metabolites were applied individually (soil drench or foliar spray, according to native compartment). (E) Disease-index reduction in the metabolite treatments. Bars show mean ± SD (*n* = 6); bars with different lowercase letters differ significantly (one-way ANOVA, *P* < .05).

Untargeted LC–MS profiling revealed pronounced, niche-specific remodulating of exudate chemistry after pathogen challenge. OPLS-DA models showed clear separation between inoculated and control samples in the rhizosphere (*R*^2^X = 0.68, *Q*^2^ = 0.95), root endosphere (*R*^2^X = 0.75, *Q*^2^ = 0.95), and leaf endosphere (*R*^2^X = 0.57, *Q*^2^ = 0.97) of both cultivars (permutation test, *P* < .01; Fig. S19A). Volcano plots and Venn diagrams identified differentially abundant metabolites (|log_2_FC| ≥ 1, *P* < .05) in each compartment (Fig. S19B). Specifically, in the rhizosphere, tolerant plants accumulated formononetin (log_2_FC = 1.10, *P* < .05) and maltol (log_2_FC = 1.25, *P* < .01), whereas both genotypes shared increases in corticosterone, palmitoleic acid, and L-arabitol (Fig. 7A). In the root endosphere, among 241 enriched metabolites, phenylpropanoid intermediates, that arctigenin, isovanillic acid, and murrayone, were strongly up-regulated in the tolerant cultivar (Fig. 7A). In the leaf endosphere, secondary metabolites rose broadly, with tolerant plants showing specific enrichment of flavonoids and terpenoids such as bilobalide, oxypeucedanin, isoxanthohumol, isosakuranetin, and diosmetin (Fig. 7A).

Twenty indicator compounds (top seven or eight from each compartment including two shared metabolites) were tested against *F. falciforme* and representative SynCom isolates (Fig. 7B and C, Fig. S20A). Most compounds stimulated beneficial bacteria rather than directly inhibiting the pathogen (Fig. 7C). Growth promotion was highly substrate-specific. For example, *Massilia* exploited formononetin (OD_600_ increase of 3.2%–14.9%) and palmitoleic acid (OD_600_ increase of 25.9%–90.4%) but was repressed by other metabolites (Fig. 7B). Metabolites enriched in the tolerant genotype not only promoted the growth of bacteria such as *Bacillus*, *Streptomyces*, *Paenibacillus*, and *Herbaspirillum*, but also exerted significant positive effects on fungal endophytes (Fig. 7B and C). Quantitative analysis using ImageJ revealed that these resistance-linked metabolites significantly increased the colony areas of *Penicillium* and *Aspergillus*, whereas their effect on *Nigrospora* was marginal (Fig. S21). These results suggest that resistance-linked metabolites reshape host microbial interaction networks by selectively fostering the proliferation of both beneficial bacterial and fungal taxa.

To determine whether the metabolites that stimulated beneficial microbes *in vitro* could confer disease resistance in planta, we selected five key metabolites (formononetin, maltol, isovanillic acid, isosakuranetin, and diosmetin) to investigate their regulatory effects on soybean root rot. Pot experiments showed that exogenous addition of these metabolites increased soybean root length by 14.2%–41.5%, chlorophyll content (SPAD value) by 2.86–3.36 times, and biomass accumulation by 43.6%–206.3% (Fig. S20B and E). The disease index of root rot in the metabolite-supplemented treatment groups was significantly lower than that in the control group (*P* < .01; Fig. 7D and E). These results systematically reveal the ecological adaptation mechanism by which plants precisely regulate microbial communities through metabolome remodulation, thereby forming a “metabolic barrier” to resist soil-borne diseases.

## Discussion

By integrating microbiome, metagenomic, metabolomic, and transcriptomic data from tolerant and susceptible soybean cultivars, we show that whole-plant resistance to *F. falciforme* is achieved through niche-specific microbiota remodelling across the rhizosphere, root endosphere, and leaf endosphere. In the tolerant cultivar, core beneficial taxa, including *Bacillus*, *Streptomyces*, *Massilia*, and *Penicillium*, become consistently enriched across these compartments and assemble dense cross-kingdom interaction networks, associated with reduced pathogen burden and a marked suppression of root rot symptoms (Figs 2 and 4).

This study expands the classical “cry-for-help” paradigm by establishing a bidirectional, cross-compartment defense model initiated by root infection. Upon *F. falciforme* invasion in the root zone, the tolerant soybean cultivar triggers long-distance signaling, likely via hormonal or metabolite cues, that reprograms leaf metabolic profiles and promotes the selective recruitment of beneficial microbes in the leaf endosphere. These foliar-enriched taxa, including *Penicillium* and *Nigrospora*, are not merely passive responders but actively contribute to root protection, either through systemic signaling or direct downward migration. This dynamic feedback loop, from root to shoot and back to root, fortifies the host against *Fusarium*-induced root rot and supports a holistic view of plant immunity as an integrated, whole-plant microbiome-mediated network. The model is consistent with the previously proposed shoot–root signal-coordination hypothesis [73, 74] and extends it by providing direct experimental evidence of shoot-mediated microbial recruitment, reinforcing belowground defense. The multi-niche synergistic defense model proposed in this study is not only applicable to the soybean pathogens investigated here but also serves as a significant reference for other pathosystems characterized by vascular colonization or soil-borne transmission, such as *Ralstonia solanacearum* and *Verticillium* [75, 76]. This multi-compartmental synergy implies that defense mechanisms are not confined to the initial infection sites but extend across the critical dispersal pathways of the pathogen, thereby constructing a continuous biological barrier from localized tissues to systemic levels.

Plant-derived secondary metabolites serve as pivotal mediators linking host immunity with microbiome assembly [16, 77]. In this study, *F. falciforme* infection distinct metabolic shifts across the rhizosphere, root endosphere, and leaf endosphere, with tolerant soybean cultivars showing selective accumulation of phenolics and terpenoids, including formononetin, maltol, and isovanillic acid, which are previously implicated in microbiota modulation (Fig. 7A, Fig. S19). Although these metabolites lacked direct antifungal activity, they preferentially promoted the growth of beneficial taxa such as *Bacillus*, *Streptomyces*, and *Paenibacillus* (Fig. 7B and C), suggesting metabolite-mediated, indirect suppression of disease. The substrate specificity of individual microbes, for instance, *Massilia*’s preference for formononetin and palmitoleic acid, highlights a chemically precise recruitment mechanism. Exogenous application of these metabolites significantly enhanced root growth, chlorophyll content, and disease resistance in pot assays (Fig. 7D and E, Fig. S20). These findings support a model in which tolerant plants reconfigure their metabolome to construct a “metabolic shield” that fosters protective microbial consortia. Such metabolite-driven modulation of the microbiome is likely representative of a conserved evolutionary stratagem within the plant kingdom, rather than an isolated phenomenon. This is evidenced by the glutamate-mediated recruitment of *Pseudomonas* in *Arabidopsis thaliana* and the benzoxazinoid-dependent tuning of the maize rhizosphere [34, 78]. Although the chemical structures of the molecules secreted by different crops vary, the underlying logic of achieving “niche customization” through metabolic regulation remains highly consistent. Although the homologous receptors and downstream host signal transduction mechanisms remain to be fully elucidated, this study provides a mechanistic foundation for leveraging host metabolites to construct suppressive microbiomes.

Further greenhouse assays confirmed that cross-kingdom SynComs, constructed using either a “top–down” strategy reconstituting host-selected core taxa or a “bottom–up” strategy combining antagonistic isolates, consistently suppressed *Fusarium* root rot in the susceptible cultivar. Foliar application of SynComs retained strong protective effects, supporting the concept of “leaf endosphere-mediated rhizosphere protection” and highlighting its translational potential. Multi-omic profiling revealed that the two designs exploit different layers of host biology. Top–down Cross-K consortia triggered a classic immune-priming program. MAPK cascades (ko04016) and specialized-metabolite pathways, including terpenoid backbone (ko00900), phenylpropanoid (ko00940), and flavonoid biosynthesis (ko00941), were up-regulated 2- to 5-fold relative to heat-killed controls. Because these live communities showed no direct antibiosis against *F. falciforme in vitro*, their protection must arise from tripartite interactions among pathogen, plant, and microbiota. This is consistent with the idea that immune signals act first as “initiators,” inducing antimicrobial proteins and defensive metabolites [79]. Similar microbe-associated molecular pattern (MAMP)-driven cascades are well documented. Fungal chitin and bacterial flg22 evoke reactive oxygen species (ROS) bursts and MAPK activation in rice, increasing phenylpropanoids, flavonoids, and linoleic-acid derivatives [80]. The endophyte *Penicillium oxalicum* up-regulates *FLS2* and CaM-like genes in cucumber, boosting flavonoid and diterpenoid biosynthesis [81].

Unlike the immune-priming mechanism triggered by top–down consortia, bottom–up SynComs predominantly modulated primary metabolism. Across all compartments, they enriched for genes involved in the tricarboxylic acid cycle, glycolysis, fatty acid synthesis, and nitrogen metabolism, suggesting a metabolic reprogramming that rebalances carbon and nitrogen fluxes. This shift likely fuels microbial colonization and indirectly reinforces host defenses by promoting antioxidant accumulation and redox-hormonal cross-talk [82, 83]. These divergent mechanisms highlight the complementary potential of top–down and bottom–up SynCom strategies for integrated microbiome-based disease control.

The SynComs also induced compartment-specific secondary-metabolite signatures, with terpenoids dominating in the rhizosphere, phenylpropanoids in the root endosphere, and flavonoids in the leaf endosphere. Such spatial stratification suggests that plants allocate costly defences where they are most effective. Structural lignin precursors are below ground, and diffusible phytoalexins are above ground, thus balancing metabolic expenditure across niches. Removing the fungal fraction from either SynCom reduced protection (Fig. S15C and D). This protective efficacy stems from the synergistic inhibition of *F. falciforme* mycelial growth by polyketides secreted from *Penicillium* and *Cladosporium* isolates in conjunction with *Bacillus* [84–86]. These observations indicate that fungal partners not only contribute direct antagonism but also potentiate bacteria-triggered immune priming.

Although our findings provide a comprehensive model for microbiome-mediated disease resistance, certain limitations exist. Research remains confined to controlled experiments under greenhouse conditions. Transcriptomic profiling was performed exclusively on cross-kingdom SynCom-treated plants, as this configuration best mimics the multi-kingdom complexity of the native soybean holobiont. The resulting data therefore capture the composite host response to both bacterial and fungal MAMPs/effector repertoires, offering a holistic view of the immune and metabolic circuits engaged during successful biocontrol. Sterile substrates ensure reproducibility; the model and SynCom performance—including colonization persistence and ecological safety—must be further validated under diverse field conditions to account for complex natural microbial backgrounds. Furthermore, the specific molecular mediators of shoot-root signaling, such as hormones, small RNAs, or volatiles, and their spatiotemporal dynamics urgently require elucidation using single-cell and real-time tracing technologies. Finally, the genetic basis underlying the contrasting metabolite–microbe interactions of resistant versus susceptible cultivars remains unexplored. A logical next step is to couple high-resolution quantitative trait locus (QTL) mapping with metabolite genome-wide association studies and microbiome genome-wide association study (GWAS), thereby pinpointing the loci that link soybean exudate chemistry, microbiome assembly, and root-rot resistance; such information would underpin molecular-marker-assisted breeding of disease-resilient cultivars.

**Figure 8:**
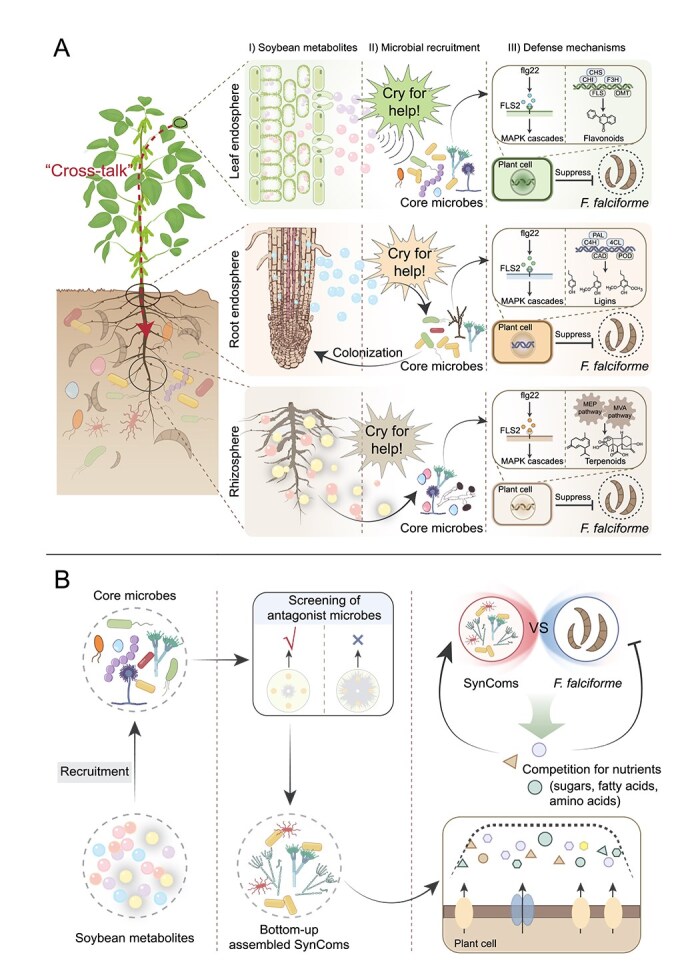
Contrasting modes of action of top-down and bottom-up cross-kingdom SynComs against *F. falciforme* root rot. The diagram integrates soybean metabolites, plant signaling pathways, and recruited microbes to illustrate how each SynCom design enhances defense. (A) In the top–down SynCom scenario, pathogen attack provokes each soybean compartment to secrete a distinct metabolite cocktail that doubles as a “cry-for-help” signal. In the rhizosphere, the isoflavone formononetin, together with maltol, selectively enriches *Bacillus*, *Brevibacillus*, and *Streptomyces*; these bacteria, in turn, activate MAPK cascades and terpenoid-backbone biosynthesis, curbing *F. falciforme* proliferation. In the root endosphere, exuded isovanillic acid draws in *Bacillus* and *Massilia*, whose presence accelerates lignin deposition, reinforcing cell walls and blocking pathogen entry. Concurrently, the leaf endosphere releases the flavonoids, diosmetin, and isosakuranetin, which attract *Pandoraea* and *Aspergillus*; this alliance triggers MAPK signaling and a surge in flavonoid production that further suppresses the pathogen, completing a three-tier, metabolite-guided defense network. (B) In the bottom–up SynCom scenario, communities built from plate-screened *F. falciforme* antagonists are applied to all three soybean compartments; once established, these bacteria–fungus consortia simultaneously secrete broad-spectrum antibiotics and remodulate host primary metabolism. Across the rhizosphere, root endosphere, and leaf endosphere, they up-regulate genes for the tricarboxylic-acid cycle, glycolysis, fatty-acid synthesis, and nitrogen assimilation, accelerating carbon- and nitrogen turnover within plant tissues. The heightened metabolic flux deprives *F. falciforme* of essential resources, whereas antimicrobial metabolites (e.g. iturins, fengycins, gliotoxin, and β-glucanases) released by the SynCom members further damage hyphae. Faced with the dual pressures of nutrient starvation and direct chemical assault, the pathogen’s growth inhibited, resulting in a lower disease index in the host.

In summary, we propose and substantiate a model of “metabolite–microbiota coupled, multi-niche collaborative defense.” Tolerant soybean cultivars precisely recruit cross-kingdom beneficial microbiota via specific metabolites, constructing a whole-plant immune barrier under bidirectional shoot–root signal regulation. SynComs assembled through both top–down (host-selected core taxa) and bottom–up (antagonistic strain selection) strategies significantly alleviated disease symptoms, albeit operating via distinct molecular and functional mechanisms (Fig. 8). Moreover, our data highlight an often-overlooked contribution of fungal partners, incorporating *Penicillium*, *Nigrospora,* and related isolates into the SynComs boosted *in planta* protection and reproduced native cross-kingdom complexity more faithfully than bacteria-only formulations. Collectively, these findings advance our understanding of plant–microbiota integrated defense strategies against root rot, provide a robust framework for the rational design of cross-kingdom SynComs, and offer valuable insights for developing sustainable and precise microbiota-based solutions for crop protection and breeding.

## Supplementary Material

Supplementary_Information_wrag080

## Data Availability

The amplicon raw sequencing data have been submitted to the National Center for Biotechnology Information (NCBI) Sequence Read Archive under accession numbers PRJNA1277538. The metagenomic raw sequencing data are available in the NCBI database under accession number PRJNA1276253. RNA-Seq data for all samples used in this study have been deposited in the NCBI database under project number PRJNA1276240. The raw amplicon sequencing data for the synthetic community colonization at 14 days post-inoculation have been deposited in the NCBI BioProject database under accession number PRJNA1429573. The reference sequences for the isolated microbial strains used in this study are available under BioProject PRJNA1433828.
