# Supplementary material for: Cross-niche metabolite–microbiome interactions orchestrate systemic soybean resistance to Fusarium root rot
Source: ISME J. 2026 Mar 27;20(1):wrag080. doi: 10.1093/ismejo/wrag080 (PMC13140568; doi:10.1093/ismejo/wrag080)
Supplement: Supplementary_Information_wrag080 [file supplementary_information_wrag080.docx]

Cross-niche metabolite-microbiome interactions orchestrate systemic soybean resistance to *Fusarium* root rot

Running title: Soybean microbiome-mediated resistance

Qi Liu^1,4^, Lang Cheng^1,4^, Enxi Zhang^1,4^, Li Ling^1^, Weiyi Tan^1^, Suwen Liang^1^, Canwei Shu^1^, Qibin Ma^1^, Shuai Zhao^2^, Jian Wei^3^, Yingxiang Wang^1^, Hai Nian^1,3*^, Yanbo Cheng^1*^, Tengxiang Lian^1*^

1.Guangdong Basic Research Center of Excellence for Precise Breeding of Future Crops, Guangdong Laboratory for Lingnan Modern Agriculture, Guangdong Provincial Key Laboratory for the Development Biology and Environmental Adaptation of Agricultural Organisms, South China Institute for Soybean Innovation Research, College of Agriculture, South China Agricultural University, No.483 Wushan Road, Guangzhou, Guangdong 510642, China

2.State Key Laboratory of Desert and Oasis Ecology, Xinjiang Institute of Ecology and Geography, Chinese Academy of Sciences, Urumqi, China

3.Northern Key Laboratory of Saline-tolerant Soybean Breeding, Ministry of Agriculture and Rural Areas, Jilin Agricultural University

4.These authors contributed equally: Qi Liu, Lang Cheng, Enxi Zhang

Corresponding author:

Tengxiang Lian (lead contact), Email: [liantx@scau.edu.cn](mailto:liantx@scau.edu.cn)

Mailing address: College of Agriculture, South China Agricultural University, No.483 Wushan Road, Guangzhou, Guangdong 510642, China

Hai Nian, Email: [hnian@scau.edu.cn](mailto:hnian@scau.edu.cn)

Mailing address: College of Agriculture, South China Agricultural University, No.483 Wushan Road, Guangzhou, Guangdong 510642, China

Yanbo Cheng, Email: [ybcheng@scau.edu.cn](mailto:ybcheng@scau.edu.cn)

Mailing address: College of Agriculture, South China Agricultural University, No.483 Wushan Road, Guangzhou, Guangdong 510642, China

**Supplementary Information**

**Text S1 Preparation of *F. falciforme* conidial suspension**

*Fusarium falciforme* was maintained on potato dextrose agar (PDA), and its conidial suspension was prepared by modifying a previously described method [1]. Specifically, a 1-cm² mycelial plug from an *F. falciforme* culture grown on PDA was transferred to potato dextrose broth (PDB) and incubated at 28°C with orbital shaking at 160 rpm. After 7 days of incubation, conidia were harvested via filtration to remove mycelia and medium debris, followed by washing with sterile double-distilled water (ddH_2_O). Over 90% of the harvested propagules were identified as microconidia. Additionally, the sequence information for this *F. falciforme* strain has been deposited in the NCBI database under the accession number MZ067794.

**Text S2 Wounding inoculation**

The root-wounding procedure was adapted from a previously described method [2], briefly, three to five longitudinal incisions (approximately 0.5 mm in depth) were artificially made on the lower half of the primary root using a sterile disposable scalpel. Subsequently, the wounded roots were inoculated via the root-soaking method in the pathogen suspension pool. This procedure ensured that the vascular tissues (cortex and vascular bundles) were directly exposed to the inoculum, simultaneously maintaining the structural integrity of the root system to prevent non-pathogenic mortality caused by excessive physical trauma.

**Text S3 qPCR methodology and standard curve validation**

1. Standard curve construction

The target fragment of *F. falciforme* was amplified and ligated into the *p*MD18-T vector (2,692 bp). The concentration of the purified recombinant plasmid was determined using a NanoDrop 2000. Molar concentration was converted to genomic copy numbers based on the total molecular weight (Vector + Insert). A 10-fold serial dilution was performed to establish a standard curve with high linearity (*R*^2^ > 0.99) and optimal amplification efficiency (90%–110%). All assays were conducted in triplicate.

1. qPCR protocols

Each 25 µL qPCR mixtures contained 12.5 µL of 2X TB Green Premix Ex Taq II (Tli RNaseH Plus) (Takara), 1 µL of each primer (10 µM; Fal-qF/Fal-qR), 2 µL of template DNA, and 8.5 µL of nuclease-free water. Thermal cycling was performed as follows: initial denaturation at 95°C for 30 s, followed by 40 cycles of denaturation at 95°C for 5 s, annealing at 60°C for 30 s, and extension at 72°C for 30 s. A melting curve analysis was conducted at the end of each run to verify amplification specificity.

**Supplementary figures**


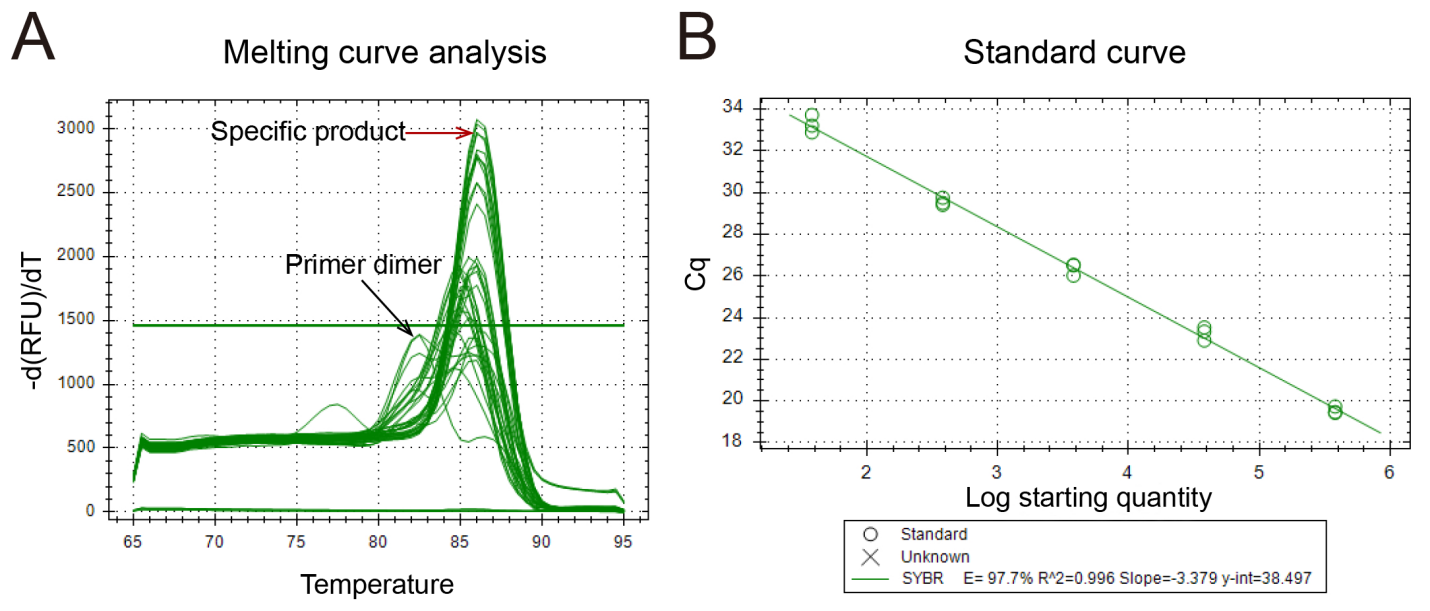


**Fig. S1.** Validation of the qPCR assay for *F. falciforme* quantification. (A) Melting curve analysis of the designed primers. Target pathogen (red) show a single, sharp peak, confirming high amplification specificity for the target pathogen. Non-inoculated (CK) samples (black) show no specific peaks, with only non-specific signals (primer dimers) appearing at Cq > 35. (B) Validation of the plasmid-based standard curve. The standard curve exhibited high linearity (*R*^2^ > 0.99) and an amplification efficiency of 97.7% (slope = -3.379) for *F. falciforme* quantification. These parameters fall within the optimal range (Efficiency: 90–110%; *R*^2^ > 0.99), confirming the assay's high sensitivity and accuracy for biomass calculation.


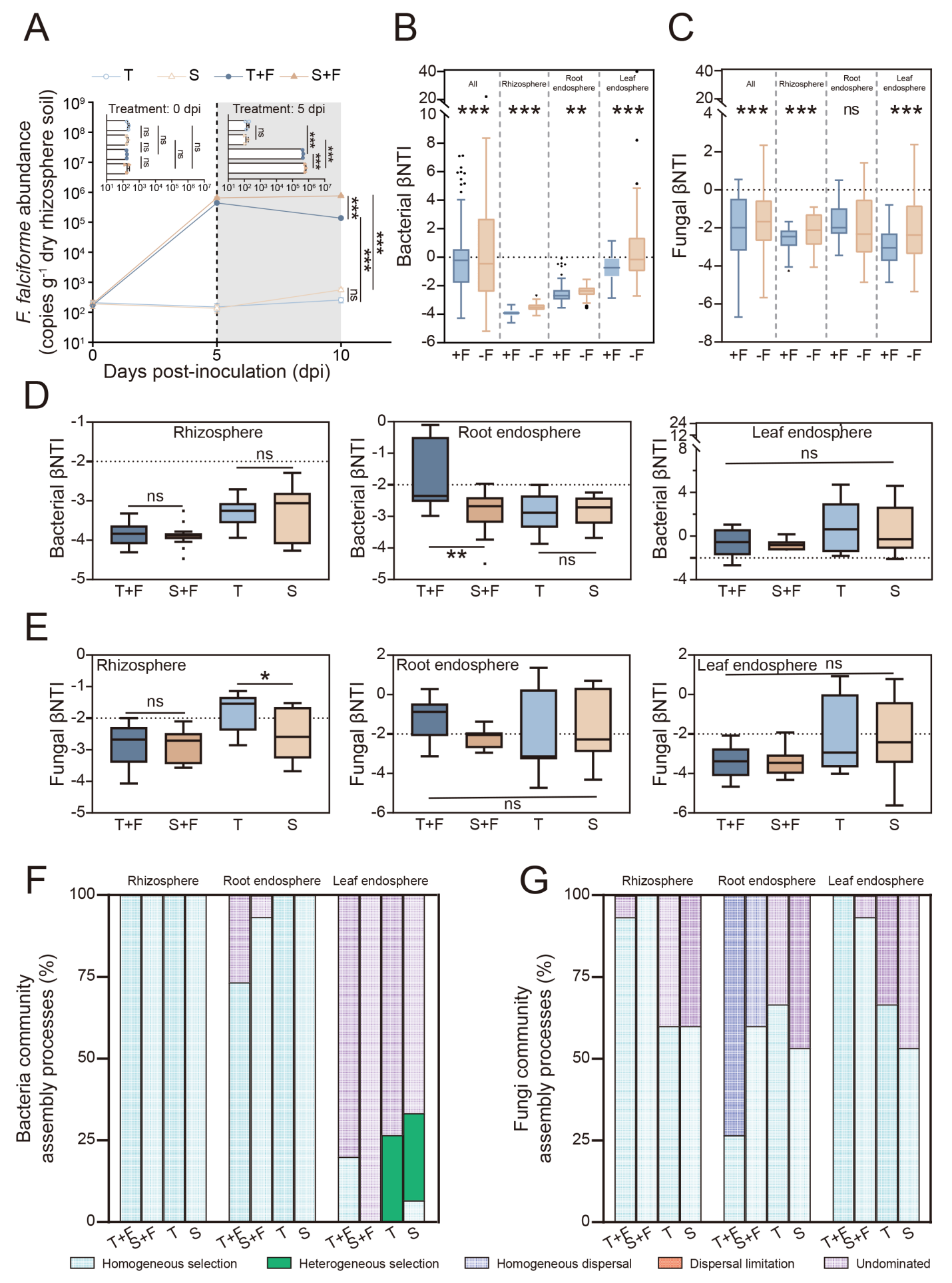


**Fig. S2.** Pathogen load and community-assembly metrics. (A) Pathogen abundance of *F. falciforme* in the rhizosphere of tolerant (GXD2, T) and susceptible (ND12, S) soybeans over a 10-day period. The main line graph illustrates the colonization trend, with significant differences between treatments at 10 days post-inoculation (dpi) indicated by asterisks. The two insets (bar charts) provide detailed comparisons at 0 and 5 dpi, respectively. Four treatments are compared: non-inoculated controls (T and S) and inoculated groups (T+F and S+F). Data are presented as mean ± SEM (n = 3 biological replicates). Statistical significance was determined by one-way ANOVA (* *P* < 0.05; ** *P* < 0.01; *** *P* < 0.001; ns, not significant). (B, C) βNTI values for bacterial (B) and fungal (C) communities in inoculated (+F) versus uninoculated (–F) plants, reflecting phylogenetic turnover. (D, E) βNTI for bacteria (D) and fungi (E) in tolerant versus susceptible soybeans under +F and –F treatments, resolved by compartment. Asterisks denote significance (* *P* < 0.05; ** *P* < 0.01; *** *P* < 0.001, one-way ANOVA). (F, G) Relative contribution of community-assembly processes—variable selection (VS, βNTI > 2), homogenizing selection (HS, βNTI < –2) and stochastic drift/dispersal (DDH, |βNTI| < 2)—in tolerant and susceptible plants across the three compartments.


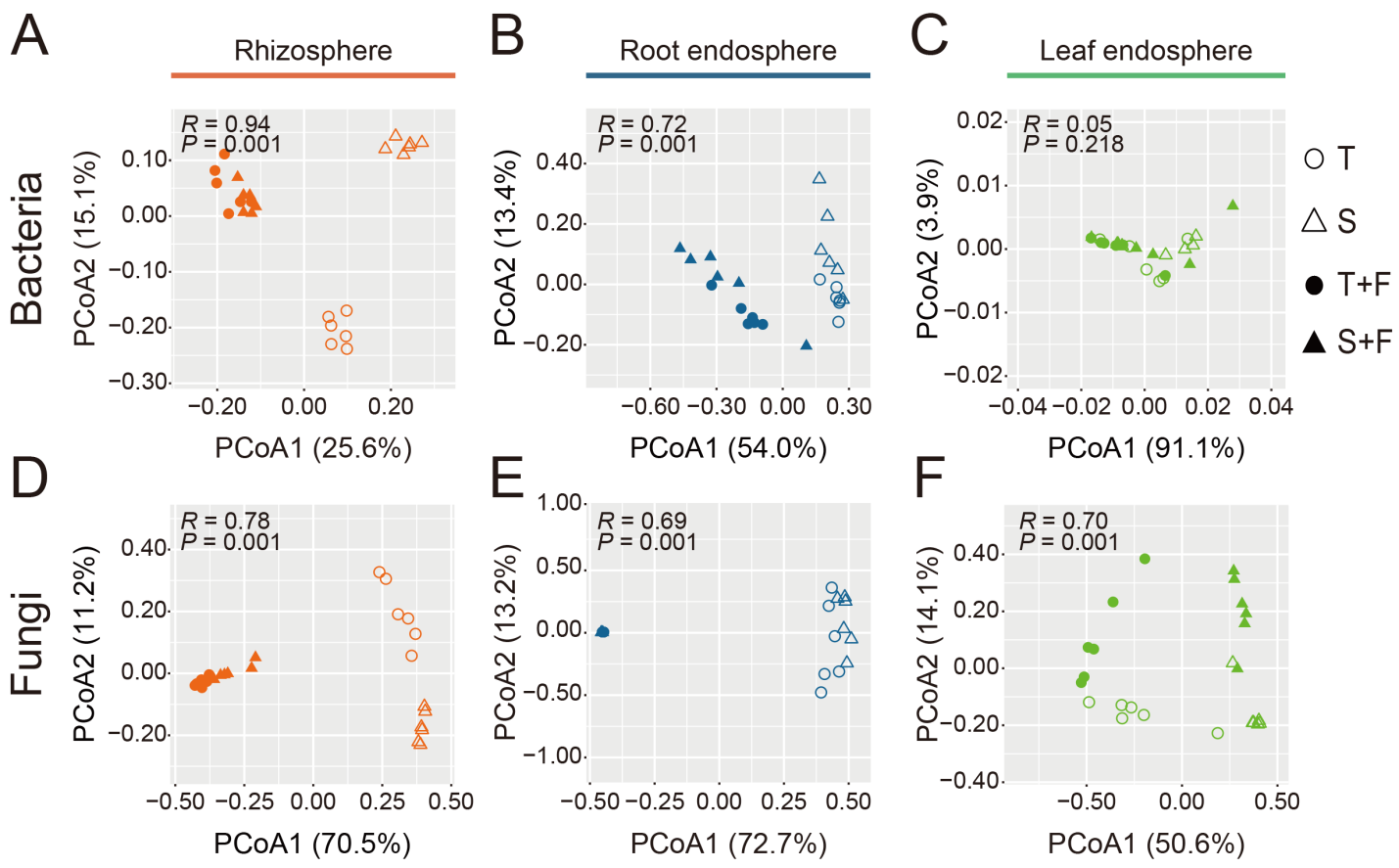


**Fig. S3.** PCoA of microbial community structures in response to *F*. *falciforme* inoculation. Principal Coordinate Analysis (PCoA) based on Bray-Curtis dissimilarities illustrating the clustering patterns of (A-C) bacterial and (D-F) fungal communities across the rhizosphere, root endosphere, and leaf endosphere. Circles represent the tolerant variety T and triangles represent the sensitive variety S. Open symbols indicate non-inoculated controls whereas solid symbols (+F) indicate inoculation with *Fusarium*. Percentages on axes represent the variance explained by PCoA1 and PCoA2. The *R* and *P* values were derived from ANOSIM to indicate the degree of community dissimilarity among varieties and inoculation treatments.


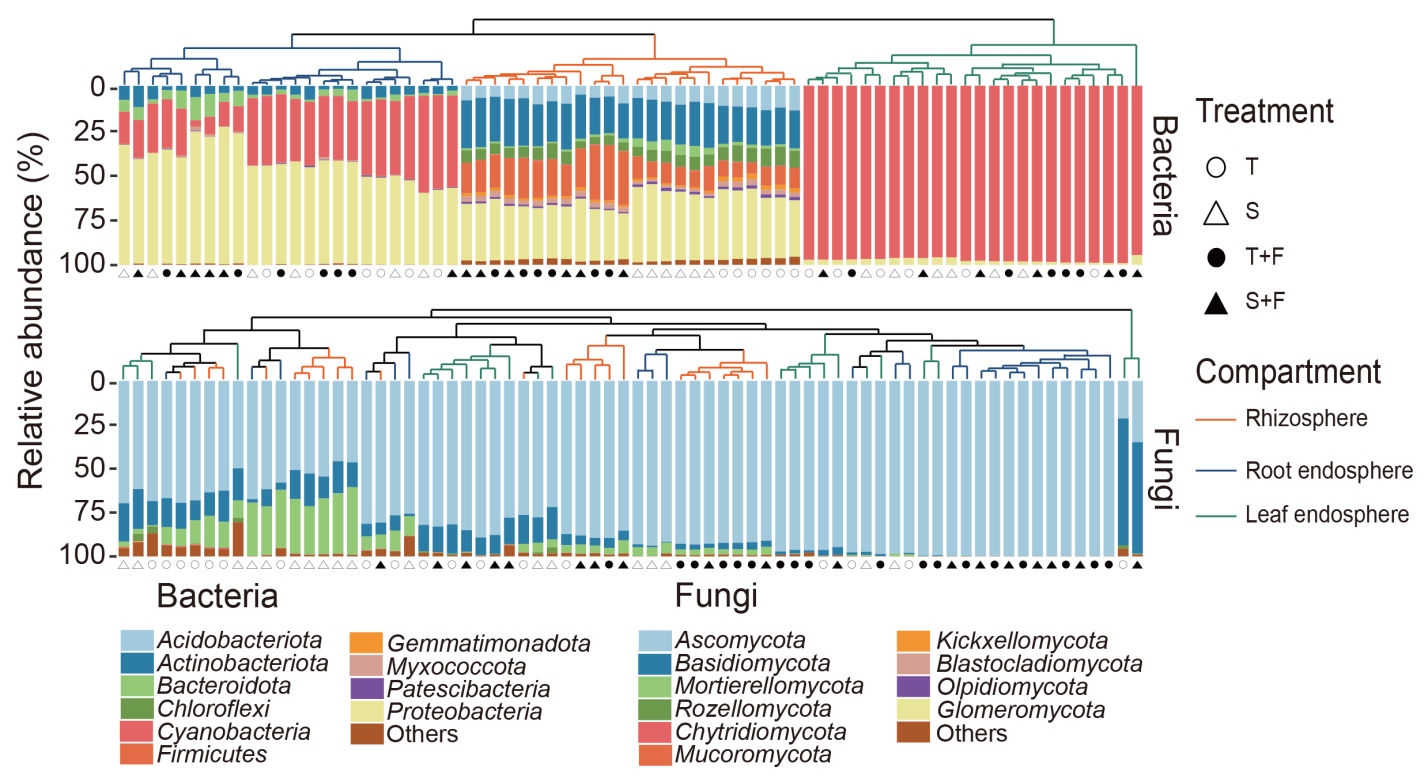


**Fig. S4.** Phylum-level composition of bacterial and fungal communities across treatments. Stacked bar charts show the relative abundance of bacterial (top) and fungal (down) phyla in all 72 samples. Hierarchical clustering of Bray–Curtis distances (Ward.D2 linkage) orders the samples along the x-axis. Phyla representing < 1% of total sequences are pooled into “Others.”


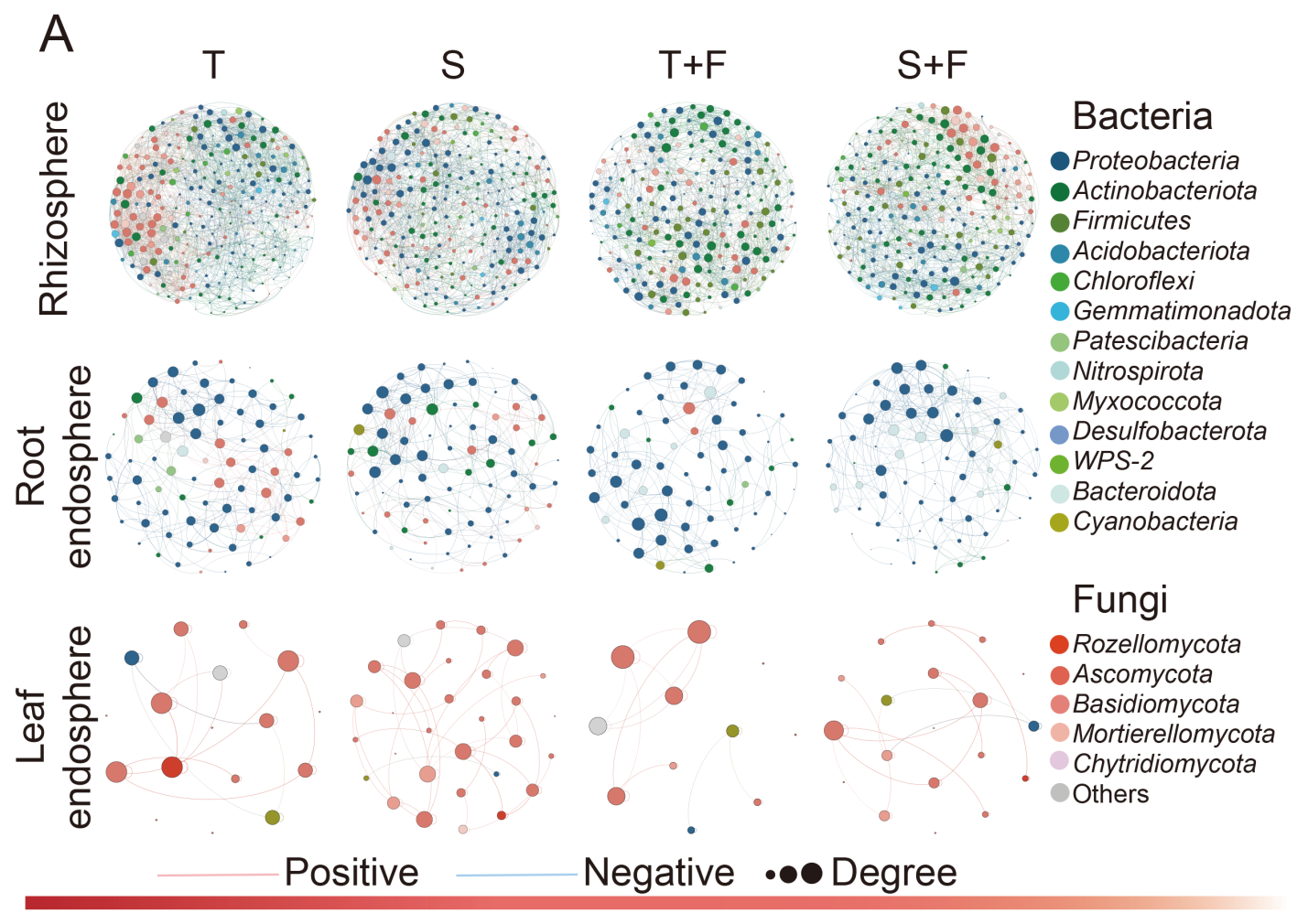


**Fig. S5.** Cross-kingdom co-occurrence networks in tolerant (T) and susceptible (S) soybeans with (+F) or without (–F) *F. falciforme* challenge. (A) Co-occurrence networks depicting intra-kingdom interactions within bacterial and fungal communities across rhizosphere, root endosphere, and leaf endosphere niches. Each node represents an ASV; node size is proportional to its degree (number of edges). Node colors correspond to the dominant phylum (key at right). Edges indicate significant Spearman correlations (*ρ* > 0.7, *P* < 0.05); pink, positive links; blue, negative links.

**
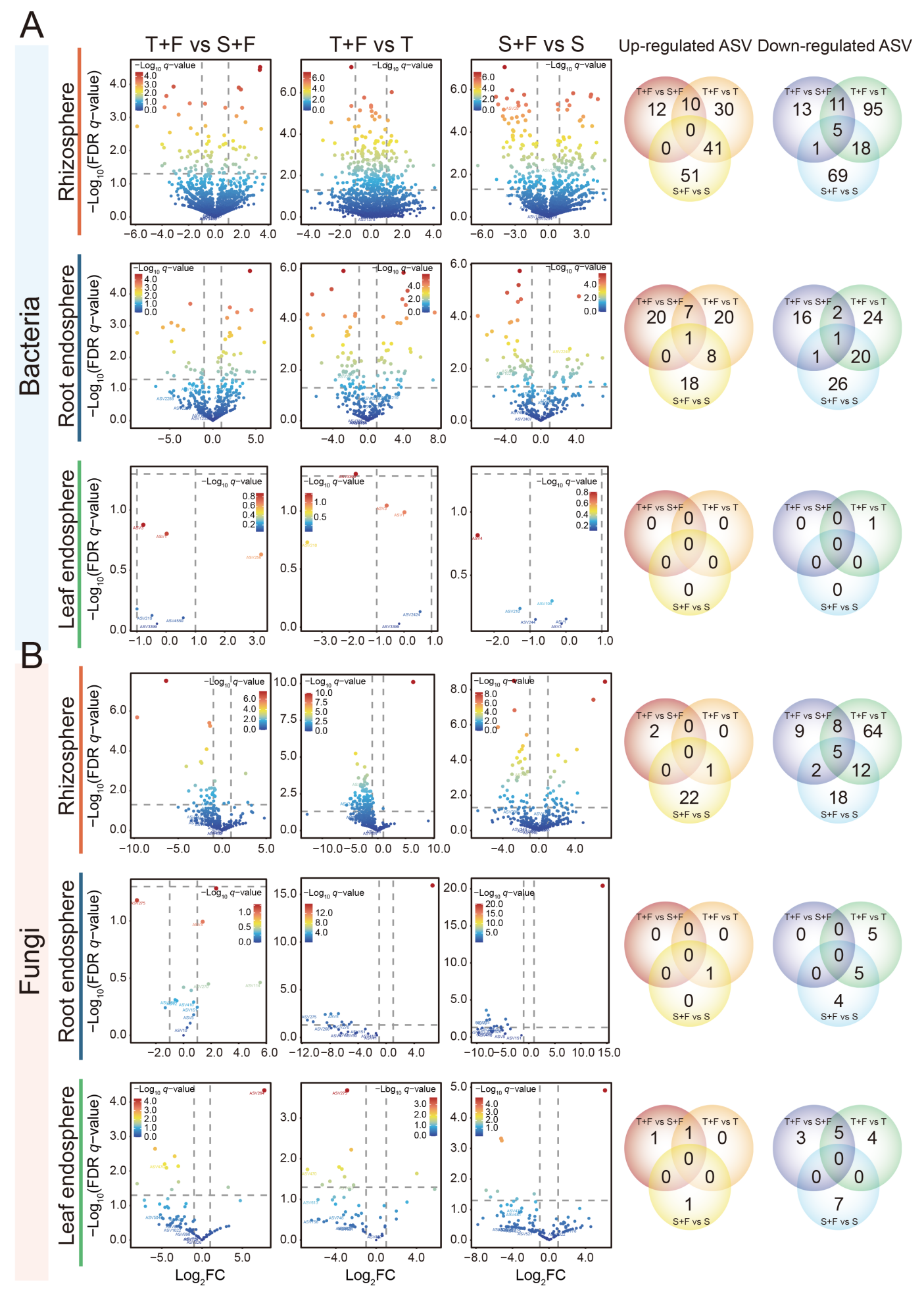
**

**Fig. S6.** ASV-level shifts driven by *F. falciforme* differ by genotype and plant compartment. Volcano plots dissect differential ASVs of bacteria (A) and fungi (B) across three critical contrasts: genotypic divergence under stress (T+F vs S+F; tolerant vs susceptible under *F. falciforme* inoculation), resistance-specific restructuring (T+F vs T; *F. falciforme*-induced microbiome remodeling), and susceptibility-associated dysbiosis (S+F vs S; *F. falciforme*-triggered community destabilization). Points to the right of the centre line are enriched, those to the left are depleted; vertical position reflects statistical support (–log_₁₀_ *q*-value). Venn diagrams summarise the overlap of up-regulated and down-regulated ASVs among the three comparisons, illustrating genotype- and compartment-specific recruitment versus loss of taxa in response to *F. falciforme*.

**
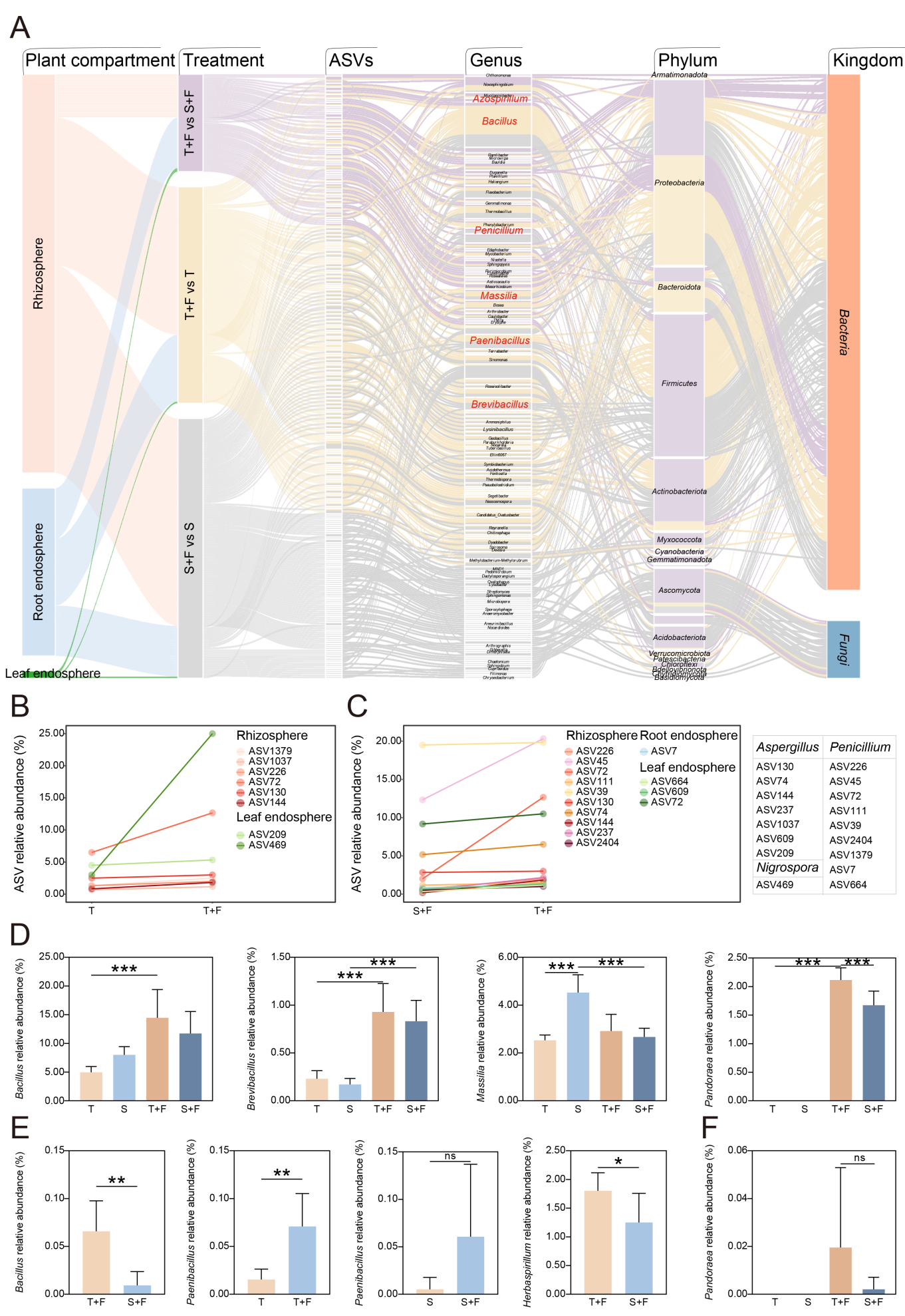
**

**Fig. S7.** Compartment-resolved recruitment of key taxa in response to *Fusarium falciforme*. (A) Sankey diagram traces ASVs significantly enriched in tolerant (T + F) versus susceptible (S + F) plants across the rhizosphere, root endosphere, and leaf endosphere (log_2_FC ≥ 1, *P* < 0.05). Band width is proportional to summed relative abundance. (B) Fungal ASVs whose abundance rises after infection in the tolerant cultivar (T + F vs T). (C) Fungal ASVs that are more abundant in the tolerant than the susceptible cultivar under infection (T + F vs S + F). (D) Relative abundance of *Bacillus*, *Brevibacillus*, *Massilia*, and *Pandoraea* in the rhizosphere under the four treatment combinations (T, T + F, S, S + F). (E) Relative abundance of *Bacillus*, *Paenibacillus*, and *Herbaspirillum* in the root endosphere. (F) Relative abundance of *Pandoraea* in the leaf endosphere. Bars represent means ± SD (n = 6). ns, not significant; *P* < 0.05 (*), < 0.01 (**), < 0.001 (***); one-way ANOVA with post-hoc Tukey test.


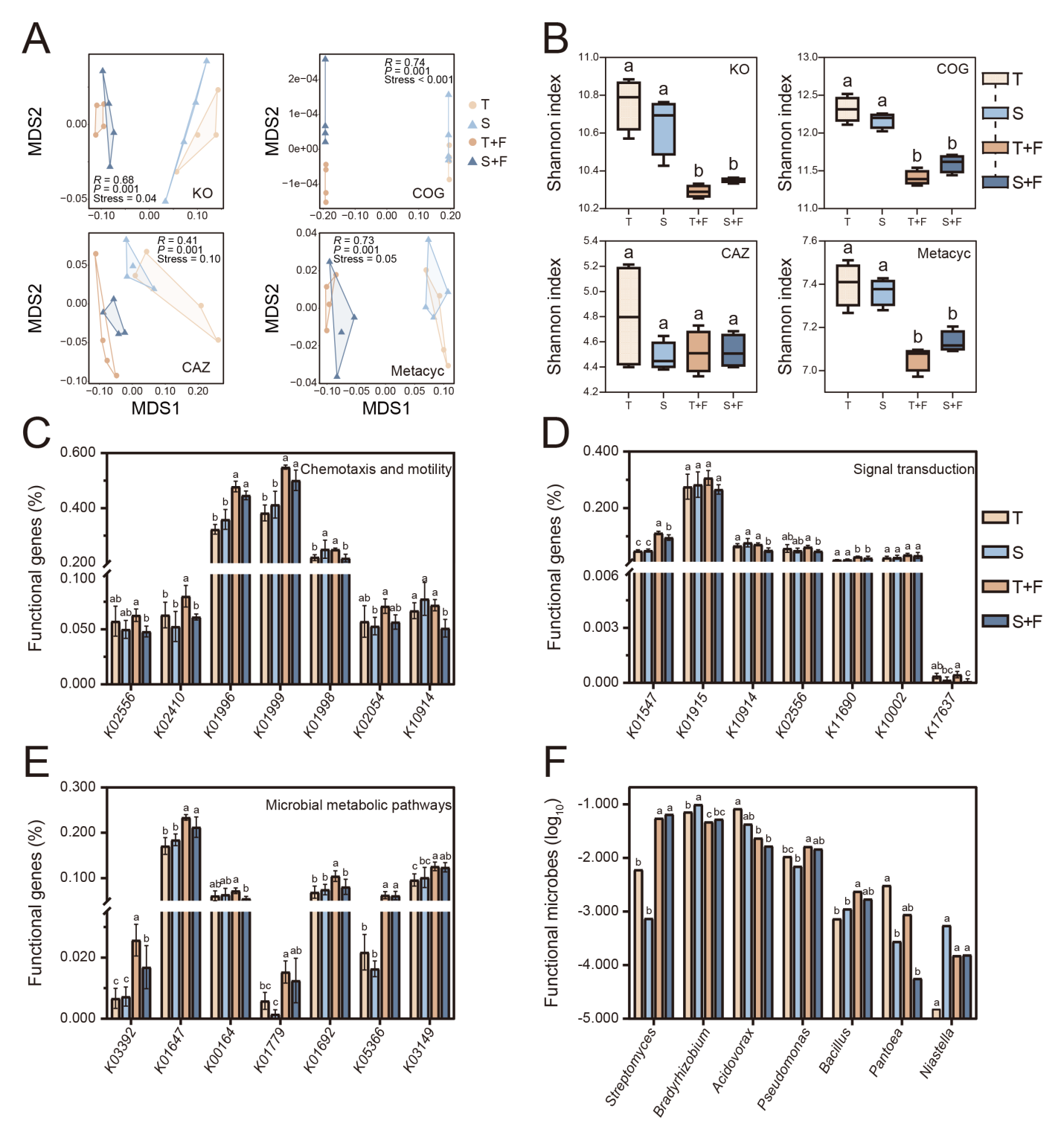


**Fig. S8.** Metagenome-level functional shifts under *Fusarium falciforme* challenge. (A) Non-metric multidimensional scaling (NMDS) ordinations (Bray–Curtis) of KO, COG, CAZy, and MetaCyc profiles show that infection separates functional repertoires of tolerant (T) and susceptible (S) cultivars. (B) Shannon index of the four annotation sets. Boxes display median ± IQR; letters denote significant differences (one-way ANOVA, *P* < 0.05). (C–E) Bar plots of key KO marker genes: (C) chemotaxis/motility, (D) signal transduction, (E) core metabolic pathways. Means ± SD, *n* = 4. (F) Contribution of the seven most abundant genera to "other secondary-metabolite biosynthesis" (KEGG) across treatments. Different lowercase letters indicate significant pairwise differences (*P* < 0.05, Tukey’s test).


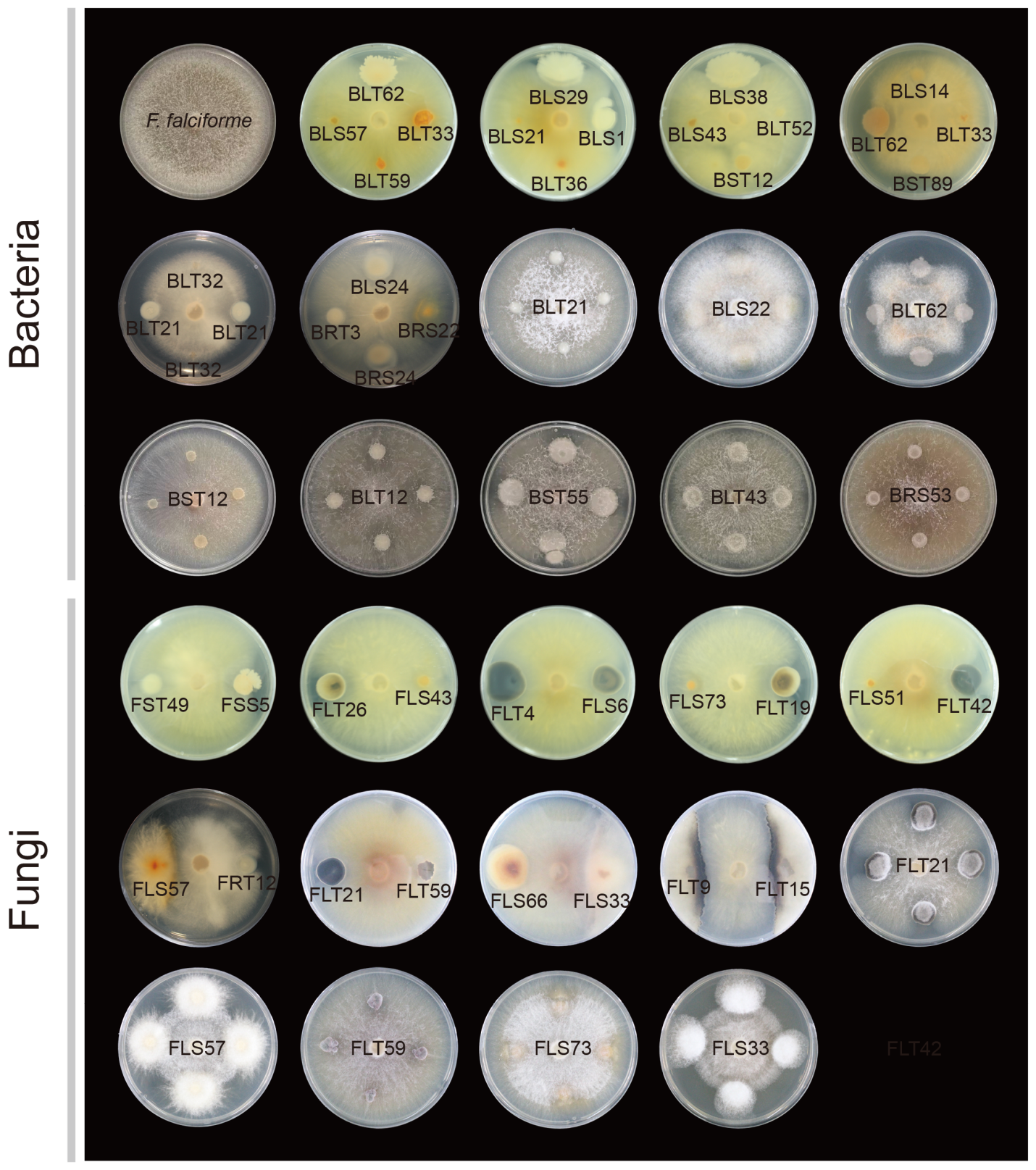


**Fig. S9.** Antagonistic activity of compartment-derived isolates against *F. falciforme.* Dual-culture assays on PDA plates illustrate representative inhibition halos produced by selected bacterial (upper panels) and fungal (lower panels) isolates obtained from the rhizosphere, root endosphere, and leaf endosphere. B, Bacteria; F, Fungi; S, Rhizosphere; R, Root endosphere; L, Leaf endosphere.


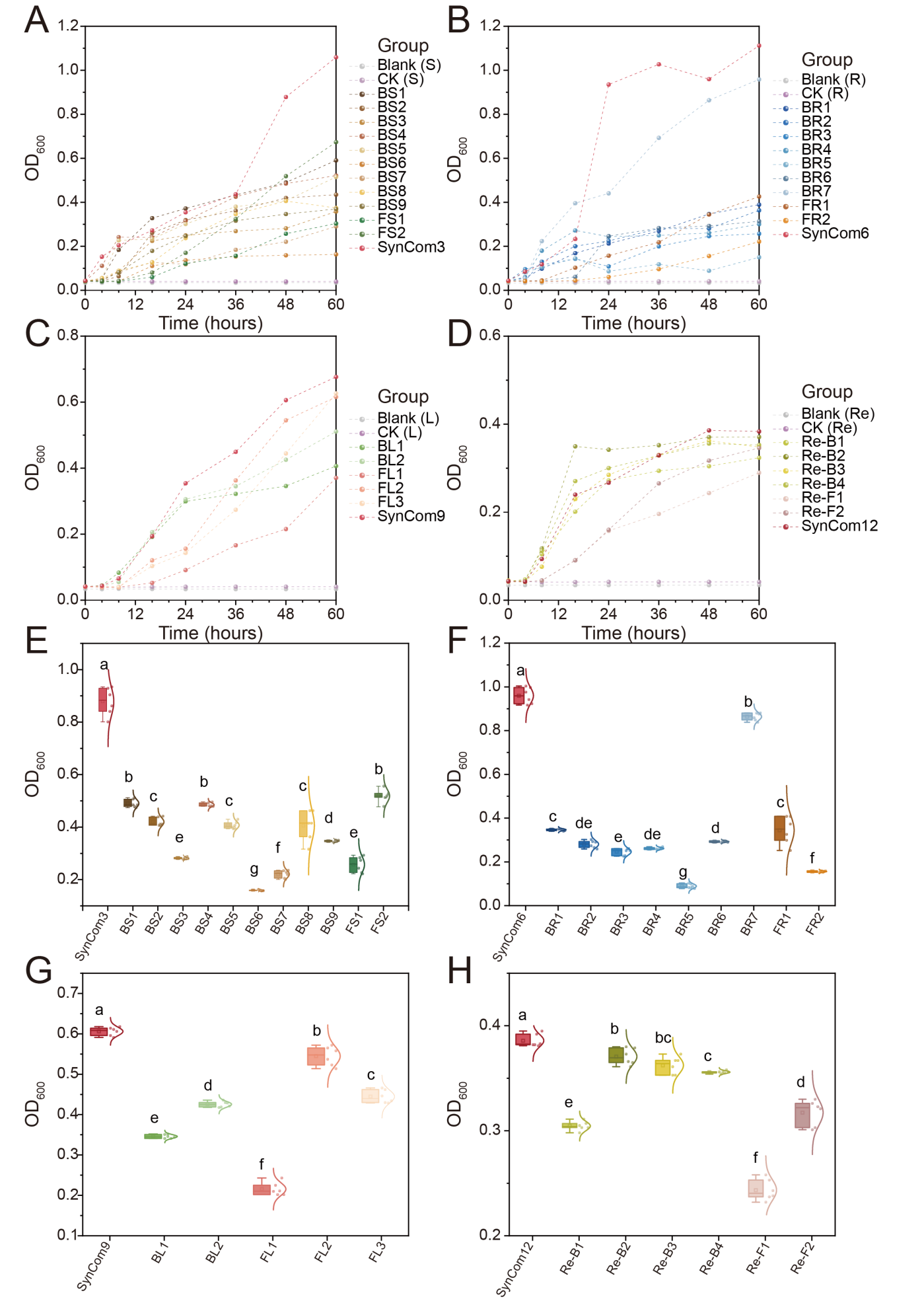


**Fig. S10.** Growth dynamics and biomass accumulation (OD_600_) of SynComs and their constituent strains. (A-D) Growth trajectories of SynComs and individual member strains over a 60-hour incubation period. B, Bacteria; F, Fungi; S, Rhizosphere; R, Root endosphere; L, Leaf endosphere; Re/Re-, Antagonistic strains against *F. falciforme*. Blank and CK represent the negative and medium controls, respectively. (E–H) OD_600_ values at 48 hours for the top-down constructed rhizospheric (SynCom3, E), root endophytic (SynCom6, F), and leaf endophytic (SynCom9, G) communities, and the bottom-up constructed SynCom12 (H), compared with their respective individual strains (n = 6). Different lowercase letters denote significant differences among treatments (one-way ANOVA, *P* < 0.05).


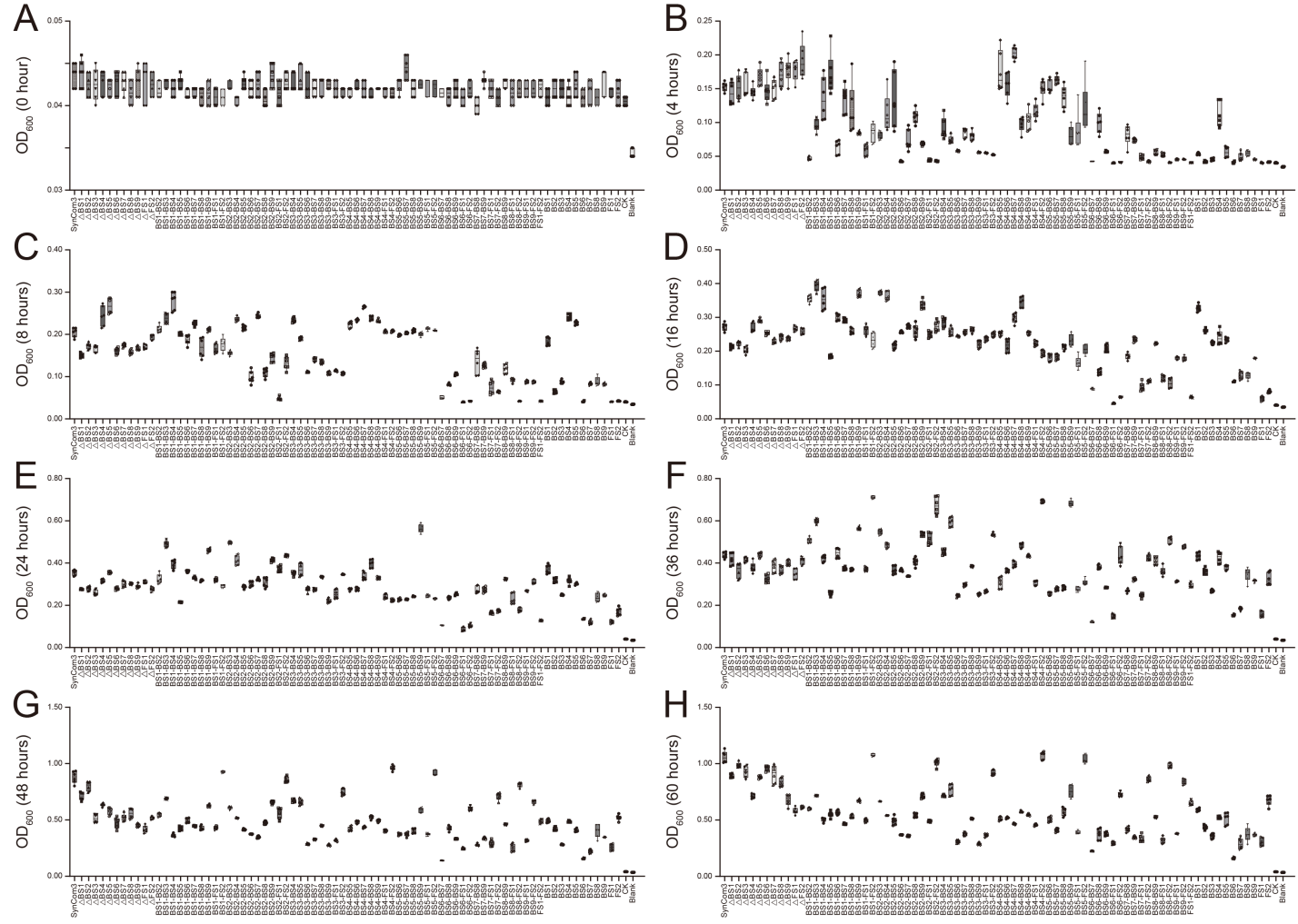


**Fig. S11.** Dynamic growth profiles of the rhizospheric SynCom and its constituent assemblages. (A–H) Box plots of OD_600_ values for the SynCom, leave-one-out groups, pairwise co-cultures, and single strains across 0 to 60 hours (n = 6). Statistical significance was assessed using one-way ANOVA. Full statistical details are available on GitHub (https://github.com/liuqi0619/*Fusarium*-root-rot-in-soybean).


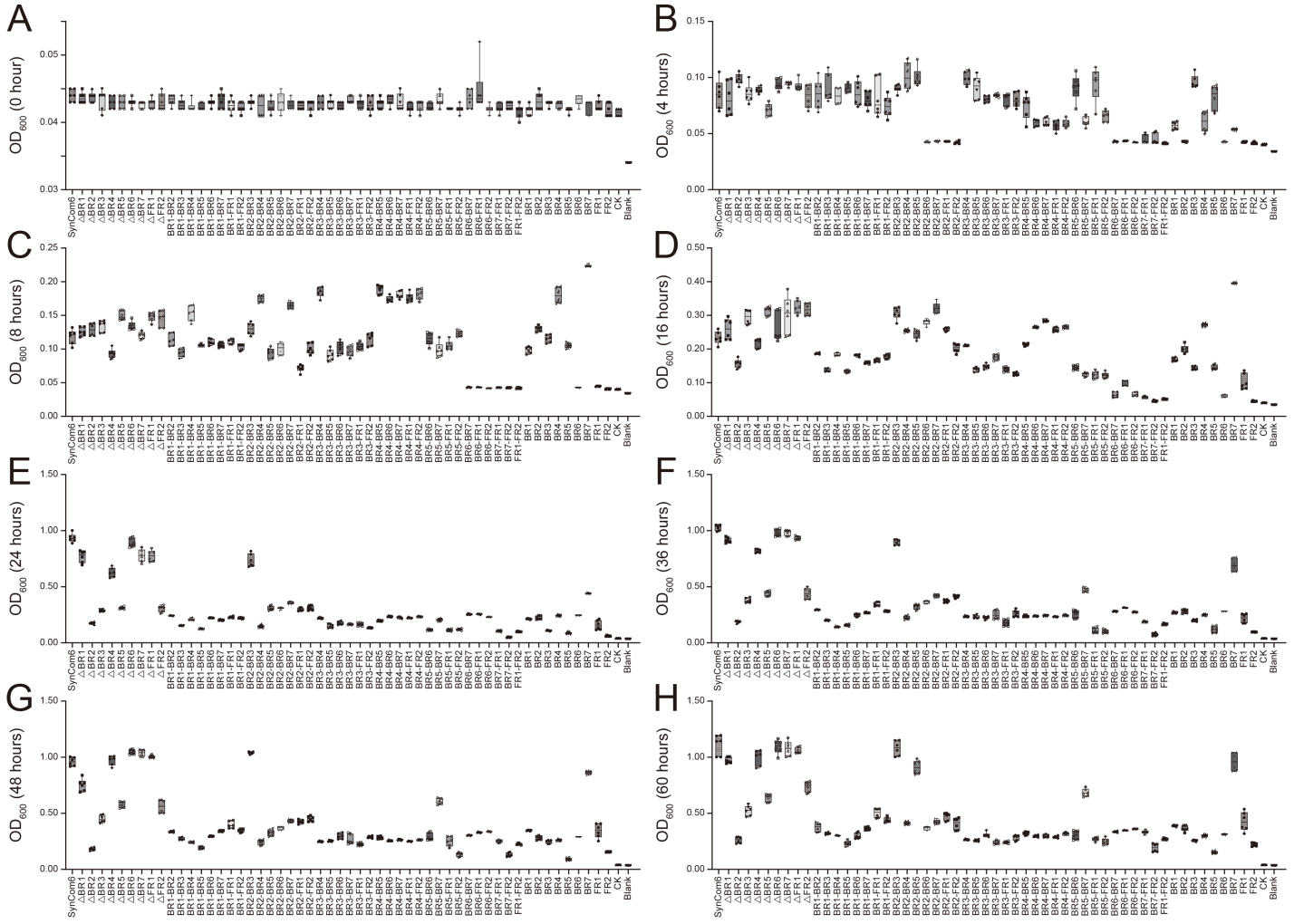


**Fig. S12.** Dynamic growth profiles of the root endophytic SynCom and its constituent assemblages. (A–H) Box plots of OD_600_ values for the SynCom, leave-one-out groups, pairwise co-cultures, and single strains across 0 to 60 hours (n = 6). Statistical significance was assessed using one-way ANOVA. Full statistical details are available on GitHub (https://github.com/liuqi0619/*Fusarium*-root-rot-in-soybean).


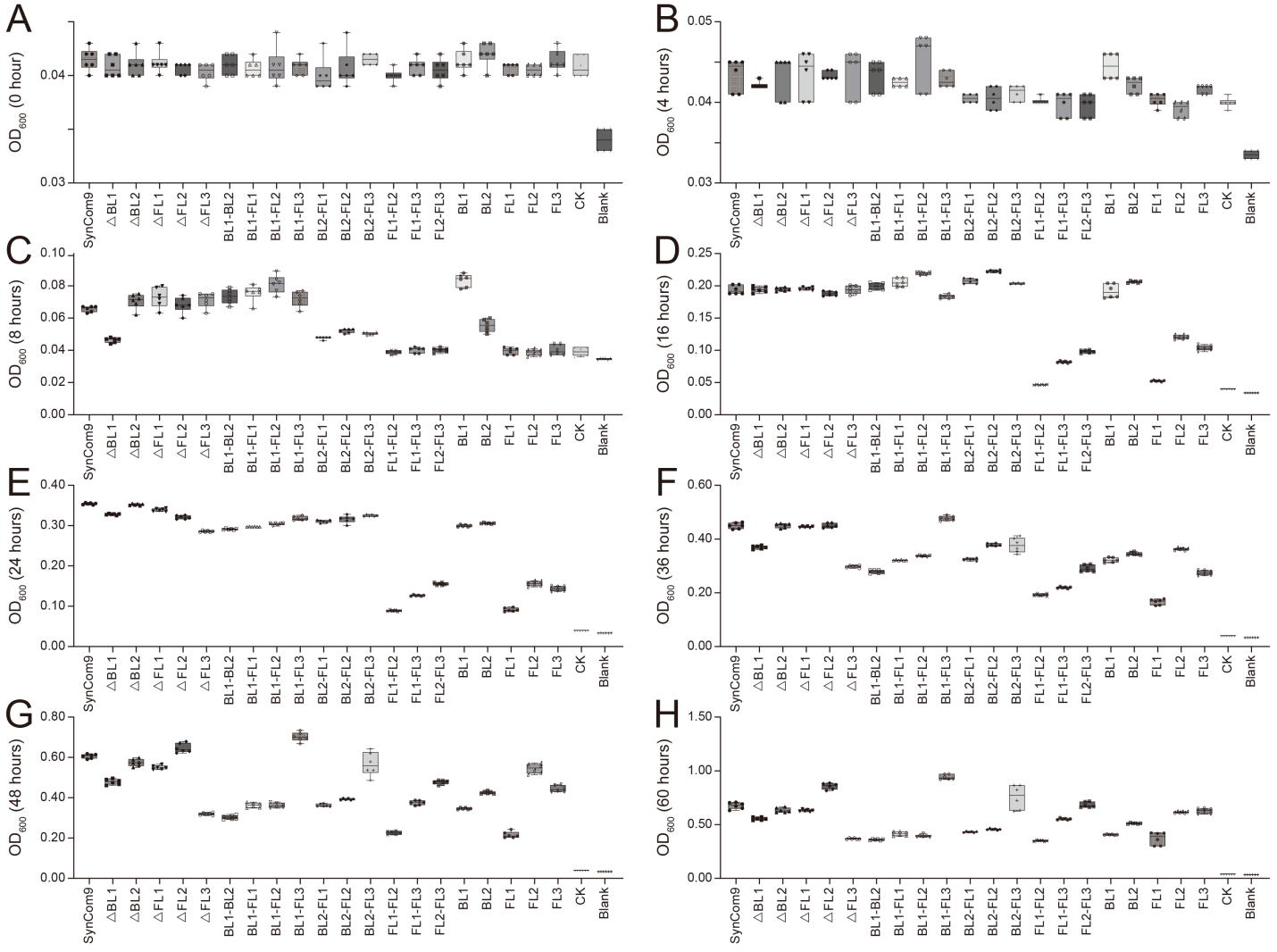


**Fig. S13.** Dynamic growth profiles of the leaf endophytic SynCom and its constituent assemblages. (A–H) Box plots of OD_600_ values for the SynCom, leave-one-out groups, pairwise co-cultures, and single strains across 0 to 60 hours (n = 6). Statistical significance was assessed using one-way ANOVA. Full statistical details are available on GitHub (https://github.com/liuqi0619/*Fusarium*-root-rot-in-soybean).


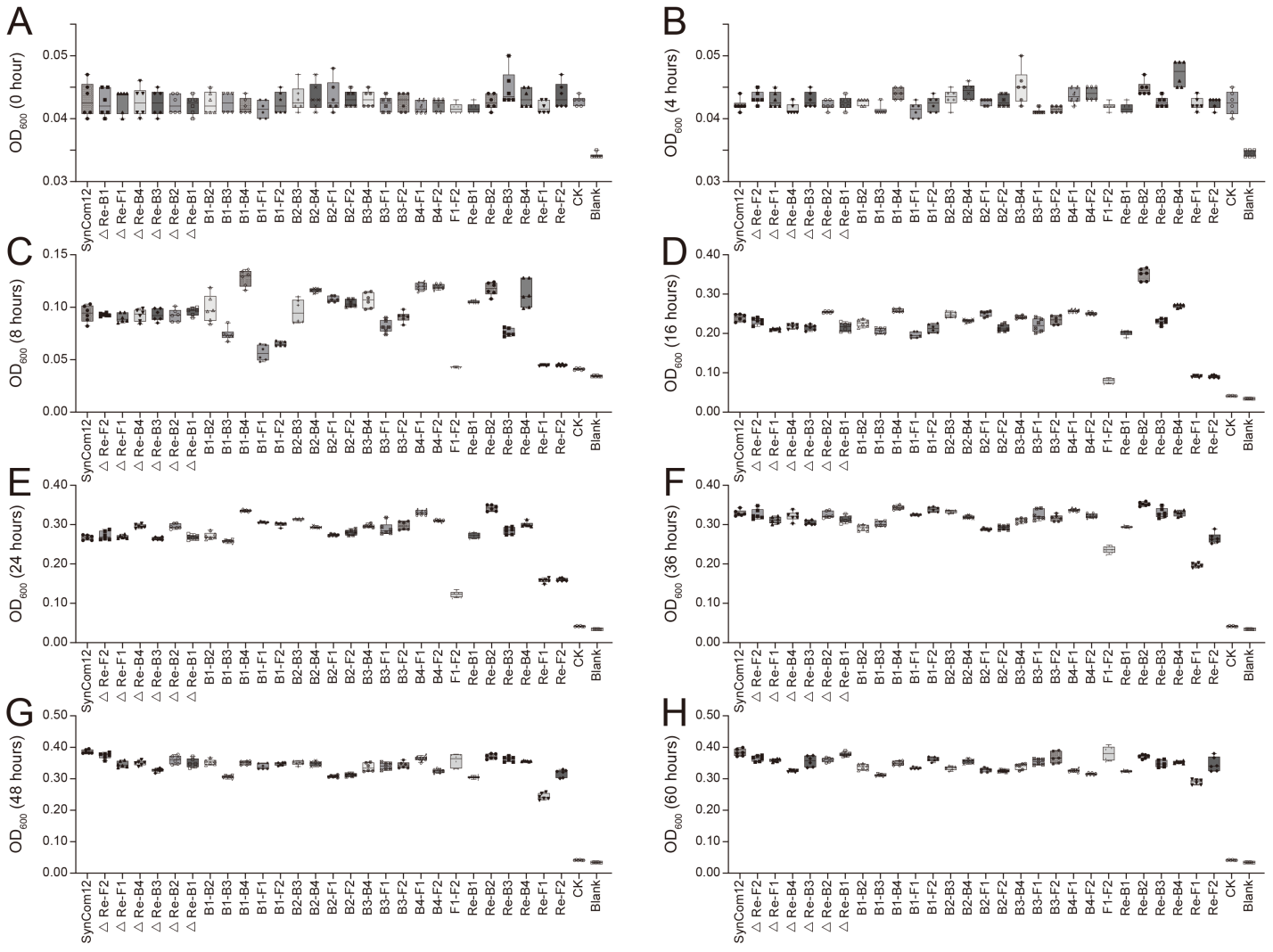


**Fig. S14.** Dynamic growth profiles of the bottom-up designed antagonistic SynCom and its constituent assemblages. (A–H) Box plots of OD_600_ values for the *F. falciforme*-antagonistic SynCom (SynCom12), leave-one-out groups, pairwise co-cultures, and single strains across 0 to 60 hours (n = 6). Statistical significance was assessed using one-way ANOVA. Full statistical details are available on GitHub (https://github.com/liuqi0619/*Fusarium*-root-rot-in-soybean).


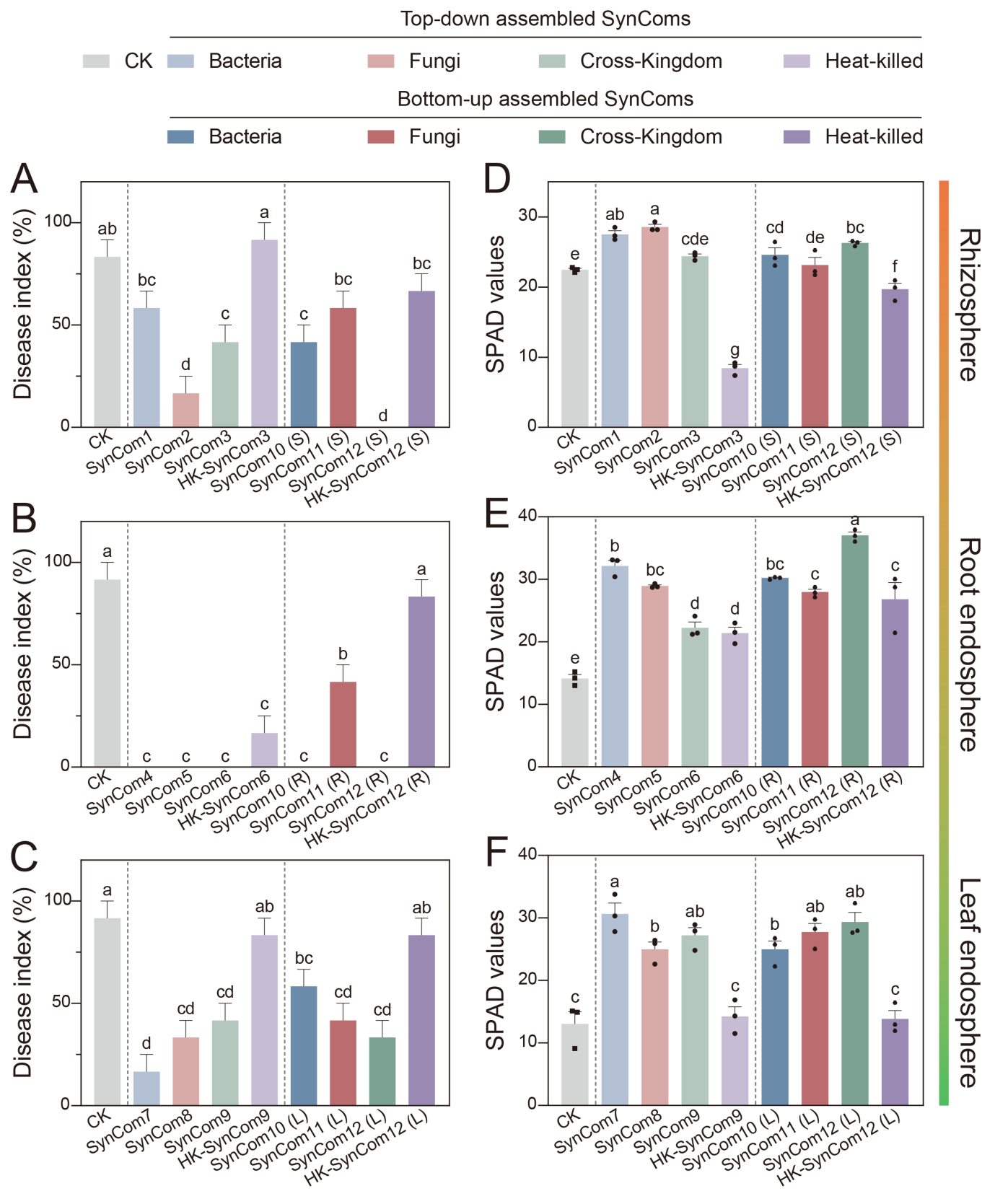


**Fig. S15**. SynCom-mediated mitigation of *F. falciforme* root-rot symptoms in susceptible soybean. Disease index (A-C) and leaf chlorophyll content (SPAD value, D-F) recorded 14 days after inoculation with the indicated synthetic communities applied to the rhizosphere, root endosphere, or leaf endosphere. Bars represent means ± SD (n = 3, four plants per pot). Different lowercase letters denote significant differences among treatments (one-way ANOVA, *P* < 0.05).


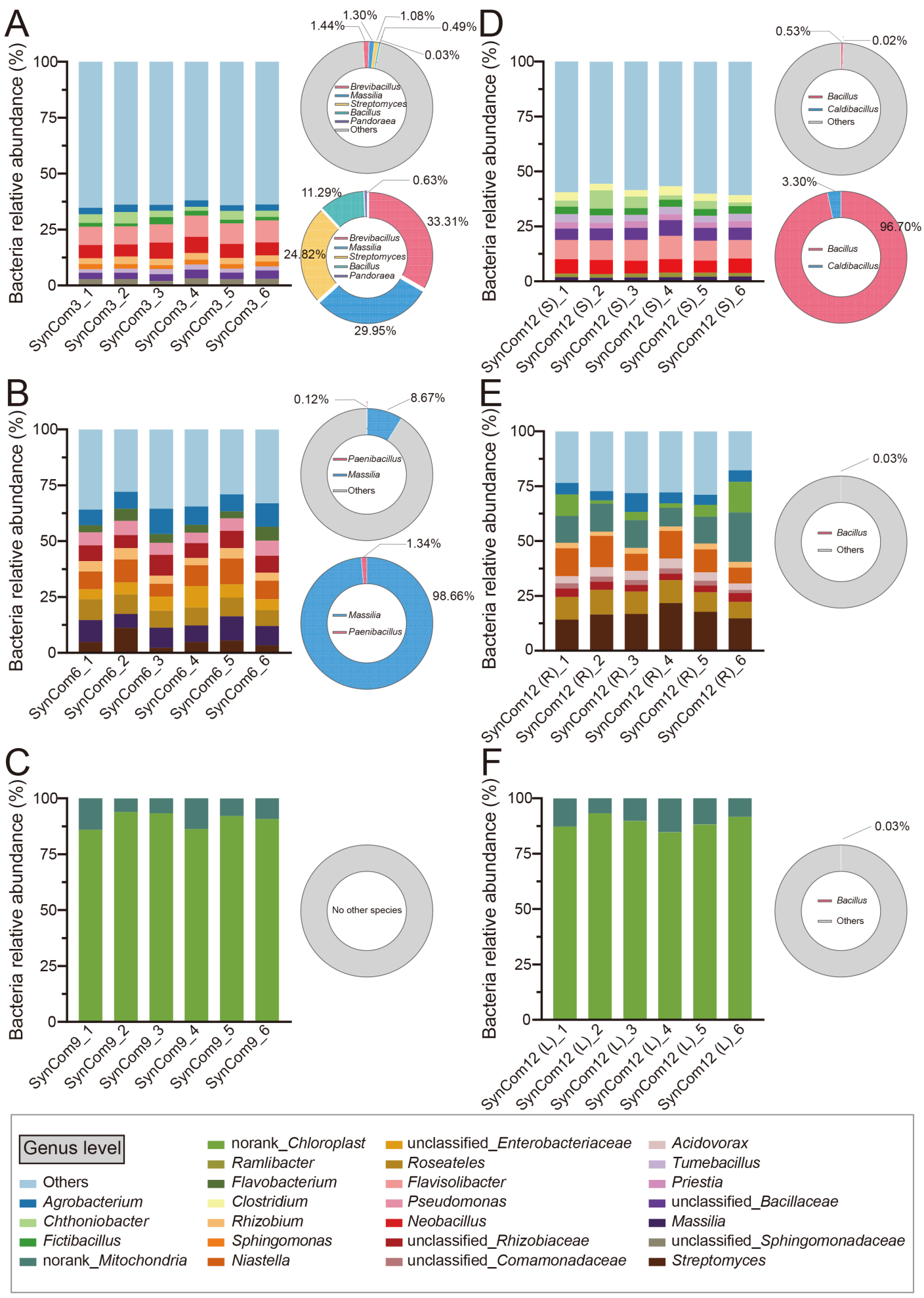


**Fig. S16.** Taxonomic profiles and colonization of SynComs following inoculation. (A–C) Genus-level composition (top 10 abundant genera) in the rhizosphere (S), root endosphere (R), and leaf endosphere (L) after inoculation with SynCom3, 6, and 9. (D–F) Profiles following inoculation with SynCom12. Stacked bars show total bacterial relative abundance. Adjacent pie charts detail SynCom colonization: above, combined relative abundance of all target strains within the whole community; below, relative proportions among individual target strains within the SynCom. Strains below the detection limit are omitted.


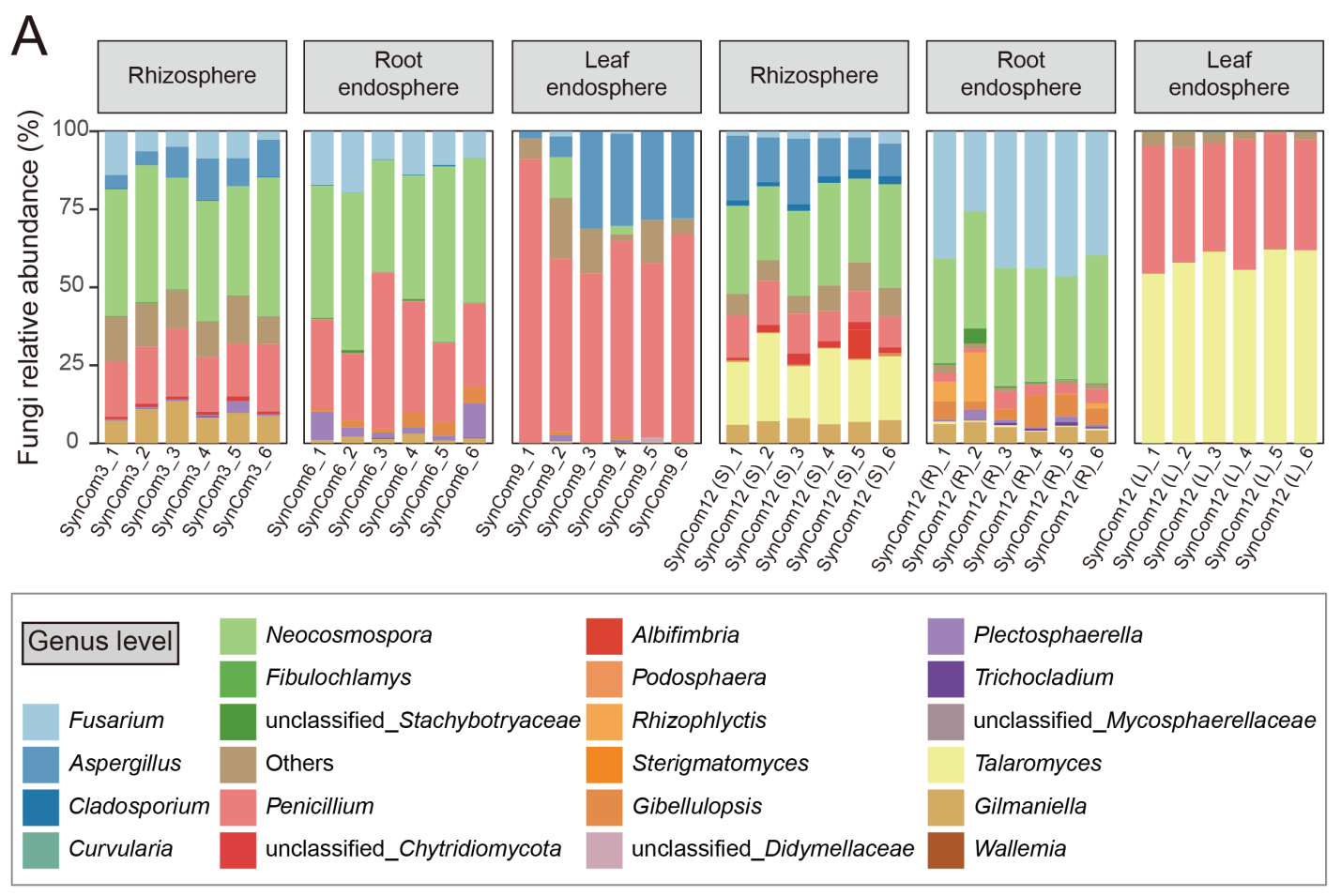


**Fig. S17.** Fungal genus-level composition following SynCom inoculation. (A) Relative abundance of fungal genera in the rhizosphere, root endosphere, and leaf endosphere across different SynCom treatments (SynCom3, 6, 9, and 12). Each stacked bar represents an individual biological replicate (n = 6). Colors correspond to the fungal genera as indicated in the legend.


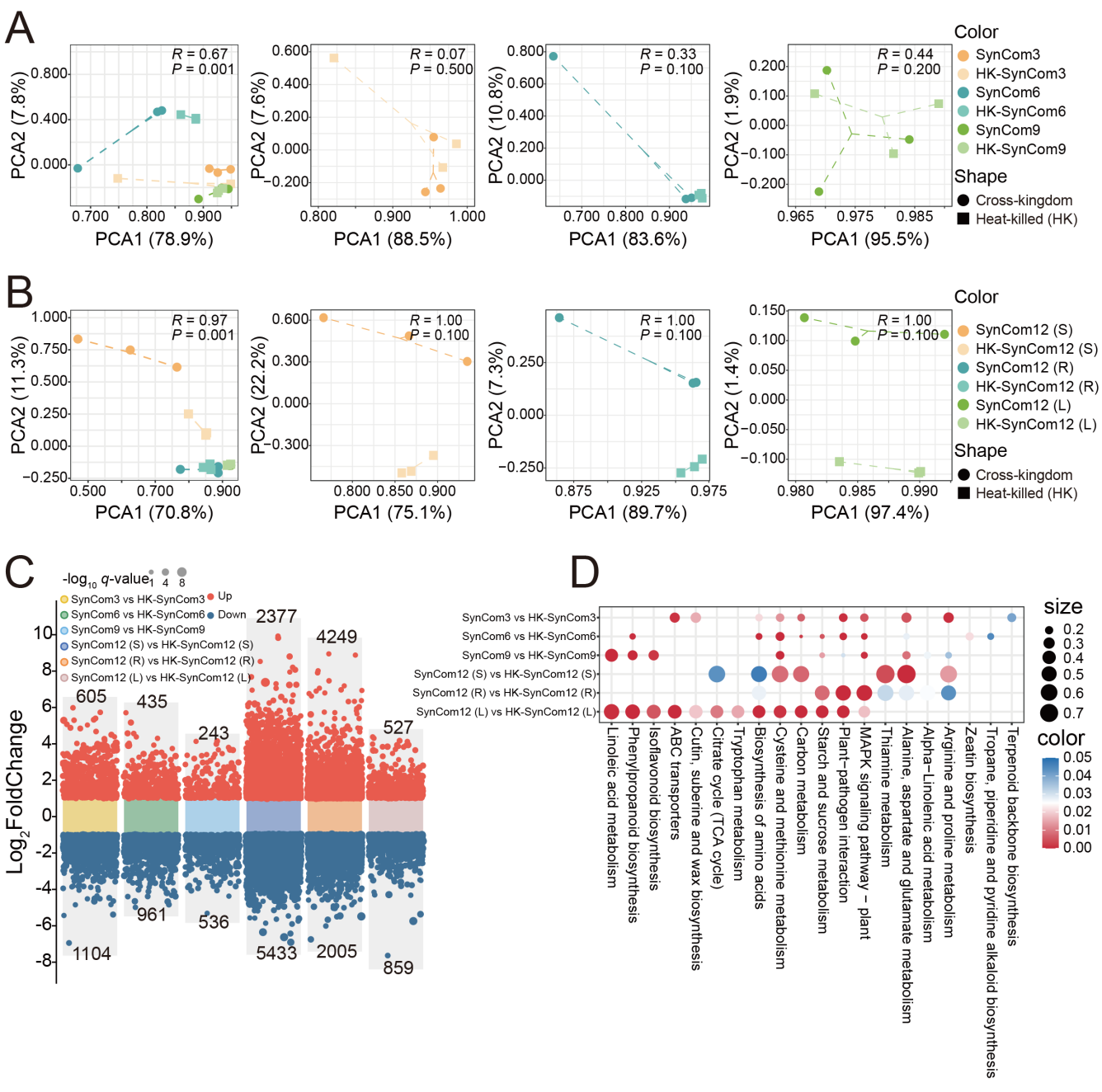


**Fig. S18**. Root-transcriptome reprogramming triggered by live versus heat-killed cross-kingdom SynComs. (A) Principal-component analysis (PCA) of root transcriptomes from plants treated with live SynComs built from microbes enriched in each compartment (SynCom3, SynCom6, SynCom9) and their heat-killed controls (HK-SynCom3, HK-SynCom6, HK-SynCom9). (B) PCA of plants treated with live SynComs assembled from plate-screened *Fusarium*-antagonistic isolates (SynCom12 (S), SynCom12 (R), SynCom12 (L)) and the corresponding heat-killed controls. (C) Volcano plots of differentially expressed genes (DEGs) for each live/heat-killed comparison (log_2_FC ≥ 1, *P* < 0.05). Red and blue dots indicate up- and down-regulated genes, respectively. (D) KEGG pathway enrichment of DEGs in each SynCom treatment. Abbreviations: SynCom3, rhizosphere SynCom from host-enriched genus; SynCom6, root-endosphere SynCom; SynCom9, leaf endosphere SynCom; SynCom12 (S) / SynCom12 (R) / SynCom12 (L), compartment-specific SynComs composed of *F. falciforme* antagonists; HK-, heat-killed counterpart.


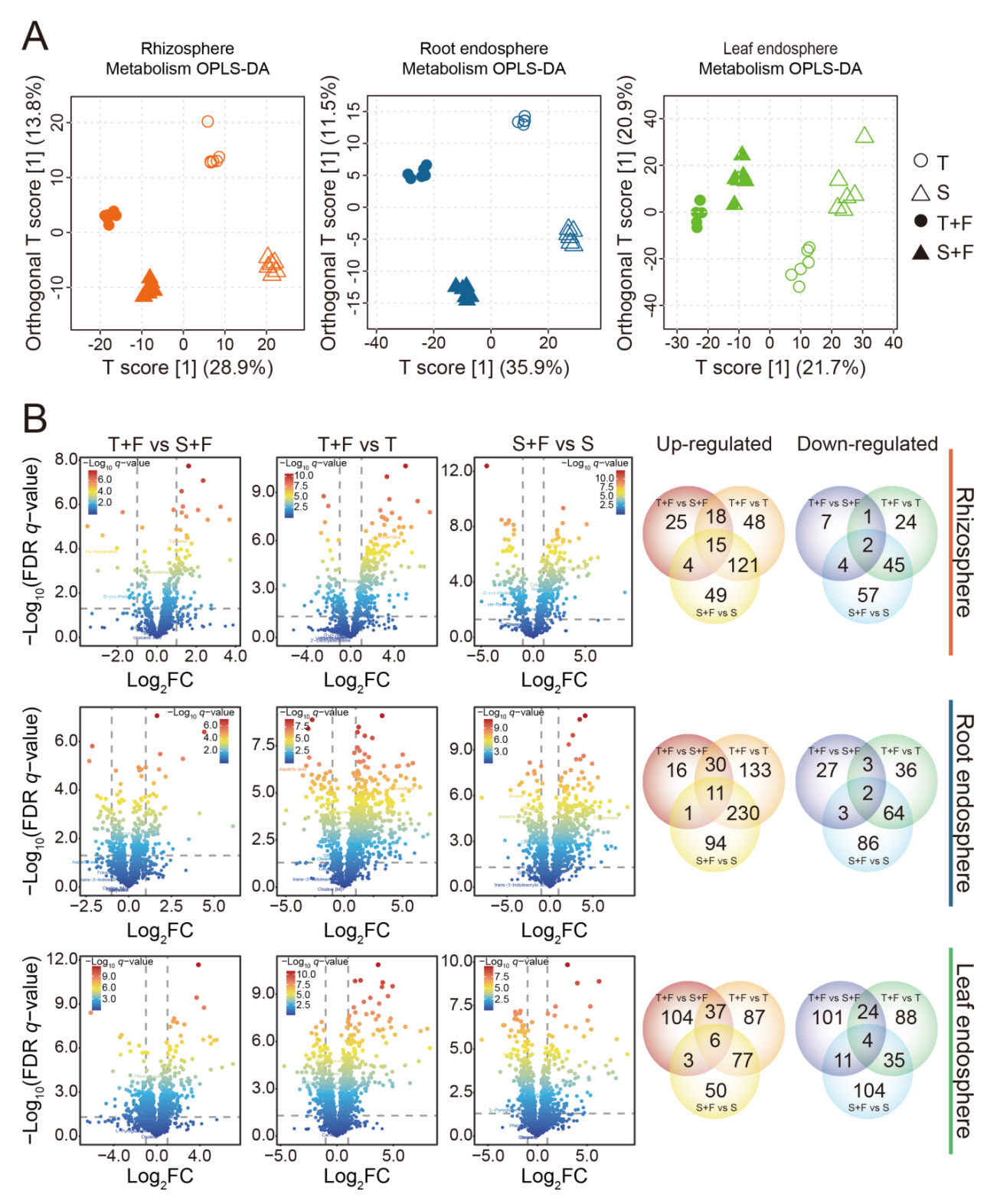


**Fig. S19.** Compartment-specific metabolomic rewiring in tolerant and susceptible soybean after *Fusarium falciforme* challenge. (A) Orthogonal partial-least-squares discriminant analysis (OPLS-DA) of rhizosphere, root endosphere, and leaf endosphere metabolomes from tolerant (GXD2, T) and susceptible (ND12, S) plants with (+F) or without (–F) inoculation. (B) Differential-abundance overview. Left panels: volcano plots of metabolites in each compartment (log_2_FC on the x-axis, –log_₁₀_ FDR on the y-axis); red, enriched, and blue, depleted (FDR < 0.05, |log_2_FC| ≥ 1). Right panel: Venn diagrams summarising overlap of significantly up- and down-regulated metabolites between tolerant (+F vs –F) and susceptible (+F vs –F) genotypes.


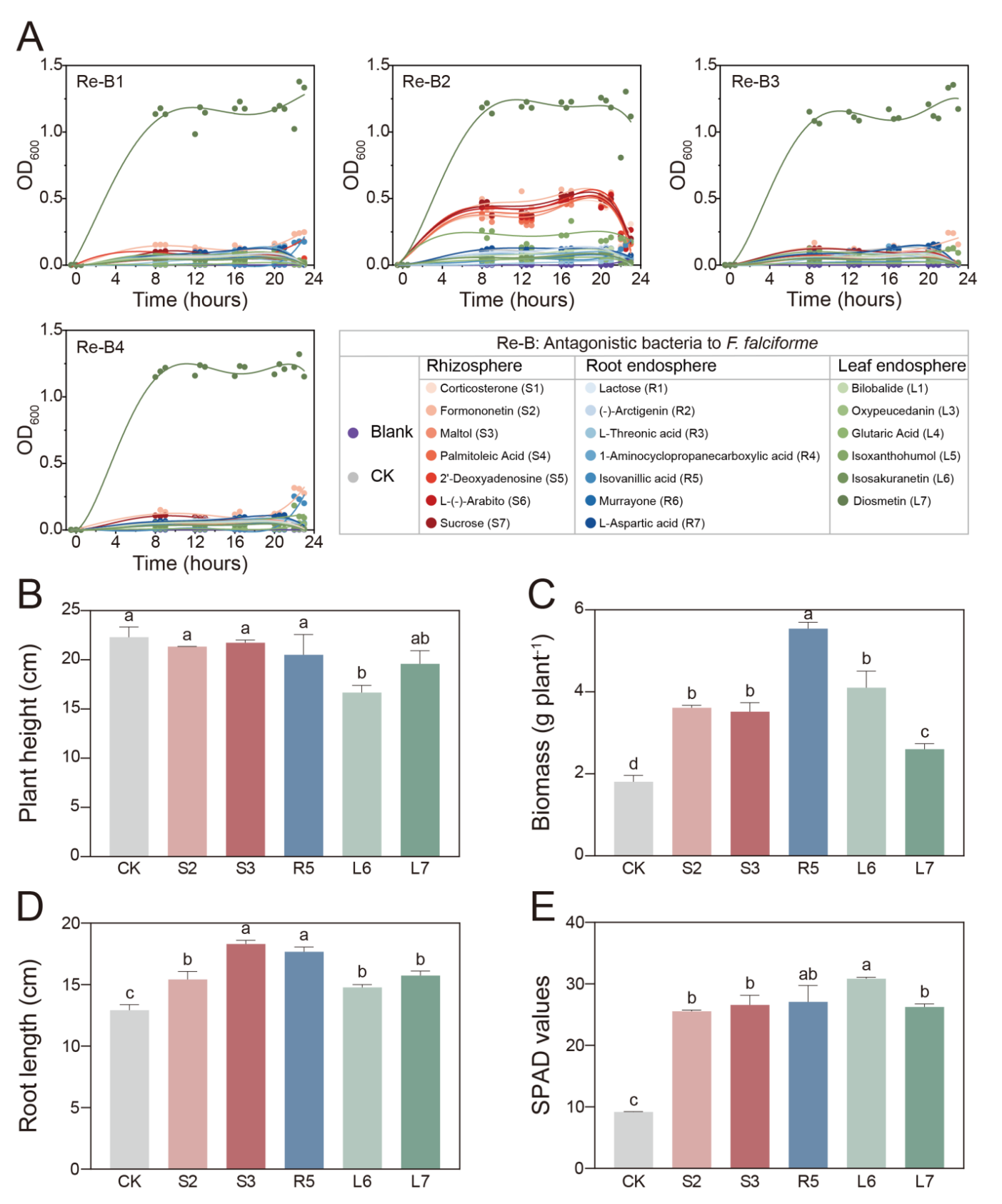


**Fig. S20.** Metabolite-driven stimulation of SynCom bacteria and mitigation of *Fusarium* root-rot symptoms. (A) Growth responses (OD_600_) of the bacterial antagonists used in our bottom-up SynComs when supplied with individual metabolites that accumulate in the rhizosphere, root endosphere, or leaf endosphere after *F. falciforme* infection. Bars show mean ± SD (n = 3); different letters indicate significant differences (one-way ANOVA, *P* < 0.05). (B–E) Performance of the susceptible cultivar 14 days after soil application of the five most stimulatory metabolites (formononetin, maltol, isovanillic acid, isosakuranetin, and diosmetin). Measured traits: (B) plant height, (C) total fresh biomass, (D) primary-root length, and (E) chlorophyll content (SPAD). Columns are mean ± SD (n = 3).


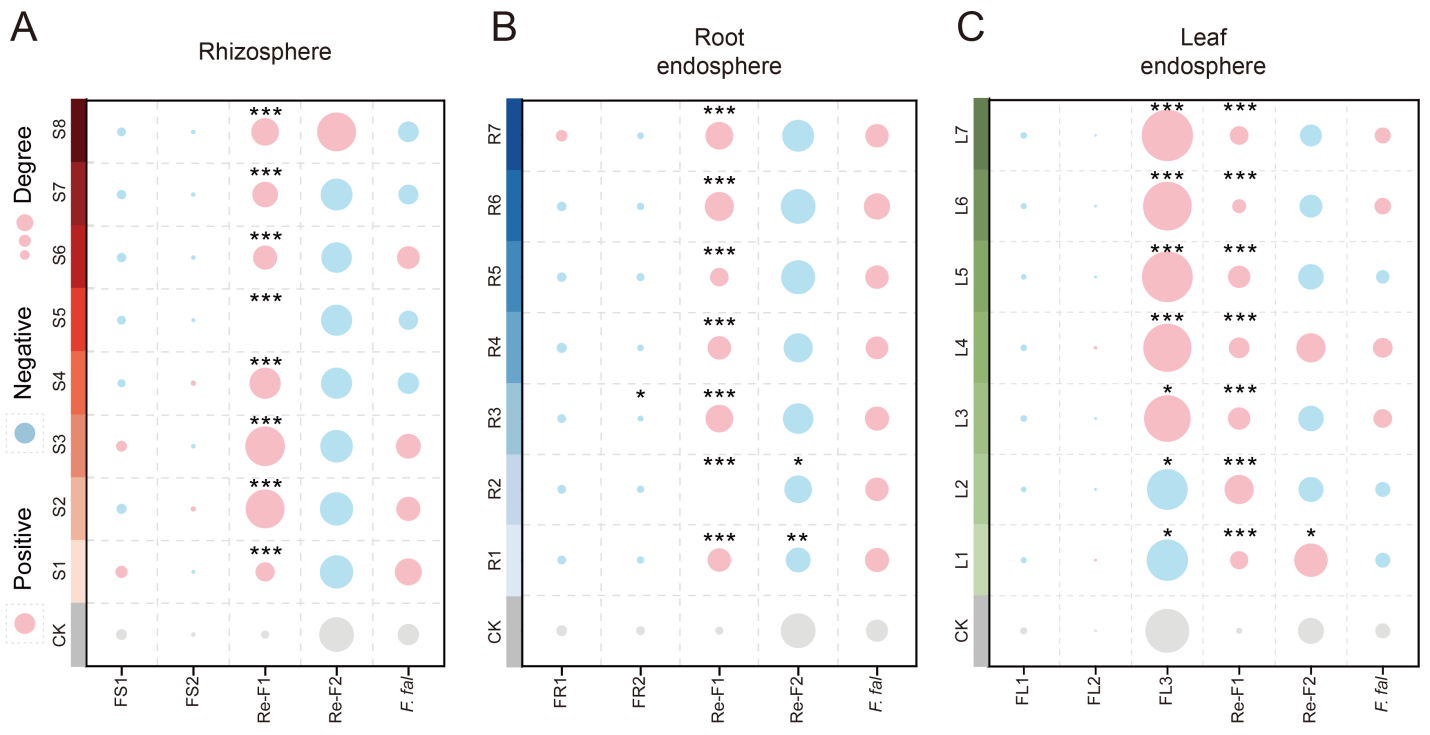


**Fig. S21.** Effects of plant-enriched metabolites on the growth of selected fungal strains. Bubble plots illustrate the mean fungal colony area (n = 3) across various metabolite treatments enriched from the (A) rhizosphere, (B) root endosphere, and (C) leaf endosphere. Data are normalized using log_2_(x+1) transformation. Bubble size corresponds to the log-transformed colony area. Pink and blue bubbles indicate significant growth promotion (positive) and inhibition (negative) relative to the control (CK), respectively. Significant differences were determined by one-way ANOVA followed by Tukey's post-hoc test: *P* < 0.05 (*), *P* < 0.01 (**), *P* < 0.001 (***).

**Supplementary tables**

**Table S1** The medium components used in this study.

| Medium | Component |
| --- | --- |
| NA medium | Peptone 10.0 g, Beef extract 3.0 g, Sodium chloride 5.0 g, Agar 15.0 g, pH 7.2–7.4 |
| PDA medium | 300.0 g Potatoes, Glucose 20.0 g, Agar 15.0 g, Chloramphenicol 0.1 g, pH7.0 |
| Martin medium (modified) | Peptone 5.0 g, Yeast extract 2.0 g, Glucose 20.0 g, K₂HPO₄ 1.0 g, MgSO₄ 0.5 g, pH 6.4 ± 0.2 |

**Table S2** Primers information used in this study.

| Gene fragment | Primers | Region | Sequence (5’-3’) | PCR program | Reference |
| --- | --- | --- | --- | --- | --- |
| 16S rRNA | 338F | V3-V4 | ACTCCTACGGGAGGCAGCAG | 3 min initial denaturation at 95°C, 35 cycles of 30 s at 94°C, 30 s at 54°C, and 45 s at 72°C, with a final 10 min elongation at 72°C. | [3] |
|  | 806R |  | GGACTACHVGGGTWTCTAAT |  |  |
| ITS | ITS1F | ITS1 | CTTGGTCATTTAGAGGAAGTAA | 3 min initial denaturation at 95°C, 35 cycles of 30 s at 94°C, 30 s at 54°C, and 45 s at 72°C, with a final 10 min elongation at 72°C. | [4] |
|  | ITS2R |  | GCTGCGTTCTTCATCGATGC |  |  |
| 16S rRNA | 27F | - | AGAGTTTGATCCTGGCTCAG | 4 min initial denaturation at 94°C, 32 cycles of 30 s at 94°C, 30 s at 55°C, and 50 s at 72°C, with a final 10 min elongation at 72°C. | [5] |
|  | 1492R | - | GGTTACCTTGTTACGACTT |  |  |
| ITS | ITS1 | - | TCCGTAGGTGAACCTGCGG | 4 min initial denaturation at 94°C, 32 cycles of 30 s at 94°C, 30 s at 55°C, and 50 s at 72°C, with a final 10 min elongation at 72°C. | [6] |
|  | ITS4 | - | TCCTCCGCTTATTGATATGC |  |  |
| Fal | Fal-qF | - | GAAGTTGGGTGTTTTACGGC | 4 min initial denaturation at 94°C, 35 cycles of 30 s at 94°C, 30 s at 60°C, and 8 s at 72°C, with a final 10 min elongation at 72°C. | This study |
|  | Fal-qR | - | CCCCAAATACAGTGGCGGTC |  |  |

**Table S3** Scoring criteria for root-rot disease severity index.

| Score | Description of Symptoms |
| --- | --- |
| 0 | No visible disease symptoms. |
| 1 | Slight wilting on cotyledons or true leaves, with normal growth. |
| 2 | Distinct necrotic plaques on leaves; root symptoms appearing; growth delay. |
| 4 | Complete plant wilting; plant death or severely stunted/dwarfed growth. |

**Table S4** PERMANOVA by adonis of all bacterial 16S rRNA and fungal ITS1 samples. PERMANOVA analysis using the Bray Curtis dissimilarities for plant compartment, genotype, and *F.* *falciforme* in beta diversity.

| Microbial communities | Variables | Df^a^ | SumsOfSqs^b^ | MeanSqs^c^ | *F*.Model | *R*^2^ | Pr(> *F*)^d^ |
| --- | --- | --- | --- | --- | --- | --- | --- |
| Bacterial community | Compartment | 2 | 15.06 | 7.53 | 177.8 | 0.74 | 0.001 |
|  | Genotype | 1 | 0.21 | 0.21 | 5.1 | 0.01 | 0.005 |
|  | *F.* *falciforme* | 1 | 0.80 | 0.80 | 18.9 | 0.04 | 0.001 |
| Fungal community | Compartment | 2 | 6.11 | 3.06 | 37.7 | 0.26 | 0.001 |
|  | Genotype | 1 | 1.12 | 1.12 | 13.9 | 0.05 | 0.001 |
|  | *F.* *falciforme* | 1 | 0.94 | 0.94 | 11.6 | 0.04 | 0.001 |

^a^ degrees of freedom, ^b^ sum of squares, ^c^ mean sum of squares, ^d^ *P* values are based on 999 permutations with subsequent Bonferroni correction.

**Table S5** PERMANOVA by adonis of bacterial 16S rRNA conducted separately for each compartment. PERMANOVA analysis using the BrayCurtis dissimilarities for genotype and *F.* *falciforme* inoculation performed separately at individual compartment.

| Compartment | Factor | Df^a^ | SumsOfSqs^b^ | MeanSqs^c^ | *F*.Model | *R*^2^ | Pr(> *F*)^d^ |
| --- | --- | --- | --- | --- | --- | --- | --- |
| Rhizosphere | genotype | 1 | 0.266 | 0.266 | 3.7 | 0.10 | 0.001 |
|  | *F.* *falciforme* | 1 | 0.607 | 0.606 | 8.5 | 0.24 | 0.001 |
| Root endosphere | genotype | 1 | 0.247 | 0.247 | 4.4 | 0.09 | 0.009 |
|  | *F.* *falciforme* | 1 | 1.219 | 1.219 | 21.8 | 0.46 | 0.001 |
| Leaf endosphere | genotype | 1 | < 0.001 | < 0.001 | 3.3 | 0.13 | 0.061 |
|  | *F.* *falciforme* | 1 | < 0.001 | < 0.001 | 1.8 | 0.07 | 0.183 |

^a^ degrees of freedom, ^b^ sum of squares, ^c^ mean sum of squares, ^d^ *P* values are based on 999 permutations with subsequent Bonferroni correction.

**Table S6** PERMANOVA by adonis of fungal ITS1 conducted separately for each compartment. PERMANOVA analysis using the Bray Curtis dissimilarities for genotype and *F.* *falciforme* inoculation performed separately at individual compartment.

| Compartment | Factor | Df^a^ | SumsOfSqs^b^ | MeanSqs^c^ | *F*.Model | *R*^2^ | Pr(> *F*)^d^ |
| --- | --- | --- | --- | --- | --- | --- | --- |
| Rhizosphere | *F.* *falciforme* | 1 | 3.286 | 3.286 | 78.0 | 0.70 | 0.001 |
|  | genotype | 1 | 0.264 | 0.264 | 6.3 | 0.06 | 0.005 |
| Root endosphere | *F.* *falciforme* | 1 | 4.942 | 4.942 | 62.9 | 0.72 | 0.001 |
|  | genotype | 1 | 0.156 | 0.156 | 2.0 | 0.02 | 0.196 |
| Leaf endosphere | *F.* *falciforme* | 1 | 0.680 | 0.800 | 5.5 | 0.11 | 0.012 |
|  | genotype | 1 | 2.943 | 2.943 | 24.0 | 0.47 | 0.001 |

^a^ degrees of freedom, ^b^ sum of squares, ^c^ mean sum of squares, ^d^ *P* values are based on 999 permutations with subsequent Bonferroni correction.

**Table S7** Linear-mixed models (LMMs) for alpha diversity indices. Effects of plant compartment, genotype, and *F.* *falciforme* inoculation on bacterial and fungal community alpha diversity indices were tested with linear-mixed models (LMMs). Significance was assessed using type II ANOVA with Kenward–Rodger approximation of the degrees of freedom in a linear-mixed model.

| Microbial communities | Variables | Shannon index | | Observed ASVs | |
| --- | --- | --- | --- | --- | --- |
|  |  | *F* value | *P* ( > *F*) | *F* value | *P* ( > *F*) |
| Bacterial community | Compartment | 3538.0 | < 0.001 | 876.1 | < 0.001 |
|  | Host genotype | < 0.1 | 0.914 | 6.0 | 0.017 |
|  | *F.* *falciforme* | 1.3 | 0.251 | 10.6 | 0.002 |
| Fungal community | Compartment | 38.0 | < 0.001 | 178.9 | < 0.001 |
|  | Host genotype | 8.5 | 0.005 | 0.7 | 0.404 |
|  | *F.* *falciforme* | 105.7 | < 0.001 | 1.4 | 0.235 |

**Tables S8** Topology properties of the interkingdom networks under *F.* *falciforme*-tolerant (T) and *F.* *falciforme*-sensitive (S) soybean genotypes without and with *F.* *falciforme* (+F) inoculation at individual compartment.

| Compartment | Network metrics | Number of nodes | Number of edges | Average degree (avgK) | Average weight degree | Modularity (M) |
| --- | --- | --- | --- | --- | --- | --- |
| Rhizosphere | T | 260 | 2610 | 20.077 | 4.588 | 2.661 |
|  |  | （B:189; F:71） | (P:61.8%; N:38.2%) |  |  |  |
|  | T+F | 211 | 1715 | 16.256 | 5.497 | 1.600 |
|  |  | (B:180; F:31) | （P:67.9%; N:32.1%） |  |  |  |
|  | S | 241 | 2072 | 17.195 | 2.282 | 19.409 |
|  |  | （B:187; F:54） | （P:56.9%; N:43.1%） |  |  |  |
|  | S+F | 227 | 1923 | 16.943 | 3.391 | 4.196 |
|  |  | （B:185; F:42） | （P:60.3%; N:39.7%） |  |  |  |
| Root endosphere | T | 81 | 359 | 8.864 | 4.054 | 1.581 |
|  |  | （B:64; F:17） | （P:73.0%; N:27.0%） |  |  |  |
|  | T+F | 68 | 246 | 7.235 | 2.950 | 2.327 |
|  |  | （B:66; F:2） | （P:69.5%; N:30.5%） |  |  |  |
|  | S | 83 | 373 | 8.988 | 4.294 | 1.182 |
|  |  | （B:63; F:20） | （P:72.9%; N:27.1%） |  |  |  |
|  | S+F | 72 | 336 | 9.333 | 5.957 | 0.805 |
|  |  | （B:71; F:1） | （P:82.1%; N:17.9%） |  |  |  |
| Leaf endosphere | T | 17 | 30 | 3.529 | 2.187 | -1.136 |
|  |  | （B:2; F:15） | （P:83.3%; N:16.7%） |  |  |  |
|  | T+F | 11 | 22 | 4.000 | 1.797 | -13.088 |
|  |  | （B:2; F:9） | （P:72.7%; N:27.3%） |  |  |  |
|  | S | 30 | 61 | 4.067 | 3.255 | 0.757 |
|  |  | （B:2; F:28） | （P:93.4%; N:6.6%） |  |  |  |
|  | S+F | 19 | 32 | 3.368 | 2.543 | 0.841 |
|  |  | （B:2; F:17） | （P:90.6%; N:9.4%） |  |  |  |

"B" represents bacteria, "F" represents fungi, "P" represents positive, and "N" represents negative.

**Tables S9** Network key nodes of the interkingdom networks *F.* *falciforme*-tolerant (T) and *F.* *falciforme*-sensitive (S) soybean genotypes without and with *F.* *falciforme* (+F) inoculation at individual compartment.

| Compartment | Network metrics | ASVs | Degree | Closeness Centrality | Betweeness Centrality | Geuns |
| --- | --- | --- | --- | --- | --- | --- |
| Rhizosphere | T | BASV353 | 16 | 0.356 | 458.529 | Unclassified *Micropepsaceae* |
|  |  | BASV279 | 20 | 0.338 | 202.168 | *Sphingomonas* |
|  |  | BASV276 | 17 | 0.358 | 229.736 | *Haliangium* |
|  |  | FASV21 | 20 | 0.371 | 361.114 | *Fibulochlamys* |
|  |  | FASV3 | 22 | 0.370 | 284.494 | *Chaetomium* |
|  | T+F | BASV314 | 17 | 0.360 | 345.708 | *Bacillus* |
|  |  | BASV306 | 16 | 0.361 | 280.592 | *Ramlibacter* |
|  |  | BASV115 | 13 | 0.354 | 246.751 | *Streptomyces* |
|  |  | FASV17 | 16 | 0.351 | 241.113 | *Penicillium* |
|  |  | FASV14 | 16 | 0.351 | 241.113 | Unclassified *Aspergillaceae* |
|  | S | BASV55 | 32 | 0.377 | 588.494 | *Mucilaginibacter* |
|  |  | BASV305 | 30 | 0.358 | 372.057 | *Sphingomonas* |
|  |  | BASV2904 | 29 | 0.369 | 594.454 | *Cupriavidus* |
|  |  | FASV1 | 32 | 0.368 | 386.693 | *Neocosmospora* |
|  |  | FASV2 | 22 | 0.363 | 344.706 | *Mortierella* |
|  | S+F | BASV302 | 15 | 0.339 | 322.800 | *Streptomyces* |
|  |  | BASV162 | 16 | 0.321 | 254.123 | *Bacillus* |
|  |  | BASV34 | 17 | 0.356 | 220.134 | *Arthrobacter* |
|  |  | FASV7 | 22 | 0.364 | 298.829 | *Penicillium* |
|  |  | FASV11 | 17 | 0.356 | 220.134 | *Mortierella* |

**Continued Tables S9** Network key nodes of the interkingdom networks *F.* *falciforme*-tolerant (T) and *F.* *falciforme*-sensitive (S) soybean genotypes without and with *F.* *falciforme* (+F) inoculation at individual compartment.

| Compartment | Network metrics | ASVs | Degree | Closeness Centrality | Betweeness Centrality | Geuns |
| --- | --- | --- | --- | --- | --- | --- |
| Root endosphere | T | BASV1341 | 16 | 0.361 | 229.763 | *Burkholderia* |
|  |  | BASV100 | 14 | 0.348 | 230.196 | *Mucilaginibacter* |
|  |  | BASV262 | 12 | 0.319 | 212.621 | *Bradyrhizobium* |
|  |  | FASV7 | 13 | 0.325 | 303.276 | *Penicillium* |
|  |  | FASV81 | 14 | 0.353 | 180.251 | Unclassified |
|  | T+F | BASV178 | 12 | 0.354 | 172.255 | *Dyadobacter* |
|  |  | BASV3 | 11 | 0.303 | 301.896 | Unclassified *Mitochondria* |
|  |  | BASV273 | 10 | 0.300 | 110.419 | *Massilia* |
|  |  | BASV58 | 10 | 0.300 | 152.424 | *Sphingomonas* |
|  |  | FASV1 | 12 | 0.354 | 172.255 | *Neocosmospora* |
|  | S | BASV51 | 17 | 0.382 | 380.688 | *Streptomyces* |
|  |  | BASV135 | 17 | 0.348 | 336.239 | *Sphingomonas* |
|  |  | BASV47 | 16 | 0.384 | 258.824 | *Mesorhizobium* |
|  |  | BASV30 | 14 | 0.354 | 205.585 | *Flavobacterium* |
|  |  | BASV50 | 18 | 0.367 | 170.145 | *Duganella* |
|  | S+F | BASV73 | 17 | 0.396 | 296.486 | *Bosea* |
|  |  | BASV1146 | 15 | 0.411 | 236.078 | *Novosphingobium* |
|  |  | BASV97 | 20 | 0.399 | 151.410 | *Allorhizobium* |
|  |  | BASV106 | 10 | 0.369 | 193.041 | *Caulobacter* |
|  |  | BASV58 | 11 | 0.353 | 105.270 | *Sphingomonas* |

**Continued Tables S9** Network key nodes of the interkingdom networks *F.* *falciforme*-tolerant (T) and *F.* *falciforme*-sensitive (S) soybean genotypes without and with *F.* *falciforme* (+F) inoculation at individual compartment.

| Compartment | Network metrics | ASVs | Degree | Closeness Centrality | Betweeness Centrality | Geuns |
| --- | --- | --- | --- | --- | --- | --- |
| Leaf endosphere | T | FASV468 | 5 | 0.800 | 3.000 | Unclassified *Cladosporiaceae* |
|  |  | FASV29 | 5 | 0.800 | 3.000 | *Cladosporium* |
|  |  | FASV470 | 5 | 1.000 | 0.500 | Unclassified *Rozellomycota* |
|  |  | FASV597 | 5 | 1.000 | 0.500 | *Saccharomyces* |
|  | T+F | FASV29 | 5 | 0.714 | 4.000 | *Cladosporium* |
|  |  | FASV468 | 6 | 0.833 | 2.000 | Unclassified *Cladosporiaceae* |
|  |  | FASV264 | 6 | 0.833 | 2.000 | *Erysiphe* |
|  |  | BASV1 | 1 | 1.000 | 1.000 | Unclassified *Chloroplast* |
|  | S | FASV279 | 6 | 0.484 | 0.153 | *Talaromyces* |
|  |  | FASV97 | 6 | 0.429 | 0.086 | *Cercospora* |
|  |  | FASV266 | 6 | 0.349 | 0.048 | *Apiotrichum* |
|  |  | FASV493 | 6 | 0.349 | 0.043 | *Cladosporium* |
|  |  | FASV552 | 6 | 1.000 | 0.009 | *Cladosporium* |
|  | S+F | FASV474 | 6 | 0.636 | 14.000 | *Cladosporium* |
|  |  | FASV29 | 5 | 0.583 | 10.000 | *Cladosporium* |
|  |  | FASV266 | 4 | 0.467 | 6.000 | *Apiotrichum* |
|  |  | FASV489 | 4 | 0.438 | 6.000 | *Cladosporium* |

"B" represents bacteria, and "F" represents fungi.

**Tables S10** Differences between inoculated and non-inoculated *F.* *falciforme* ASVs.

| Plant compartment | ASVs ID | Taxonomic position | *P*1 value  (log_2_FC ≥ 1) | *P*2 value  (log_2_FC ≥ 1) |
| --- | --- | --- | --- | --- |
| Rhizosphere | ASV283 | *Bacteria*-*Firmicutes*-*Bacilli*-*Brevibacillales*-*Brevibacillaceae*-*Brevibacillus*-unclassified | 0.017 | 0.042 |
|  | ASV2491 | *Bacteria*-*Firmicutes*-*Bacilli*-*Bacillales*-*Bacillaceae*-*Bacillus*-unclassified | 0.032 | 0.232 |
|  | ASV329 | *Bacteria*-*Firmicutes*-*Bacilli*-*Bacillales*-*Bacillaceae*-*Bacillus*-unclassified | 0.010 | - |
|  | ASV31 | *Bacteria*-*Proteobacteria*-*Alphaproteobacteria*-*Rhizobiales*-*Beijerinckiaceae*-*Bosea*-unclassified | < 0.001 | 0.057 |
|  | ASV907 | *Bacteria*-*Firmicutes*-*Bacilli*-*Paenibacillales*-*Paenibacillaceae*-*Thermobacillus*-unclassified | < 0.001 | < 0.001 |
|  | ASV538 | *Bacteria*-*Firmicutes*-*Bacilli*-*Paenibacillales*-*Paenibacillaceae*-*Paenibacillus*-*Paenibacillus_cookii* | 0.023 | - |
|  | ASV668 | *Bacteria*-*Firmicutes*-*Bacilli*-*Bacillales*-*Bacillaceae*-*Bacillus*-*Bacillus_funiculus* | 0.048 | 0.024 |
|  | ASV440 | *Bacteria*-*Actinobacteriota*-*Actinobacteria*-*Micrococcales*-*Micrococcaceae*-unclassified | 0.001 | < 0.001 |
|  | ASV362 | *Bacteria*-*Firmicutes*-*Bacilli*-*Bacillales*-*Bacillaceae*-*Bacillus*-unclassified | 0.022 | - |
|  | ASV300 | *Bacteria*-*Firmicutes*-*Clostridia*-*Lachnospirales*-*Lachnospiraceae*-uncultured | 0.028 | 0.005 |
|  | ASV468 | *Bacteria*-*Proteobacteria*-*Alphaproteobacteria*-*Rhizobiales*-*Xanthobacteraceae*-unclassified | 0.026 | 0.001 |
|  | ASV355 | *Bacteria*-*Firmicutes*-*Clostridia*-*Clostridiales*-*Clostridiaceae*-*Clostridium_sensu_stricto_1*-unclassified | 0.001 | < 0.001 |
|  | ASV234 | *Bacteria*-*Actinobacteriota*-*Actinobacteria*-*Micrococcales*-*Intrasporangiaceae*-unclassified_f__*Intrasporangiaceae* | 0.010 | 0.011 |
|  | ASV656 | *Bacteria*-*Firmicutes*-*Clostridia*-norank_c__*Clostridia*-*Hungateiclostridiaceae*-*Pseudoclostridium*-*Pseudoclostridium_thermosuccinogenes* | 0.042 | 0.004 |
|  | ASV443 | *Bacteria*-*Firmicutes*-*Bacilli*-*Bacillales*-*Sporolactobacillaceae*-*Tuberibacillus*-*Tuberibacillus_calidus* | 0.046 | 0.095 |
|  | ASV434 | *Bacteria*-*Proteobacteria*-*Gammaproteobacteria*-*Burkholderiales*-*Burkholderiaceae*-*Burkholderia*-*Caballeronia*-*Paraburkholderia*-unclassified | 0.010 | 0.005 |

**Continued Tables S10** Differences between inoculated and non-inoculated *F.* *falciforme* ASVs.

| Plant compartment | ASVs ID | Taxonomic position | *P*1 value  (log_2_FC ≥ 1) | *P*2 value  (log_2_FC ≥ 1) |
| --- | --- | --- | --- | --- |
| Rhizosphere | ASV189 | *Bacteria*-*Actinobacteriota*-*Actinobacteria*-*Micrococcales*-*Micrococcaceae*-*Sinomonas*-unclassified | 0.015 | 0.609 |
|  | ASV438 | *Bacteria*-*Actinobacteriota*-*Actinobacteria*-*Corynebacteriales*-*Nocardiaceae*-*Nocardia*-*Nocardia_nova* | 0.028 | 0.011 |
|  | ASV495 | *Bacteria*-*Firmicutes*-*Bacilli*-*Bacillales*-*Bacillaceae*-*Bacillus*-*Bacillus_asahii* | 0.037 | 0.054 |
|  | ASV392 | *Bacteria*-*Firmicutes*-*Bacilli*-*Brevibacillales*-*Brevibacillaceae*-*Brevibacillus*-*Brevibacillus_fulvus* | < 0.001 | 0.001 |
|  | ASV366 | *Bacteria*-*Firmicutes*-*Bacilli*-*Bacillales*-*Bacillaceae*-*Geobacillus*-unclassified | < 0.001 | 0.004 |
|  | ASV291 | *Bacteria*-*Actinobacteriota*-*Actinobacteria*-*Micrococcales*-*Micrococcaceae*-unclassified | < 0.001 | 0.103 |
|  | ASV261 | *Bacteria*-*Actinobacteriota*-*Actinobacteria*-*Micrococcales*-*Micrococcaceae*-unclassified | 0.006 | < 0.001 |
|  | ASV486 | *Bacteria*-*Proteobacteria*-*Alphaproteobacteria*-*Caulobacterales*-*Caulobacteraceae*-unclassified | < 0.001 | 0.003 |
|  | ASV83 | *Bacteria*-*Actinobacteriota*-*Actinobacteria*-*Micrococcales*-*Microbacteriaceae*-*Leifsonia*-unclassified | < 0.001 | 0.109 |
|  | ASV750 | *Bacteria*-*Bacteroidota*-*Bacteroidia*-*Cytophagales*-*Spirosomaceae*-*Arcicella*-unclassified | 0.009 | 0.341 |
|  | ASV874 | *Bacteria*-*Firmicutes*-*Bacilli*-*Paenibacillales*-*Paenibacillaceae*-*Thermobacillus*-uncultured | < 0.001 | 0.288 |
|  | ASV163 | *Bacteria*-*Bacteroidota*-*Bacteroidia*-*Sphingobacteriales*-*Sphingobacteriaceae*-*Mucilaginibacter*-unclassified | 0.037 | < 0.001 |
|  | ASV487 | *Bacteria*-*Firmicutes*-*Bacilli*-*Bacillales*-*Bacillaceae*-*Bacillus*-unclassified | < 0.001 | 0.002 |
|  | ASV344 | *Bacteria*-*Firmicutes*-*Bacilli*-*Bacillales*-*Planococcaceae*-*Lysinibacillus*-unclassified | < 0.001 | < 0.001 |
|  | ASV785 | *Bacteria*-*Bacteroidota*-*Bacteroidia*-*Flavobacteriales*-*Flavobacteriaceae*-*Flavobacterium*-unclassified | 0.009 | - |
|  | ASV289 | *Bacteria*-*Proteobacteria*-*Gammaproteobacteria*-*Burkholderiales*-*Oxalobacteraceae*-*Massilia*-unclassified | 0.001 | 0.211 |
|  | ASV439 | *Bacteria*-*Firmicutes*-*Bacilli*-*Bacillales*-*Bacillaceae*-*Geobacillus*-unclassified | 0.001 | 0.062 |

**Continued Tables S10** Differences between inoculated and non-inoculated *F.* *falciforme* ASVs.

| Plant compartment | ASVs ID | Taxonomic position | *P*1 value  (log_2_FC ≥ 1) | *P*2 value  (log_2_FC ≥ 1) |
| --- | --- | --- | --- | --- |
| Rhizosphere | ASV208 | *Bacteria*-*Cyanobacteria*-*Sericytochromatia*-uncultured | 0.004 | 0.163 |
|  | ASV471 | *Bacteria*-*Proteobacteria*-*Gammaproteobacteria*-*Burkholderiales*-*Nitrosomonadaceae*-*Ellin6067*-unclassified | 0.044 | 0.074 |
|  | ASV567 | *Bacteria*-*Firmicutes*-*Symbiobacteriia*-*Symbiobacteriales*-*Symbiobacteraceae*-*Symbiobacterium*-uncultured | 0.037 | 0.029 |
|  | ASV788 | *Bacteria*-*Actinobacteriota*-*Actinobacteria*-*Streptosporangiales*-*Thermomonosporaceae*-*Thermobispora*-*Thermobispora*_*bispora* | 0.005 | < 0.001 |
|  | ASV314 | *Bacteria*-*Firmicutes*-*Bacilli*-*Bacillales*-*Bacillaceae*-*Bacillus*-unclassified | 0.003 | < 0.001 |
|  | ASV655 | *Bacteria*-*Actinobacteriota*-*Actinobacteria*-*Streptosporangiales*-*Thermomonosporaceae*-*Thermobispora*-unclassified_g__*Thermobispora* | 0.035 | 0.031 |
|  | ASV295 | *Bacteria*-*Actinobacteriota*-*Actinobacteria*-*Micromonosporales*-*Micromonosporaceae*-unclassified | 0.009 | < 0.001 |
|  | ASV719 | *Bacteria*-*Firmicutes*-*Bacilli*-*Paenibacillales*-*Paenibacillaceae*-*Thermobacillus*-uncultured | 0.002 | < 0.001 |
|  | ASV257 | *Bacteria*-*Firmicutes*-*Bacilli*-*Bacillales*-*Bacillaceae*-*Bacillus*-unclassified | 0.003 | 0.005 |
|  | ASV378 | *Bacteria*-*Actinobacteriota*-*Actinobacteria*-*Micrococcales*-*Intrasporangiaceae*-unclassified | < 0.001 | - |
|  | ASV259 | *Bacteria*-*Firmicutes*-*Bacilli*-*Bacillales*-*Bacillaceae*-*Bacillus*-unclassified | < 0.001 | 0.851 |
|  | ASV258 | *Bacteria*-*Firmicutes*-*Bacilli*-*Bacillales*-*Bacillaceae*-*Bacillus*-unclassified | 0.001 | 0.203 |
|  | ASV14 | *Bacteria*-*Proteobacteria*-*Alphaproteobacteria*-*Sphingomonadales*-*Sphingomonadaceae*-*Novosphingobium*-unclassified | < 0.001 | 0.305 |
|  | ASV270 | *Bacteria*-*Actinobacteriota*-*Actinobacteria*-*Micrococcales*-*Micrococcaceae*-*Sinomonas*-unclassified | 0.006 | 0.003 |
|  | ASV2206 | *Bacteria*-*Acidobacteriota*-*Vicinamibacteria*-*Vicinamibacterales*-unclassified | 0.044 | - |
|  | ASV459 | *Bacteria*-*Firmicutes*-*Bacilli*-*Paenibacillales*-*Paenibacillaceae*-*Paenibacillus*-unclassified | 0.004 | 0.014 |
|  | ASV311 | *Bacteria*-*Firmicutes*-*Bacilli*-*Brevibacillales*-*Brevibacillaceae*-*Brevibacillus*-unclassified | 0.005 | 0.004 |

**Continued Tables S10** Differences between inoculated and non-inoculated *F.* *falciforme* ASVs.

| Plant compartment | ASVs ID | Taxonomic position | *P*1 value  (log_2_FC ≥ 1) | *P*2 value  (log_2_FC ≥ 1) |
| --- | --- | --- | --- | --- |
| Rhizosphere | ASV1102 | *Bacteria*-*Proteobacteria*-*Gammaproteobacteria*-*Burkholderiales*-*SC-I-84*-norank_f__*SC-I-84*-unclassified | 0.049 | 0.010 |
|  | ASV335 | *Bacteria*-*Firmicutes*-*Clostridia*-*Clostridiales*-*Clostridiaceae*-*Clostridium_sensu_stricto_8*-unclassified | 0.020 | 0.001 |
|  | ASV499 | *Bacteria*-*Proteobacteria*-*Gammaproteobacteria*-*Burkholderiales*-*Comamonadaceae*-*Ramlibacter*-unclassified | 0.002 | 0.708 |
|  | ASV626 | *Bacteria*-*Firmicutes*-*Clostridia*-*Clostridiales*-*Caloramatoraceae*-*Fonticella*-unclassified | 0.018 | 0.024 |
|  | ASV536 | *Bacteria*-*Firmicutes*-*Bacilli*-*Paenibacillales*-*Paenibacillaceae*-*Thermobacillus*-uncultured | 0.027 | 0.035 |
|  | ASV898 | *Bacteria*-*Chloroflexi*-*Ktedonobacteria*-*Ktedonobacterales*--uncultured | 0.033 | - |
|  | ASV96 | *Bacteria*-*Firmicutes*-*Bacilli*-*Bacillales*-*Bacillaceae*-*Bacillus*-unclassified | 0.001 | 0.817 |
|  | ASV600 | *Bacteria*-*Firmicutes*-*Bacilli*-*Bacillales*-*Bacillaceae*-*Bacillus*-unclassified | < 0.001 | 0.030 |
|  | ASV602 | *Bacteria*-*Myxococcota*-*Polyangia*-*Haliangiales*-*Haliangiaceae*-*Haliangium*-unclassified | 0.048 | 0.002 |
|  | ASV98 | *Bacteria*-*Proteobacteria*-*Alphaproteobacteria*-*Caulobacterales*-*Caulobacteraceae*-*Phenylobacterium*-unclassified | 0.002 | 0.012 |
|  | ASV43 | *Bacteria*-*Proteobacteria*-*Gammaproteobacteria*-*Burkholderiales*-*Oxalobacteraceae*-*Massilia*-unclassified | 0.014 | 0.002 |
|  | ASV515 | *Bacteria*-*Firmicutes*-*Symbiobacteriia*-*Symbiobacteriales*-*Symbiobacteraceae*-*Symbiobacterium*-*Symbiobacterium_thermophilum_IAM_14863* | 0.015 | 0.002 |
|  | ASV977 | *Bacteria*-*Bacteroidota*-*Bacteroidia*-*Chitinophagales*-*Chitinophagaceae*-*Segetibacter*-unclassified | 0.004 | 0.925 |
|  | ASV280 | *Bacteria*-*Actinobacteriota*-*Actinobacteria*-*Micrococcales*-*Micrococcaceae*-*Sinomonas*-unclassified | < 0.001 | 0.002 |
|  | ASV247 | *Bacteria*-*Gemmatimonadota*-*Gemmatimonadetes*-*Gemmatimonadales*-*Gemmatimonadaceae*-*Roseisolibacter*-unclassified | < 0.001 | 0.001 |
|  | ASV1451 | *Bacteria*-*Firmicutes*-*Bacilli*-*Paenibacillales*-*Paenibacillaceae*-*Paenibacillus*-unclassified | 0.039 | 0.004 |

**Continued Tables S10** Differences between inoculated and non-inoculated *F.* *falciforme* ASVs.

| Plant compartment | ASVs ID | Taxonomic position | *P*1 value  (log_2_FC ≥ 1) | *P*2 value  (log_2_FC ≥ 1) |
| --- | --- | --- | --- | --- |
| Rhizosphere | ASV184 | *Bacteria*-*Actinobacteriota*-*Actinobacteria*-*Micrococcales*-*Intrasporangiaceae*-*Terrabacter*-unclassified | < 0.001 | 0.011 |
|  | ASV42 | *Bacteria*-*Actinobacteriota*-*Actinobacteria*-*Micrococcales*-*Micrococcaceae*-*Arthrobacter*-unclassified | 0.001 | 0.205 |
|  | ASV1871 | *Bacteria*-*Firmicutes*-*Limnochordia*-*Limnochordales*-uncultured | 0.039 | 0.514 |
|  | ASV32 | *Bacteria*-*Proteobacteria*-*Gammaproteobacteria*-*Burkholderiales*-*Comamonadaceae*-unclassified | < 0.001 | 0.141 |
|  | ASV162 | *Bacteria*-*Firmicutes*-*Bacilli*-*Bacillales*-*Bacillaceae*-*Bacillus*-unclassified | 0.001 | 0.007 |
|  | ASV746 | *Bacteria*-*Myxococcota*-*Polyangia*-*Haliangiales*-*Haliangiaceae*-*Haliangium*-unclassified | 0.011 | 0.915 |
|  | ASV911 | *Bacteria*-*Proteobacteria*-*Gammaproteobacteria*-*Burkholderiales*-*Nitrosomonadaceae*-*mle1-7*-unclassified | 0.007 | 0.083 |
|  | ASV304 | *Bacteria*-*Firmicutes*-*Bacilli*-*Paenibacillales*-*Paenibacillaceae*-*Ammoniphilus*-unclassified | < 0.001 | < 0.001 |
|  | ASV717 | *Bacteria*-*Firmicutes*-*Bacilli*-*Paenibacillales*-*Paenibacillaceae*-*Paenibacillus*-uncultured | 0.019 | 0.001 |
|  | ASV266 | *Bacteria*-*Firmicutes*-*Bacilli*-*Bacillales*-*Bacillaceae*-*Bacillus*-unclassified | 0.001 | 0.317 |
|  | ASV260 | *Bacteria*-*Firmicutes*-*Bacilli*-*Bacillales*-*Bacillaceae*-*Bacillus*-unclassified | 0.003 | 0.007 |
|  | ASV2830 | *Bacteria*-*Firmicutes*-*Bacilli*-*Thermoactinomycetales*-*Thermoactinomycetaceae*-uncultured | 0.039 | 0.522 |
|  | ASV726 | *Bacteria*-*Firmicutes*-*Bacilli*-*Thermoactinomycetales*-*Thermoactinomycetaceae*-*Planifilum*-unclassified | 0.012 | 0.571 |
|  | ASV301 | *Bacteria*-*Firmicutes*-*Bacilli*-*Bacillales*-*Bacillaceae*-*Bacillus*-unclassified | < 0.001 | 0.002 |
|  | ASV557 | *Bacteria*-*Actinobacteriota*-*Actinobacteria*-*Frankiales*-*Acidothermaceae*-*Acidothermus*-unclassified | 0.015 | 0.005 |
|  | ASV1 | *Fungi*-*Ascomycota*-*Sordariomycetes*-*Hypocreales*-*Nectriaceae*-*Neocosmospora*-*Neocosmospora_rubicola* | < 0.001 | < 0.001 |

**Continued Tables S10** Differences between inoculated and non-inoculated *F.* *falciforme* ASVs.

| Plant compartment | ASVs ID | Taxonomic position | *P*1 value  (log_2_FC ≥ 1) | *P*2 value  (log_2_FC ≥ 1) |
| --- | --- | --- | --- | --- |
| Root endosphere | ASV178 | *Bacteria*-*Bacteroidota*-*Bacteroidia*-*Cytophagales*-*Spirosomaceae*-*Dyadobacter*-unclassified | 0.027 | 0.313 |
|  | ASV144 | *Bacteria*-*Proteobacteria*-*Gammaproteobacteria*-*Burkholderiales*-*Comamonadaceae*-*Roseateles*-unclassified | 0.025 | 0.627 |
|  | ASV223 | *Bacteria*-*Proteobacteria*-*Alphaproteobacteria*-*Rhizobiales*-*Devosiaceae*-*Devosia*-unclassified | < 0.001 | 0.003 |
|  | ASV2234 | *Bacteria*-*Proteobacteria*-*Alphaproteobacteria*-*Rhizobiales*-*Rhizobiaceae*-unclassified | 0.047 | 0.006 |
|  | ASV190 | *Bacteria*-*Proteobacteria*-*Alphaproteobacteria*-*Rhizobiales*-*Beijerinckiaceae-28-YEA-48-Afipia_genosp._6* | 0.001 | 0.361 |
|  | ASV1163 | *Bacteria*-*Proteobacteria*-*Alphaproteobacteria*-*Rhizobiales*-*Rhizobiaceae*-*Allorhizobium*-*Neorhizobium*-*Pararhizobium*-*Rhizobium*-unclassified | 0.002 | 0.150 |
|  | ASV1139 | *Bacteria*-*Proteobacteria*-*Alphaproteobacteria*-*Caulobacterales*-*Caulobacteraceae*-*Phenylobacterium*-unclassified | 0.049 | 0.041 |
|  | ASV438 | *Bacteria*-*Actinobacteriota*-*Actinobacteria*-*Corynebacteriales*-*Nocardiaceae*-*Nocardia*-*Nocardia_nova* | < 0.001 | < 0.001 |
|  | ASV180 | *Bacteria*-*Proteobacteria*-*Alphaproteobacteria*-*Rhizobiales*-*Rhizobiaceae*-unclassified | 0.006 | 0.005 |
|  | ASV1142 | *Bacteria*-*Proteobacteria*-*Alphaproteobacteria*-*Rhizobiales*-*Rhizobiaceae*-*Mesorhizobium*-unclassified | 0.024 | 0.033 |
|  | ASV1135 | *Bacteria*-*Proteobacteria*-*Alphaproteobacteria*-*Sphingomonadales*-*Sphingomonadaceae*-*Novosphingobium*-unclassified | < 0.001 | 0.165 |
|  | ASV182 | *Bacteria*-*Bacteroidota*-*Bacteroidia*-*Cytophagales*-*Spirosomaceae*-*Spirosoma*-*Spirosoma_gilvum* | 0.002 | 0.757 |
|  | ASV1141 | *Bacteria*-*Proteobacteria*-*Alphaproteobacteria*-*Reyranellales*-*Reyranellaceae*-*Reyranella*-unclassified | 0.011 | 0.007 |
|  | ASV1133 | *Bacteria*-*Proteobacteria*-*Gammaproteobacteria*-*Gammaproteobacteria_Incertae_Sedis*-unclassified | 0.004 | 0.004 |
|  | ASV1138 | *Bacteria*-*Proteobacteria*-*Alphaproteobacteria*-*Rhizobiales*-*Rhizobiaceae*-unclassified_f__*Rhizobiaceae* | 0.011 | 0.058 |

**Continued Tables S10** Differences between inoculated and non-inoculated *F.* *falciforme* ASVs.

| Plant compartment | ASVs ID | Taxonomic position | *P*1 value  (log_2_FC ≥ 1) | *P*2 value  (log_2_FC ≥ 1) |
| --- | --- | --- | --- | --- |
| Root endosphere | ASV1173 | *Bacteria*-*Bdellovibrionota*-*Oligoflexia*-*0319-6G20*-uncultured | 0.024 | 0.002 |
|  | ASV53 | *Bacteria*-*Proteobacteria*-*Alphaproteobacteria*-*Caulobacterales*-*Caulobacteraceae*-*Caulobacter*-unclassified | 0.001 | 0.693 |
|  | ASV629 | *Bacteria*-*Actinobacteriota*-*Actinobacteria*-*Corynebacteriales*-*Mycobacteriaceae*-*Mycobacterium*-unclassified | < 0.001 | < 0.001 |
|  | ASV56 | *Bacteria*-*Proteobacteria*-*Alphaproteobacteria*-*Sphingomonadales*-*Sphingomonadaceae*-*Novosphingobium*-unclassified | < 0.001 | 0.159 |
|  | ASV1015 | *Bacteria*-*Bacteroidota*-*Bacteroidia*-*Chitinophagales*-*Chitinophagaceae*-norank_f__*Chitinophagaceae*-*Chitinophaga*_sp._*WW12011* | 0.038 | 0.106 |
|  | ASV1136 | *Bacteria*-*Actinobacteriota*-*Actinobacteria*-*Corynebacteriales*-*Mycobacteriaceae*-*Mycobacterium*-unclassified | < 0.001 | 0.010 |
|  | ASV1154 | *Bacteria*-*Proteobacteria*-*Alphaproteobacteria*-*Sphingomonadales*-*Sphingomonadaceae*-*Sphingopyxis*-unclassified | 0.001 | 0.021 |
|  | ASV176 | *Bacteria*-*Proteobacteria*-*Alphaproteobacteria*-*Caulobacterales*-*Caulobacteraceae*-*Asticcacaulis*-uncultured | 0.002 | 0.117 |
|  | ASV72 | *Bacteria*-*Proteobacteria*-*Alphaproteobacteria*-*Rhizobiales*-*Beijerinckiaceae*-*Methylobacterium*-*Methylorubrum*-unclassified | 0.005 | 0.008 |
|  | ASV1168 | *Bacteria*-*Bacteroidota*-*Bacteroidia*-*Chitinophagales*-*Chitinophagaceae*-*Chitinophaga*-*Chitinophaga*_sp._*Po14a* | 0.013 | 0.026 |
|  | ASV98 | *Bacteria*-*Proteobacteria*-*Alphaproteobacteria*-*Caulobacterales*-*Caulobacteraceae*-*Phenylobacterium*-unclassified | 0.017 | 0.142 |
|  | ASV1092 | *Bacteria*-*Armatimonadota*-*Armatimonadia*-*Armatimonadales*-uncultured | 0.023 | 0.207 |
|  | ASV1167 | *Bacteria*-*Proteobacteria*-*Alphaproteobacteria*-*Rhizobiales*-*Beijerinckiaceae*-*Bosea*-unclassified | 0.013 | 0.113 |
|  | ASV394 | *Bacteria*-*Myxococcota*-*Myxococcia*-*Myxococcales*-*Myxococcaceae*-unclassified | 0.013 | 0.209 |
|  | ASV30 | *Bacteria*-*Bacteroidota*-*Bacteroidia*-*Flavobacteriales*-*Flavobacteriaceae*-*Flavobacterium*-*Flavobacterium*_sp._*WG1* | 0.003 | 0.014 |
|  | ASV273 | *Bacteria*-*Proteobacteria*-*Gammaproteobacteria*-*Burkholderiales*-*Oxalobacteraceae*-*Massilia*-unclassified | 0.004 | 0.648 |

**Continued Tables S10** Differences between inoculated and non-inoculated *F.* *falciforme* ASVs.

| Plant compartment | ASVs ID | Taxonomic position | *P*1 value  (log_2_FC ≥ 1) | *P*2 value  (log_2_FC ≥ 1) |
| --- | --- | --- | --- | --- |
| Root endosphere | ASV1196 | *Bacteria*-*Verrucomicrobiota*-*Chlamydiae*-*Chlamydiales*-uncultured | < 0.001 | 0.135 |
|  | ASV1143 | *Bacteria*-*Proteobacteria*-*Gammaproteobacteria*-*Gammaproteobacteria_Incertae_Sedis*-unclassified | 0.004 | 0.029 |
|  | ASV92 | *Bacteria*-*Bacteroidota*-*Bacteroidia*-*Cytophagales*-*Spirosomaceae*-*Dyadobacter*-unclassified | < 0.001 | 0.015 |
|  | ASV73 | *Bacteria*-*Proteobacteria*-*Alphaproteobacteria*-*Rhizobiales*-*Beijerinckiaceae*-*Bosea*-unclassified | < 0.001 | 0.015 |
|  | ASV77 | *Bacteria*-*Proteobacteria*-*Alphaproteobacteria*-*Rhizobiales*-*Xanthobacteraceae*-unclassified | 0.003 | 0.071 |
|  | ASV1 | *Fungi*-*Ascomycota*-*Sordariomycetes*-*Hypocreales*-*Nectriaceae*-*Neocosmospora*-*Neocosmospora_rubicola* | < 0.001 | < 0.001 |
| Leaf endosphere | ASV24 | *Fungi*-*Ascomycota*-*Sordariomycetes*-*Hypocreales*-*Nectriaceae*-*Fusarium*-unclassified | 0.023 | - |

*P* values are the differences in the performance of inoculated and non-inoculated ASVs on *F.* *falciforme*-tolerant (*P*1) and *F.* *falciforme*-sensitive genotype (*P*2), with *P* < 0.05 representing a significant difference between inoculated and non-inoculated *F.* *falciforme* ASVs.

**Tables S11** Significantly enriched ASVs in tolerant (T) compared to sensitive (S) soybeans with *F.* *falciforme* (+F) inoculation.

| Plant compartment | ASVs ID | Taxonomic position | *P* value (log_2_FC ≥ 1) |
| --- | --- | --- | --- |
| Rhizosphere | ASV329 | *Bacteria*-*Firmicutes*-*Bacilli*-*Bacillales*-*Bacillaceae*-*Bacillus*-unclassified | 0.016 |
|  | ASV69 | *Bacteria*-*Proteobacteria*-*Gammaproteobacteria*-*Burkholderiales*-*Oxalobacteraceae*-*Duganella*-unclassified | < 0.001 |
|  | ASV569 | *Bacteria*-*Proteobacteria*-*Alphaproteobacteria*-*Rhizobiales*-*Beijerinckiaceae*-*Microvirga*-unclassified | 0.001 |
|  | ASV874 | *Bacteria*-*Firmicutes*-*Bacilli*-*Paenibacillales*-*Paenibacillaceae*-*Thermobacillus*-uncultured | 0.026 |
|  | ASV163 | *Bacteria*-*Bacteroidota*-*Bacteroidia*-*Sphingobacteriales*-*Sphingobacteriaceae*-*Mucilaginibacter*-unclassified | 0.047 |
|  | ASV424 | *Bacteria*-*Proteobacteria*-*Gammaproteobacteria*-*Burkholderiales*-*Oxalobacteraceae*-unclassified | < 0.001 |
|  | ASV785 | *Bacteria*-*Bacteroidota*-*Bacteroidia*-*Flavobacteriales*-*Flavobacteriaceae*-*Flavobacterium*-unclassified | 0.014 |
|  | ASV259 | *Bacteria*-*Firmicutes*-*Bacilli*-*Bacillales*-*Bacillaceae*-*Bacillus*-unclassified | 0.005 |
|  | ASV14 | *Bacteria*-*Proteobacteria*-*Alphaproteobacteria*-*Sphingomonadales*-*Sphingomonadaceae*-*Novosphingobium*-unclassified | < 0.001 |
|  | ASV634 | *Bacteria*-*Proteobacteria*-*Gammaproteobacteria*-*Burkholderiales*-*Comamonadaceae*-unclassified | 0.009 |
|  | ASV499 | *Bacteria*-*Proteobacteria*-*Gammaproteobacteria*-*Burkholderiales*-*Comamonadaceae*-*Ramlibacter*-unclassified | 0.001 |
|  | ASV98 | *Bacteria*-*Proteobacteria*-*Alphaproteobacteria*-*Caulobacterales*-*Caulobacteraceae*-*Phenylobacterium*-unclassified | < 0.001 |
|  | ASV597 | *Bacteria*-*Proteobacteria*-*Alphaproteobacteria*-*Rhizobiales*-*Rhizobiales_Incertae_Sedis*-*Bauldia*-unclassified | 0.042 |
|  | ASV408 | *Bacteria*-*Proteobacteria*-*Gammaproteobacteria*-*Burkholderiales*-*Oxalobacteraceae*-unclassified | 0.026 |
|  | ASV746 | *Bacteria*-*Myxococcota*-*Polyangia*-*Haliangiales*-*Haliangiaceae*-*Haliangium*-unclassified | 0.019 |

**Continued Tables S11** Significantly enriched ASVs in tolerant (T) compared to sensitive (S) soybeans with *F.* *falciforme* (+F) inoculation.

| Plant compartment | ASVs ID | Taxonomic position | *P* value (log_2_FC ≥ 1) |
| --- | --- | --- | --- |
| Rhizosphere | ASV1011 | *Bacteria*-*Armatimonadota*-*Chthonomonadetes*-*Chthonomonadales*-*Chthonomonadaceae*-*Chthonomonas*-uncultured | 0.008 |
|  | ASV1011 | *Bacteria*-*Armatimonadota*-*Chthonomonadetes*-*Chthonomonadales*-*Chthonomonadaceae*-*Chthonomonas*-uncultured | 0.008 |
|  | ASV807 | *Bacteria*-*Cyanobacteria*-*Sericytochromatia*-uncultured | 0.008 |
|  | ASV821 | *Bacteria*-*Gemmatimonadota*-*Gemmatimonadetes*-*Gemmatimonadales*-*Gemmatimonadaceae*-*Gemmatimonas*-unclassified | 0.002 |
|  | ASV572 | *Bacteria*-*Actinobacteriota*-*Actinobacteria*-*Kineosporiales*-*Kineosporiaceae*-unclassified | 0.001 |
|  | ASV25 | *Bacteria*-*Proteobacteria*-*Alphaproteobacteria*-*Azospirillales*-*Azospirillaceae*-*Azospirillum*-unclassified | 0.009 |
|  | ASV726 | *Bacteria*-*Firmicutes*-*Bacilli*-*Thermoactinomycetales*-*Thermoactinomycetaceae*-*Planifilum*-unclassified | 0.046 |
|  | ASV895 | *Bacteria*-unclassified | 0.032 |
|  | ASV72 | *Fungi*-*Ascomycota*-*Eurotiomycetes*-*Eurotiales*-*Aspergillaceae*-*Penicillium*-*Penicillium_oxalicum* | 0.001 |
|  | ASV243 | *Fungi*-unclassified | 0.039 |
| Root endosphere | ASV31 | *Bacteria*-*Proteobacteria*-*Alphaproteobacteria*-*Rhizobiales*-*Beijerinckiaceae*-*Bosea*-unclassified | 0.009 |
|  | ASV69 | *Bacteria*-*Proteobacteria*-*Gammaproteobacteria*-*Burkholderiales*-*Oxalobacteraceae*-*Duganella*-unclassified | 0.023 |
|  | ASV144 | *Bacteria*-*Proteobacteria*-*Gammaproteobacteria*-*Burkholderiales*-*Comamonadaceae*-*Roseateles*-unclassified | 0.029 |
|  | ASV1204 | *Bacteria*-*Bacteroidota*-*Bacteroidia*-*Chitinophagales*-*Chitinophagaceae*-*Rurimicrobium*-*Rurimicrobium_arvi* | 0.031 |
|  | ASV308 | *Bacteria*-*Proteobacteria*-*Alphaproteobacteria*-*Rhizobiales*-*Rhizobiaceae*-*Mesorhizobium*-unclassified | 0.002 |
|  | ASV10 | *Bacteria*-*Proteobacteria*-*Alphaproteobacteria*-*Rhizobiales*-*Rhizobiaceae*-*Allorhizobium*-*Neorhizobium*-*Pararhizobium*-*Rhizobium*-unclassified | 0.017 |

**Continued Tables S11** Significantly enriched ASVs in tolerant (T) compared to sensitive (S) soybeans with *F.* *falciforme* (+F) inoculation.

| Plant compartment | ASVs ID | Taxonomic position | *P* value (log_2_FC ≥ 1) |
| --- | --- | --- | --- |
| Root endosphere | ASV84 | *Bacteria*-*Proteobacteria*-*Alphaproteobacteria*-*Sphingomonadales*-*Sphingomonadaceae*-*Novosphingobium*-unclassified | 0.025 |
|  | ASV1183 | *Bacteria*-*Proteobacteria*-*Alphaproteobacteria*-*Rhizobiales*-unclassified | 0.008 |
|  | ASV1135 | *Bacteria*-*Proteobacteria*-*Alphaproteobacteria*-*Sphingomonadales*-*Sphingomonadaceae*-*Novosphingobium*-unclassified | < 0.001 |
|  | ASV224 | *Bacteria*-*Proteobacteria*-*Alphaproteobacteria*-*Rhizobiales*-*Rhizobiaceae*-*Mesorhizobium*-unclassified | 0.008 |
|  | ASV55 | *Bacteria*-*Bacteroidota*-*Bacteroidia*-*Sphingobacteriales*-*Sphingobacteriaceae*-*Mucilaginibacter*-unclassified | 0.030 |
|  | ASV34 | *Bacteria*-*Actinobacteriota*-*Actinobacteria*-*Micrococcales*-*Micrococcaceae*-*Arthrobacter*-unclassified | 0.046 |
|  | ASV1241 | *Bacteria*-*Verrucomicrobiota*-*Verrucomicrobiae*-*Verrucomicrobiales*-*Rubritaleaceae*-*Luteolibacter*-unclassified | 0.029 |
|  | ASV1200 | *Bacteria*-*Proteobacteria*-*Alphaproteobacteria*-*Sphingomonadales*-*Sphingomonadaceae*-*Sphingopyxis*-unclassified | 0.028 |
|  | ASV53 | *Bacteria*-*Proteobacteria*-*Alphaproteobacteria*-*Caulobacterales*-*Caulobacteraceae*-*Caulobacter*-unclassified | 0.007 |
|  | ASV56 | *Bacteria*-*Proteobacteria*-*Alphaproteobacteria*-*Sphingomonadales*-*Sphingomonadaceae*-*Novosphingobium*-unclassified | 0.007 |
|  | ASV1136 | *Bacteria*-*Actinobacteriota*-*Actinobacteria*-*Corynebacteriales*-*Mycobacteriaceae*-*Mycobacterium*-unclassified | 0.003 |
|  | ASV1145 | *Bacteria*-*Bacteroidota*-*Bacteroidia*-*Chitinophagales*-*Chitinophagaceae*-*Niastella*-*Niastella_koreensis_GR20-10* | 0.035 |
|  | ASV14 | *Bacteria*-*Proteobacteria*-*Alphaproteobacteria*-*Sphingomonadales*-*Sphingomonadaceae*-*Novosphingobium*-unclassified | < 0.001 |
|  | ASV1154 | *Bacteria*-*Proteobacteria*-*Alphaproteobacteria*-*Sphingomonadales*-*Sphingomonadaceae*-*Sphingopyxis*-unclassified | 0.002 |
|  | ASV176 | *Bacteria*-*Proteobacteria*-*Alphaproteobacteria*-*Caulobacterales*-*Caulobacteraceae*-*Asticcacaulis*-uncultured | < 0.001 |

**Continued Tables S11** Significantly enriched ASVs in tolerant (T) compared to sensitive (S) soybeans with *F.* *falciforme* (+F) inoculation.

| Plant compartment | ASVs ID | Taxonomic position | *P* value (log_2_FC ≥ 1) |
| --- | --- | --- | --- |
| Root endosphere | ASV113 | *Bacteria*-*Acidobacteriota*-*Acidobacteriae*-*Acidobacteriales*-*Acidobacteriaceae-Subgroup_1-Edaphobacter*-uncultured | 0.036 |
|  | ASV968 | *Bacteria*-*Patescibacteria*-*Saccharimonadia*-*Saccharimonadales*-*Saccharimonadaceae*-*TM7a*-uncultured | 0.002 |
|  | ASV273 | *Bacteria*-*Proteobacteria*-*Gammaproteobacteria*-*Burkholderiales*-*Oxalobacteraceae*-*Massilia*-unclassified | 0.001 |
|  | ASV32 | *Bacteria*-*Proteobacteria*-*Gammaproteobacteria*-*Burkholderiales*-*Comamonadaceae*-unclassified | 0.001 |
|  | ASV25 | *Bacteria*-*Proteobacteria*-*Alphaproteobacteria*-*Azospirillales*-*Azospirillaceae*-*Azospirillum*-unclassified | 0.035 |
|  | ASV170 | *Bacteria*-*Proteobacteria*-*Gammaproteobacteria*-*Burkholderiales*-*Comamonadaceae*-unclassified | < 0.001 |
|  | ASV1140 | *Bacteria*-*Proteobacteria*-*Alphaproteobacteria*-*Sphingomonadales*-*Sphingomonadaceae*-unclassified | 0.003 |
| Leaf endosphere | ASV264 | *Fungi*-*Ascomycota*-*Leotiomycetes*-*Erysiphales*-*Erysiphaceae*-*Erysiphe*-*Erysiphe_diffusa* | < 0.001 |
|  | ASV24 | *Fungi*-*Ascomycota*-*Sordariomycetes*-*Hypocreales*-*Nectriaceae*-*Fusarium*-unclassified | 0.029 |

*P* < 0.05 represents a significant difference.

**Table S12** Taxonomic and species information about the inhibitory isolates.

| ID | Antagonistic phenotypes against *F.* *falciforme* | Strain assignment-baseon16S rRNA sequence and ITS sequencing |
| --- | --- | --- |
| Re-B1 | Antimicrobial compound-mediated inhibition | *Bacteria*; *Firmicutes*; *Bacilli*; *Bacillales*; *Bacillaceae*; *Bacillus*; *Bacillus siamensis* |
| Re-B2 | Antimicrobial compound-mediated inhibition | *Bacteria*; *Firmicutes*; *Bacilli*; *Bacillales*; *Bacillaceae*; *Caldibacillus*; *Caldibacillus hisashii* |
| Re-B3 | Antimicrobial compound-mediated inhibition | *Bacteria*; *Firmicutes*; *Bacilli*; *Bacillales*; *Bacillaceae*; *Bacillus*; *Bacillus amyloliquefaciens* |
| Re-B4 | Antimicrobial compound-mediated inhibition | *Bacteria*; *Firmicutes*; *Bacilli*; *Bacillales*; *Bacillaceae*; *Bacillus*; *Bacillus tequilensis* |
| BLT62 | Antimicrobial compound-mediated inhibition | *Bacteria*; *Firmicutes*; *Bacilli*; *Bacillales*; *Bacillaceae*; *Bacillus*; *Bacillus velezensis* |
| Re-F1 | Antimicrobial compound-mediated inhibition | *Fungi*; *Ascomycota*; *Saccharomyceta*; *Pezizomycotina*; *Eurotiomycetes*; *Penicillium*; *Penicillium thomii* |
| Re-F2 | Antimicrobial compound-mediated inhibition | *Fungi*; *Ascomycota*; *Eurotiomycetes*; *Eurotiales*; *Trichocomaceae*; *Talaromyces*; *Talaromyces* *funiculosus* |
| FSS5 | Preemptive inhibition | *Fungi*; *Basidiomycota*; *Tremellomycetes*; *Tremellales*; *Rhynchogastremataceae*; *Papiliotrema*; *Papiliotrema laurentii* |
| FLT19 | Preemptive inhibition | *Fungi*; *Ascomycota*; *Dothideomycetes*; *Cladosporiales*; *Cladosporiaceae*; *Cladosporium*; *Cladosporium tenuissimum* |
| FLT26 | Preemptive inhibition | *Fungi*; *Ascomycota*; *Dothideomycetes*; *Cladosporiales*; *Cladosporiaceae*; *Cladosporium*; unclassified *Cladosporium* |
| FLT42 | Preemptive inhibition | *Fungi*; *Ascomycota*; *Dothideomycetes*; *Cladosporiales*; *Cladosporiaceae*; *Cladosporium*; *Cladosporium* sp. *4 YHY-2018* |
| FLT4 | Preemptive inhibition | *Fungi*; *Ascomycota*; *Dothideomycetes*; *Cladosporiales*; *Cladosporiaceae*; *Cladosporium*; *Cladosporium cladosporioides* |
| FLS6 | Preemptive inhibition | *Fungi*; *Ascomycota*; *Dothideomycetes*; *Cladosporiales*; *Cladosporiaceae*; *Cladosporium*; *Cladosporium caricinum* |
| FLT21 | Preemptive inhibition | *Fungi*; *Ascomycota*; *Dothideomycetes*; *Cladosporiales*; *Cladosporiaceae*; *Cladosporium*; unclassified *Cladosporium* |
| FLS57 | Antimicrobial compound-mediated inhibition | *Fungi*; *Ascomycota*; *Sordariomycetes*; *Hypocreales*; *Nectriaceae*; *Fusarium*; *Fusarium proliferatum* |

**Continued Table S12** Taxonomic and species information about the inhibitory isolates.

| ID | Antagonistic phenotypes against *F.* *falciforme* | Strain assignment-baseon16S rRNA sequence and ITS sequencing |
| --- | --- | --- |
| FLS66 | Preemptive inhibition | *Fungi*; *Ascomycota*; *Sordariomycetes*; *Hypocreales*; *Stachybotryaceae*; *Xenomyrothecium*; *Xenomyrothecium tongaense* |
| FLS33 | Antimicrobial compound-mediated inhibition | *Fungi*; *Ascomycota*; *Sordariomycetes*; *Xylariales*; *Apiosporaceae*; *Apiospora*; *Apiospora* *sphaerosperma* |
| FLT9 | Preemptive inhibition | *Fungi*; *Ascomycota*; *Sordariomycetes*; *Xylariales*; *Apiosporaceae*; *Nigrospora*; unclassified *Nigrospora* |
| FLT15 | Preemptive inhibition | *Fungi*; *Ascomycota*; *Sordariomycetes*; *Xylariales*; *Apiosporaceae*; *Nigrospora*; unclassified *Nigrospora* |

**Table S13** Taxonomic and species information about the strains of SynComs.

| Plant compartment | SynCom ID | Selected strains ID | Selected strains taxonomy |
| --- | --- | --- | --- |
| Rhizosphere | SynCom 1 | BS1 | *Bacteria*; *Firmicutes*; *Bacilli*; *Bacillales*; *Bacillaceae*; *Bacillus*; *Bacillus tropicus* |
|  |  | BS2 | *Bacteria*; *Firmicutes*; *Bacilli*; *Bacillales*; *Bacillaceae*; *Bacillus*; *Bacillus paramycoides* |
|  |  | BS3 | *Bacteria*; *Firmicutes*; *Bacilli*; *Bacillales*; *Bacillaceae*; *Bacillus*; *Bacillus cereus* |
|  |  | BS4 | *Bacteria*; *Firmicutes*; *Bacilli*; *Bacillales*; *Bacillaceae*; *Bacillus*; *Bacillus toyonensis* |
|  |  | BS5 | *Bacteria*; *Firmicutes*; *Bacilli*; *Bacillales*; *Bacillaceae*; *Bacillus*; *Bacillus wiedmannii* |
|  |  | BS6 | *Bacteria*; *Firmicutes*; *Bacilli*; *Bacillales*; *Paenibacillaceae*; *Brevibacillus*; *Brevibacillus massiliensis* |
|  |  | BS7 | *Bacteria*; *Actinobacteriota*; *Actinobacteria*; *Streptomycetales*; *Streptomycetaceae*; *Streptomyces*; *Streptomyces ardesiacus* |
|  |  | BS8 | *Bacteria*; *Proteobacteria*; *Gammaproteobacteria*; *Burkholderiales*; *Oxalobacteraceae*; *Massilia*; *Massilia oculi* |
|  |  | BS9 | *Bacteria*; *Proteobacteria*; *Gammaproteobacteria*; *Burkholderiales*; *Burkholderiaceae*; *Pandoraea*; *Pandoraea commovens* |
|  | SynCom 2 | FS1 | *Fungi*; *Ascomycota*; *Eurotiomycetes*; *Eurotiales*; *Aspergillaceae*; *Penicillium*; unclassified *Penicillium* |
|  |  | FS2 | *Fungi*; *Ascomycota*; *Eurotiomycetes*; *Eurotiales*; *Aspergillaceae*; *Aspergillus*; unclassified *Aspergillus* |
|  | SynCom 3 | BS1 | *Bacteria*; *Firmicutes*; *Bacilli*; *Bacillales*; *Bacillaceae*; *Bacillus*; *Bacillus tropicus* |
|  |  | BS2 | *Bacteria*; *Firmicutes*; *Bacilli*; *Bacillales*; *Bacillaceae*; *Bacillus*; *Bacillus paramycoides* |
|  |  | BS3 | *Bacteria*; *Firmicutes*; *Bacilli*; *Bacillales*; *Bacillaceae*; *Bacillus*; *Bacillus cereus* |
|  |  | BS4 | *Bacteria*; *Firmicutes*; *Bacilli*; *Bacillales*; *Bacillaceae*; *Bacillus*; *Bacillus toyonensis* |
|  |  | BS5 | *Bacteria*; *Firmicutes*; *Bacilli*; *Bacillales*; *Bacillaceae*; *Bacillus*; *Bacillus wiedmannii* |
|  |  | BS6 | *Bacteria*; *Firmicutes*; *Bacilli*; *Bacillales*; *Paenibacillaceae*; *Brevibacillus*; *Brevibacillus massiliensis* |
|  |  | BS7 | *Bacteria*; *Actinobacteriota*; *Actinobacteria*; *Streptomycetales*; *Streptomycetaceae*; *Streptomyces*; *Streptomyces ardesiacus* |
|  |  | BS8 | *Bacteria*; *Proteobacteria*; *Gammaproteobacteria*; *Burkholderiales*; *Oxalobacteraceae*; *Massilia*; *Massilia oculi* |
|  |  | BS9 | *Bacteria*; *Proteobacteria*; *Gammaproteobacteria*; *Burkholderiales*; *Burkholderiaceae*; *Pandoraea*; *Pandoraea commovens* |
|  |  | FS1 | *Fungi*; *Ascomycota*; *Eurotiomycetes*; *Eurotiales*; *Aspergillaceae*; *Penicillium*; unclassified *Penicillium* |
|  |  | FS2 | *Fungi*; *Ascomycota*; *Eurotiomycetes*; *Eurotiales*; *Aspergillaceae*; *Aspergillus*; unclassified *Aspergillus* |

**Table S13 (continued)** Taxonomic and species information about the strains of SynComs.

| Plant compartment | Syncom ID | Selected strains ID | Selected strains taxonomy |
| --- | --- | --- | --- |
| Root  endosphere | SynCom 4 | BR1 | *Bacteria*; *Firmicutes*; *Bacilli*; *Bacillales*; *Bacillaceae*; *Bacillus*; *Bacillus safensis* |
|  |  | BR2 | *Bacteria*; *Firmicutes*; *Bacilli*; *Bacillales*; *Bacillaceae*; *Bacillus*; *Bacillus nitroreducens* |
|  |  | BR3 | *Bacteria*; *Firmicutes*; *Bacilli*; *Bacillales*; *Bacillaceae*; *Bacillus*; *Bacillus LJIY_s* |
|  |  | BR4 | *Bacteria*; *Firmicutes*; *Bacilli*; *Bacillales*; *Bacillaceae*; *Bacillus*; *Bacillus tequilensis* |
|  |  | BR5 | *Bacteria*; *Proteobacteria*; *Gammaproteobacteria*; *Burkholderiales*; *Oxalobacteraceae*; *Massilia*; *Massilia* *oculi* |
|  |  | BR6 | *Bacteria*; *Firmicutes*; *Bacilli*; *Bacillales*; *Paenibacillaceae*; *Paenibacillus*; *Paenibacillus cineris* |
|  |  | BR7 | *Bacteria*; *Proteobacteria*; *Betaproteobacteria*; *Burkholderiales*; *Herbaspirillaceae*; *Herbaspirillum*; *Herbaspirillum aquaticum* |
|  | SynCom 5 | FR1 | *Fungi*; *Ascomycota*; *Eurotiomycetes*; *Eurotiales*; *Aspergillaceae*; *Penicillium*; unclassified *Penicillium* |
|  |  | FR2 | *Fungi*; *Ascomycota*; *Dothideomycetes*; *Cladosporiales*; *Cladosporiaceae*; *Cladosporium*; *Cladosporium tenuissimum* |
|  | SynCom 6 | BR1 | *Bacteria*; *Firmicutes*; *Bacilli*; *Bacillales*; *Bacillaceae*; *Bacillus*; *Bacillus safensis* |
|  |  | BR2 | *Bacteria*; *Firmicutes*; *Bacilli*; *Bacillales*; *Bacillaceae*; *Bacillus*; *Bacillus nitroreducens* |
|  |  | BR3 | *Bacteria*; *Firmicutes*; *Bacilli*; *Bacillales*; *Bacillaceae*; *Bacillus*; *Bacillus LJIY_s* |
|  |  | BR4 | *Bacteria*; *Firmicutes*; *Bacilli*; *Bacillales*; *Bacillaceae*; *Bacillus*; *Bacillus tequilensis* |
|  |  | BR5 | *Bacteria*; *Proteobacteria*; *Gammaproteobacteria*; *Burkholderiales*; *Oxalobacteraceae*; *Massilia*; *Massilia* *oculi* |
|  |  | BR6 | *Bacteria*; *Firmicutes*; *Bacilli*; *Bacillales*; *Paenibacillaceae*; *Paenibacillus*; *Paenibacillus cineris* |
|  |  | BR7 | *Bacteria*; *Proteobacteria*; *Betaproteobacteria*; *Burkholderiales*; *Herbaspirillaceae*; *Herbaspirillum*; *Herbaspirillum aquaticum* |
|  |  | FR1 | *Fungi*; *Ascomycota*; *Eurotiomycetes*; *Eurotiales*; *Aspergillaceae*; *Penicillium*; unclassified *Penicillium* |
|  |  | FR2 | *Fungi*; *Ascomycota*; *Dothideomycetes*; *Cladosporiales*; *Cladosporiaceae*; *Cladosporium*; *Cladosporium tenuissimum* |

**Table S13 (continued)** Taxonomic and species information about the strains of SynComs.

| Plant compartment | Syncom ID | Selected strains ID | Selected strains taxonomy |
| --- | --- | --- | --- |
| Leaf endosphere | SynCom 7 | BL1 | *Bacteria*; *Proteobacteria*; *Gammaproteobacteria*; *Burkholderiales*; *Burkholderiaceae*; *Pandoraea*; *Pandoraea commovens* |
|  |  | BL2 | *Bacteria*; *Proteobacteria*; *Gammaproteobacteria*; *Burkholderiales*; *Burkholderiaceae*; *Pandoraea*; *Pandoraea morbifera* |
|  | SynCom 8 | FL1 | *Fungi*; *Ascomycota*; *Eurotiomycetes*; *Eurotiales*; *Aspergillaceae*; *Penicillium*; unclassified *Penicillium* |
|  |  | FL2 | *Fungi*; *Ascomycota*; *Sordariomycetes*; *Trichosphaeriales*; *Trichosphaeriaceae*; *Nigrospora*; unclassified *Nigrospora* |
|  |  | FL3 | *Fungi*; *Ascomycota*; *Eurotiomycetes*; *Eurotiales*; *Aspergillaceae*; *Aspergillus*; unclassified *Aspergillus* |
|  | SynCom 9 | BL1 | *Bacteria*; *Proteobacteria*; *Gammaproteobacteria*; *Burkholderiales*; *Burkholderiaceae*; *Pandoraea*; *Pandoraea commovens* |
|  |  | BL2 | *Bacteria*; *Proteobacteria*; *Gammaproteobacteria*; *Burkholderiales*; *Burkholderiaceae*; *Pandoraea*; *Pandoraea morbifera* |
|  |  | FL1 | *Fungi*; *Ascomycota*; *Eurotiomycetes*; *Eurotiales*; *Aspergillaceae*; *Penicillium*; unclassified *Penicillium* |
|  |  | FL2 | *Fungi*; *Ascomycota*; *Sordariomycetes*; *Trichosphaeriales*; *Trichosphaeriaceae*; *Nigrospora*; unclassified *Nigrospora* |
|  |  | FL3 | *Fungi*; *Ascomycota*; *Eurotiomycetes*; *Eurotiales*; *Aspergillaceae*; *Aspergillus*; unclassified *Aspergillus* |

**Table S13 (continued)** Taxonomic and species information about the strains of SynComs.

| Plant compartment | Syncom ID | Selected strains ID | Selected strains taxonomy |
| --- | --- | --- | --- |
| Rhizosphere /  Root endosphere /  Leaf endosphere | SynCom 10 | Re-B1 | *Bacteria*; *Firmicutes*; *Bacilli*; *Bacillales*; *Bacillaceae*; *Bacillus*; *Bacillus siamensis* |
|  |  | Re-B2 | *Bacteria*; *Firmicutes*; *Bacilli*; *Bacillales*; *Bacillaceae*; *Caldibacillus*; *Caldibacillus hisashii* |
|  |  | Re-B3 | *Bacteria*; *Firmicutes*; *Bacilli*; *Bacillales*; *Bacillaceae*; *Bacillus*; *Bacillus amyloliquefaciens* |
|  |  | Re-B4 | *Bacteria*; *Firmicutes*; *Bacilli*; *Bacillales*; *Bacillaceae*; *Bacillus*; *Bacillus tequilensis* |
|  | SynCom 11 | Re-F1 | *Fungi*; *Ascomycota*; *saccharomyceta*; *Pezizomycotina*; *Eurotiomycetes*;*Penicillium*; *Penicillium thomii* |
|  |  | Re-F2 | *Fungi*; *Ascomycota*; *Eurotiomycetes*; *Eurotiales*; *Trichocomaceae*; *Talaromyces*; *Talaromyces funiculosus* |
|  | SynCom 12 | Re-B1 | *Bacteria*; *Firmicutes*; *Bacilli*; *Bacillales*; *Bacillaceae*; *Bacillus*; *Bacillus siamensis* |
|  |  | Re-B2 | *Bacteria*; *Firmicutes*; *Bacilli*; *Bacillales*; *Bacillaceae*; *Caldibacillus*; *Caldibacillus hisashii* |
|  |  | Re-B3 | *Bacteria*; *Firmicutes*; *Bacilli*; *Bacillales*; *Bacillaceae*; *Bacillus*; *Bacillus amyloliquefaciens* |
|  |  | Re-B4 | *Bacteria*; *Firmicutes*; *Bacilli*; *Bacillales*; *Bacillaceae*; *Bacillus*; *Bacillus tequilensis* |
|  |  | Re-F1 | *Fungi*; *Ascomycota*; *saccharomyceta*; *Pezizomycotina*; *Eurotiomycetes*;*Penicillium*; *Penicillium thomii* |
|  |  | Re-F2 | *Fungi*; *Ascomycota*; *Eurotiomycetes*; *Eurotiales*; *Trichocomaceae*; *Talaromyces*; *Talaromyces funiculosus* |

SynCom 1–9 were constructed based on species that showed significant enrichment at the genus level in different compartments of the tolerant variety upon *F. falciforme* inoculation. In contrast, SynCom 10–12 were assembled from strains that exhibited strong antagonistic activity against *F. falciforme* in in vitro plate assays. Notably, SynCom 10–12 share an identical strain composition across the rhizosphere, root endosphere, and leaf endosphere. To evaluate their biocontrol efficacy against soybean *F. falciforme* root rot, SynCom 10–12 were respectively inoculated into each of these three compartments.

[1] Jin X, Jia H, Ran L. et al. Fusaric acid mediates the assembly of disease-suppressive rhizosphere microbiota via induced shifts in plant root exudates. *Nat Commun* 2024;**15**:5125. [10.1038/s41467-024-49218-9](https://doi.org/10.1038/s41467-024-49218-9)

[2] Jiang G, Zhang Y, Chen M. et al. Effects of plant tissue permeability on invasion and population bottlenecks of a phytopathogen. *Nat Commun* 2024;**15**:62. [10.1038/s41467-023-44234-7](https://doi.org/10.1038/s41467-023-44234-7)

[3] Caporaso JG, Lauber CL, Walters WA. et al. Global patterns of 16S rRNA diversity at a depth of millions of sequences per sample. *PNAS* 2011;**108 (1)**:4516–4522. 10.1073/pnas.1000080107

[4] Li S, Deng Y, Wang Z. et al. Exploring the accuracy of amplicon-based internal transcribed spacer markers for a fungal community. *Mol Ecol Resour* 2020;**20**:170–184. 10.1111/1755-0998.13097

[5] Johnson JS, Spakowicz DJ, Hong BY. et al. Evaluation of 16S rRNA gene sequencing for species and strain-level microbiome analysis. *Nat Commun* 2019;**10**:5029. 10.1038/s41467-019-13036-1

[6] White TJ. Amplification and direct sequencing of fungal ribosomal RNA genes for phylogenetics. In:PCR Protocols, a Guide to Methods and Applications 1990;**3**:315-322. 10.1016/B978-0-12-372180-8.50042-1
